# Identification of proprotein convertase substrates using genome-wide expression correlation analysis

**DOI:** 10.1186/1471-2164-12-618

**Published:** 2011-12-20

**Authors:** Hannu Turpeinen, Sampo Kukkurainen, Kati Pulkkinen, Timo Kauppila, Kalle Ojala, Vesa P Hytönen, Marko Pesu

**Affiliations:** 1 Immunoregulation, Institute of Biomedical Technology, FI-33014 University of Tampere, Finland; 2 BioMediTech, Tampere, Finland; 3 Protein Dynamics, Institute of Biomedical Technology, University of Tampere, Finland; 4 Institute for Molecular Medicine Finland (FIMM), University of Helsinki, Helsinki, Finland; 5 Centre for Laboratory Medicine, Tampere University Hospital, Finland

## Abstract

**Background:**

Subtilisin/kexin-like proprotein convertase (PCSK) enzymes have important regulatory function in a wide variety of biological processes. PCSKs proteolytically process at a target sequence that contains basic amino acids arginine and lysine, which results in functional maturation of the target protein. *In vitro *assays have showed significant biochemical redundancy between the seven family members, but the phenotypes of PCSK deficient mice and patients carrying an inactive PCSK allele argue for a specific biological function. Modeling the structures of individual PCSK enzymes has offered little insights into the specificity determinants. However, previous studies have shown that there can be a coordinated expression between a PCSK and its target molecule. Here, we have surveyed the putative PCSK target proteins using genome-wide expression correlation analysis and cleavage site prediction algorithms.

**Results:**

We first performed a gene expression correlation analysis over the whole genome for all PCSK enzymes. PCSKs were found to cluster differently based on the strength of correlations. The screen for putative PCSK target proteins showed a significant enrichment (p-values from 1.2e-4 to < 1.0e-10) of putative targets among the most positively correlating genes for most PCSKs. Interestingly, there was no enrichment in putative targets among the genes that correlated positively with the biologically redundant PCSK7, whereas PCSK5 showed an inverse correlation. PCSKs also showed a highly variable degree of shared target genes that were identified by expression correlation and cleavage site prediction. Multiple alignments were used to evaluate the putative targets to pinpoint the important residues for the substrate recognition. Finally, we validated our approach and identified biochemically PAPPA1 and ADAMTS6 as novel targets for FURIN proteolytic activity.

**Conclusions:**

Most PCSK enzymes display strong positive expression correlation with predicted target proteins in our genome-wide analysis. We also show that expression correlation screen combined with a cleavage site-prediction analysis can be used to identify novel bona fide target molecules for PCSKs. Exploring the positively correlating genes can thus offer additional insights into the biology of proprotein convertases.

## Background

Many proteins that control biological processes are initially synthesized as immature proproteins, which need to be proteolytically converted into functional end products. This proprotein conversion dictates the bioavailability of these dormant molecules. Therefore the enzymes responsible of this event, proprotein convertases (PCSK), are important regulatory factors. The primarily identified seven PCSKs (PCSK1-2, FURIN, PCSK4-7) are closely related and evolutionarily conserved subtilisin/kexin-like serine proteases that process their targets mainly in the secretory pathway, cell surface and endosomes (reviewed in [1,2]). The general PCSK target sequence typically encompasses a series of basic amino acids lysine and/or arginine; (K/R)-(X)n-(K/R)↓, where n is 0, 2, 4 or 6 and X is any amino acid. More recently identified and distantly related PCSK family members MBTPS1 and PCSK9 do not cleave at basic amino acids. Instead, MBTPS1 targets the consensus motif (R/K)-X-(hydrophobic)-X↓, and PCSK9 has only autocatalytic cleavage activity on its prosegment sequence VFAQ_152_↓.

Understanding the determinants of PCSK target specificity is currently incomplete; convertases have shown a variable degree of redundancy in target selection in *in vitro *experiments, especially in over-expression settings [3,4]. Importantly, however, the phenotypes of PCSK deficient animals and patients with genetic mutations that result in abolished or enhanced PCSK activity show compellingly that most, if not all, family members also have specific target proteins [5]. One approach to gain insights into the specificity prerequisites is to model and compare the structures of the PCSK enzymes. Previous results suggest that all human PCSKs share a remarkably similar structure of the substrate binding groove and there are only subtle differences in the number of charged residues close to the substrate binding region [6].

Additional clues for identification of physiological PCSK - substrate pairs come from experiments that show a positive expression correlation between PCSK and its substrate in cell. For example, FURIN is co-expressed with its target molecule VEGF-C in head and neck cancer [7], and some targets, like TGFβ-1, are even known to create a feed-forward mechanism by enhancing the expression of their converting enzyme (FURIN) [8]. An explanation for the coordinated expression is often the common transcription factors that regulate the expression of both PCSK and a target molecule [9,10]. However, whether this phenomenon is universal for all PCSKs and indicative of biological substrate specificity is currently not known.

In order to find new PCSK - target molecule pairs we have here analyzed the genome-wide expression correlation for all human genes and PCSK enzymes in a very large number of samples. Our results also show that with notable exception of PCSK5 and PCSK7 the genes that are strongly co-regulated with a certain PCSK are often putative target molecules for these enzymes. We found also that PCSKs display a highly variable number of unique and shared target genes, and that they also cluster differently with regard how many genes show a strong expression correlation. We finally validate our approach in biochemical experiments and identify PAPPA1 and ADAMTS6 as novel FURIN target molecules.

## Results and Discussion

**Figure 1:**
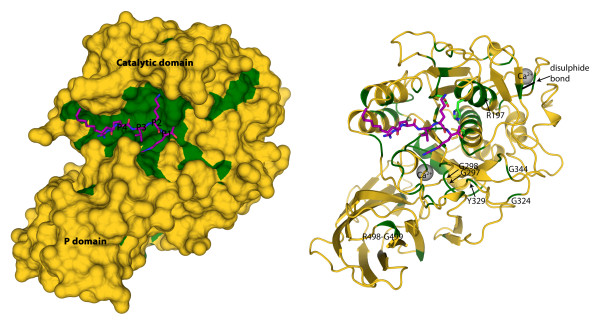
**Conservation of the PCSK catalytic domain**. The 3D-structure of mouse FURIN is shown as surface (left) and cartoon (right) presentation and the bound inhibitor as purple stick model. Amino acids identical in >95% of the 152 related PCSK sequences are marked with green color, indicating highly conserved active site. Sequences that were used for the analysis are available in the Additional File 1. Some of the conserved residues are labeled using the FURIN numbering.

The crystal structures for mouse FURIN, bacterial SUBTILISIN and yeast KEXIN have been previously determined using X-ray crystallography [11-13]. We used the mouse FURIN structure as a template to survey the conservation of enzymatically active cleft/pocket of all the PCSK homologues in different genomes (152 sequences, Figure 1, Additional File 1). We found the catalytic cleft, especially the residues that are in contact with the P1-P4 sites of the substrate, to be highly conserved in all PCSKs over different genomes. Our data further corroborate the remarkable conservation of the catalytically active cleft of the PCSK enzymes and suggest that the PCSK substrate specificity is likely not solely explained by the structural features of the active site [6].

### Expression correlation

**Figure 2:**
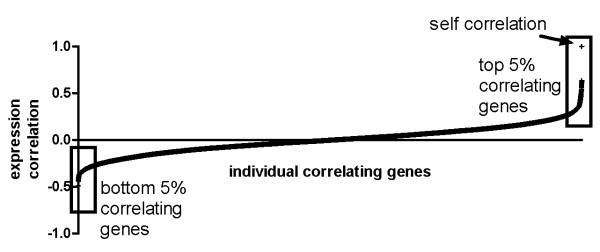
**An example picture of whole genome expression correlation with a PCSK gene**. Top 5% and bottom 5% of correlating genes were selected for further investigations.

Meta-analyses of public microarray data sources offer insights into the genome-wide gene expression in health and disease [14,15]. GeneSapiens is a comprehensive reference database of the human transcriptome that integrates large quantities of human expression data into a unified format [16]. Using GeneSapiens we first arranged the whole human transcriptome (17330 genes) according to expression similarity with PCSK genes in all healthy tissues (Figure 2, Additional File 2). The average correlation between PCSKs and all other genes was close to zero (range -0.02 - 0.04). The 5% (867 genes, 861 for PCSK4) of both most positively (hereafter "top 5%") and negatively ("bottom 5%") correlating genes were chosen for further analysis.

**Figure 3:**
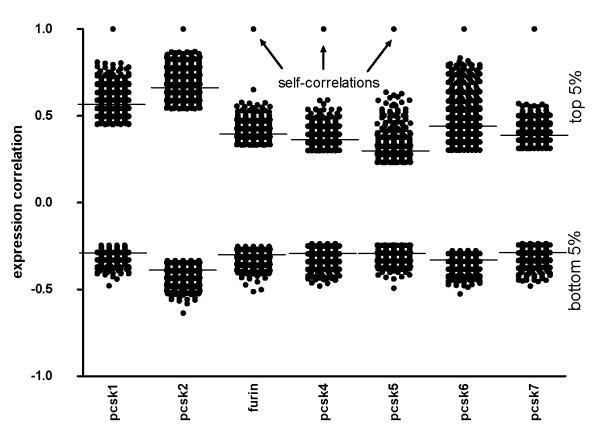
**Top 5% and bottom 5% correlating genes with PCSK genes**. An average expression correlation is shown for each group as a horizontal line.

Marked differences were observed in the magnitude of correlation amongst the top 5% genes. Average correlations fell into two distinct categories: PCSK1, PCSK2 and PCSK6 cluster together with significantly higher average correlations (0.57, 0.66 and 0.44, respectively) than FURIN (0.40), PCSK4 (0.36), PCSK5 (0.30) and PCSK7 (0.39). PCSK2 stands out from all the other PCSKs with 66 genes with an extreme high genome-wide expression correlation value of >0.8 (Additional File 2, Figure 3). In contrast, no such high correlations were observed for FURIN, PCSK4, PCSK5 or PCSK7, whereas PCSK1 and PCSK6 had only a few genes with equally high correlation (Additional File 2). The genes that had the strongest negative expression correlation with PCSKs (bottom 5% genes) showed less variation in their magnitude of correlation (average correlations in groups between -0.29 and -0.39, Figure 3).

Finally, we explored the strength of the expression correlation between the PCSK genes. Our data show that apart from PCSK1 and PCSK2 enzymes, which are chiefly present in the neuroendocrine tissues, no other PCSK-PCSK pair ranks within either top or bottom 5% in the analyses over the whole spectrum of tissues (Additional File 3). It is noteworthy, however, that when PCSK pairs were analyzed in a tissue specific setting other strong correlations can be observed. For example, FURIN and PCSK6 show highly significant correlation in blood myeloid cells (n = 156, r = 0.634, p = 0). Tissue specific expression correlation data for all PCSK pairs is shown in Additional File 4.

### Identification of putative PCSK targets

The scheme that PCSK enzymes are co-expressed with their target molecules *in vivo *is supported by experimental evidence where immature growth factors are shown to be co-expressed and even induce the expression of their converting enzyme [7,8]. We wanted to explore whether putative PCSK target molecules are generally enriched in the genes that are coordinately expressed with PCSK enzymes. To this end, we employed a previously published, artificial neural networks based method (ProP 1.0, http://www.cbs.dtu.dk/services/ProP/, [17]) to survey PCSK target sequences in the most positively and negatively co-expressed genes. In addition, since PCSK mostly process their target proteins in the secretory pathway, the presence of signal peptide sequence predicted using the SignalP algorithm integrated in ProP 1.0 was used as an additional inclusion criterion for putative targets [18].

Our analysis show that with the exception of PCSK5 and PCSK7 the top 5% of positively correlating gene pools encompasses a significant enrichment of putative target genes when compared with the bottom 5% correlates (Table 1, Additional File 2 for identity of putative target molecules). For FURIN we used both "general PC" network, which is based on the experimental crystal structures in Swiss-Prot protein database and experiment-based "FURIN specific" target prediction network; both analyses showed strong enrichment of putative targets in highly positively correlating genes. Interestingly, PCSK7, the only family member with no reported knock-out mouse phenotype or specific target genes [19,20], showed no significant difference in the number of putative target genes in top 5% versus bottom 5% correlates (p = 0.713). This finding may either further suggest redundancy for PCSK7 function in biology or be a sign of more limited number of target molecules. PCSK5 in part showed an outstanding number of putative target molecules amongst the negatively correlating genes. This is an intriguing phenomenon and may offer insights into the biological characteristics of this enzyme. For example, one could envision that the PCSK5 target mRNA translation may become repressed if mature protein is expressed in abundance. This could lead to lack of target enrichment in the positively correlating gene pool. We tested this hypothesis by exploring how an experimentally identified and bottom 5% target protein BACE1 [21] affects the PCSK5 expression in 293e cells. In these experiments transient overexpression of BACE1 did not cause any alterations in the PCSK mRNA expression levels (data not shown). This negative result could, in theory, imply that the target induced PCSK5 repression is either tissue or target dependent, or that PCSK5 downregulation requires a sustained expression of its target proteins.

**Table 1:** Basic information on PCSK genes and whole genome expression correlations

				Top 5% (n = 861/867)		Bottom 5% (n = 861/867)		
gene	id	# correlating genes in GeneSapiens (5%)	# of common samples with other genes	# genes with furin/general PC cleavage site and signal peptide	max/min correlation	# genes with furin/general PC cleavage site and signal peptide	max/min correlation	p value*
PCSK1	ensg00000175426	17330 (867)	445-1698	160	0.810/0.450	102	-0.246/-0.479	1.24E-4
PCSK2	ensg00000125851	17330 (867)	445-1698	170	0.871/0.541	96	-0.334/-0.637	5.10E-7
FURIN	ensg00000140564	17330 (867)	445-1698	161	0.652/0.332	83	-0.253/-0.512	4.21E-9/2.10E-7
PCSK4	ensg00000115257	17215 (861)	497-668	104	0.592/0.300	67	-0.236/-0.480	2.25E-4
PCSK5	ensg00000099139	17330 (867)	445-1698	116	0.636/0.232	158	-0.245/-0.493	4.45E-3
PCSK6	ensg00000140479	17330 (867)	445-1698	165	0.833/0.302	60	-0.277/-0.526	< 1E-10
PCSK7	ensg00000160613	17330 (867)	445-1698	111	0.571/0.312	109	-0.236/-0.481	0.71

Genes with PCSK cleavage site and signal peptide accumulate in top 5% correlating genes

* Chi-square test for top 5% vs bottom 5% correlating genes/fraction of genes with PCSK detection site and signal peptide from all 5% genes.

PCSK enzymes have displayed a significant level of redundancy between family members *in vitro *[4]. However, the phenotypes of PCSK knockout animals and patients with either gain or loss-of-function mutations in PCSK genes argue for target specificity and need for identification of specific enzyme-substrate pairs [22,23]. To gain insights into the PCSK substrate specificity we first analyzed the degree of shared putative target proteins identified in expression-correlation analysis. Table 2 lists the fractions of common putative targets for each PCSK enzyme, and extensive differences in sharing the co-expressed targets can be observed. For example, PCSK1 and PCSK2 form a unique convertase pair because they share a vast majority (75% and 80%) of their putative target molecules. Strikingly, PCSK5, the enzyme that prefers negative rather than positive expression correlation with its putative targets shares few genes with other PCSK enzymes. This finding underscores the dissimilar behavior of this enzyme in these expression correlation analyses.

**Table 2:** Fraction of putative PCSK targets found within another PCSK's putative targets

	PCSK1	PCSK2	FURIN, f	FURIN, gpc	PCSK4	PCSK5	PCSK6	PCSK7	n
PCSK1	**1**	0.800	0	0	0.006	0	0.163	0	160
PCSK2	0.753	**1**	0	0	0	0	0.276	0	170
FURIN, f	0	0	**1**	0.846	0.115	0	0.026	0.077	78
FURIN, gpc	0	0	0.410	**1**	0.130	0.006	0.019	0.087	161
PCSK4	0.010	0	0.087	0.202	**1**	0	0.106	0.010	104
PCSK5	0	0	0	0.009	0	**1**	0	0.095	116
PCSK6	0.158	0.285	0.012	0.018	0.067	0	**1**	0	165
PCSK7	0	0	0.054	0.126	0.009	0.099	0	**1**	111

f = furin-specific cleavage site

gpc = general PCSK cleavage site

Intriguingly, the previously published sequence-based PCSK comparisons resulted in nearly identical order of similarity as did our shared putative target analysis presented in Table 2[6]. The only exception was PCSK7, which is the structurally least similar enzyme with FURIN. In our analyses it has the second highest number of identical putative targets with FURIN. The substrate sharing between these two enzymes is supported by previous experimental data and a likely explanation for the observed biological redundancy of PCSK7 [24-26].

To further dissect the specificity-redundancy issue we classified the identified target molecules according to a calculated 'uniqueness value' (Additional File 5). First, the protease targets were sorted in descending order based on the correlation values with a specific PCSK gene and ordinals were recorded. Then, the same was done ascending, one by one, for all the other PCSK genes. Finally, the ordinal numbers for each of the correlating putative targets were summed up. Consequently, lower value of the summed ordinals predicts more unique PCSK - target pair. In other words, this 'uniqueness value' assorts the likelihood by which a PCSK enzyme is coordinately and specifically expressed with a target molecule and can therefore offer insights into the biological function and degree of substrate redundancy of these enzymes.

In addition to the direct modulation by transcription activators and repressors expression of a gene can also be dictated at epigenetic level. Clustering of the PCSK target genes in chromosomes might thus imply a coordinated, genome-structure manner of regulation. To test whether such clusters exist we performed a clustering analysis of the putative PCSK target genes. Intriguingly, marked differences could be observed; the putative targets for the PCSK1 and PCSK7 form several chromosomal clusters (six clusters for PCSK1, five clusters for PCSK7), whereas for example there was no chromosomal clustering of the PCSK6 target genes (Additional File 6). This could suggest that some of the PCSK enzymes regulate the expression of their targets by participating in the epigenetic modulation while others prefer a direct transcription factor based induction. Obviously, experimental evidence is required to test this hypothesis.

### Exploring the putative substrates beyond the PCSK consensus sequence

**Figure 4:**
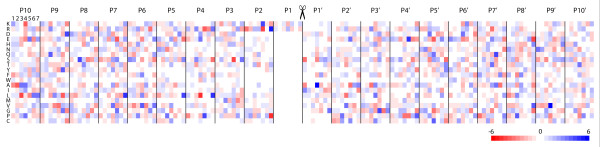
**Amino acid occurrence of putative target molecules for different PCSK enzymes**. Twenty residues (P10 - P10') around the PCSK cleavage sites of putative target molecules have been plotted for each PCSK enzymes (PCSK1-PCSK7, columns 1 - 7, respectively, in each position). Rows correspond to different amino acids. Blue color indicates increased occurrence of a particular amino acid residue type in certain position of the putative substrate when all PCSKs are considered, whereas red colors mean low occurrence of a specific amino acid. White indicates an average occurrence frequency of a specific amino acid. The increase or decrease in occurrence is shown as a scale of percentages and a combined data containing all PCSKs has been used as a comparison point. The scaling (-6 percentage to +6 percentage) is shown as a color gradient below the figure. The potentially scissile bond P1-P1' is marked with scissors.

The minimal PCSK target sequence contains basic amino acids arginine and lysine, which are critical for the substrate to bind into the negatively charged enzymatically active cleft in the PCSK enzymes. The flanking regions around the minimal consensus sequence in the PCSK targets are less well examined. The amino acid compositions around the recognition sequences were first visualized using MultiDisp server (http://bioinf.uta.fi/cgi-bin/MultiDisp.cgi, Figure 4, Additional File 7). We focused on twenty amino acid residues close to the putative PCSK cleavage site (upstream P1-P10, downstream P1'-P10') to see if any common patterns can be identified for a specific PCSK enzyme. As expected, the predicted target sequences for all PCSKs clearly favored either arginine or lysine at the P1 and P2, and P4. When the targets of a PCSK were explored individually many amino acid residues with only subtle preferences could be seen at other positions. To perform a systematic analysis of the preferred/unfavored residues we then calculated the frequency difference from a mean for each amino acid at sites P10-P10' using the amino acid frequency tables from MultiDisp (Figure 4).

Our analysis showed minor differences in the target alignment segment, where a consensus sequence of Rx[KR]R is favored. Putative targets of PCSK7 have a slight enrichment of arginine in the P2. This might be explained by the PCSK7 β8-β9 loop having a glycine aligning the P2 site instead of the glutamate in PCSK1/4/5/6 or the phenylalanine in PCSK2. Small glycine residue might allow more space for the bulky arginine side chain. In addition, PCSK5, which showed putative target enrichment in the bottom 5% correlating genes, seem to have a stronger preference for lysine at this site when compared to other PCSKs. Interestingly, when the inversely expressed putative targets from the negatively correlating genes for PCSK5 were analyzed we found a strong preference for arginine at P2 site (Additional File 8). PCSK4 appears to allow negative charged residues at the P4 site, which cannot be explained in the electrostatics of the PCSK4 residues interacting with the P4. Just outside the substrate alignment segment, at the P5, acidic residues are preferred in all groups of putative targets, except for PCSK7. It is also noteworthy that the putative targets of PCSK7 have leucines enriched at sites P4, P5 and P7. The hydrophobic nature of leucine would suggest higher extent of hydrophobic interactions between PCSK7 and target sequences in contrast to other PCSKs.

We did not find strong patterns of favored amino acids for the sites P1'-P10' that would explain substrate specificity of the PCSKs. However, position P5' was found to be quite variable and to slightly favor acidic residues, with the exception of PCSK4. Glutamine and alanine were found slightly enriched in the P7' position in PCSK5. These enriched residue types at certain positions could hint at sequence-specific substrate recognition, but additional studies are needed to prove their contribution to the biological function.

### Biochemical identification of PAPPA1 and ADAMTS6 as novel FURIN substrates

As previously pointed out the coordinated expression of PCSK and its substrates is supported by scattered experimental evidence. In addition, a previous report has convincingly shown the validity of ProP prediction in selecting PCSK targets in FURIN deficient mouse liver *in vivo *[27]. These data show that ProP can predict the physiological PCSK processed proteins fairly accurately, but also that the mainly co-expression experiment data based FURIN prediction algorithm cannot discriminate the physiological FURIN specific target molecules from general PCSK targets. Our genome-wide analysis identified several previously published targets for the PCSK enzymes, for example, the list of putative FURIN target molecules includes matrix metalloproteinases (MMP11), growth factors (PDGFB), and cytokines (BMP1) that have been previously been identified as PCSK targets [25,28,29]. Notably, the list lacks a physiological FURIN target TGFβ1, which shows a highly coordinated expression with FURIN in the tissues like blood myeloid and lymphoid cells (correlation values in GeneSapiens analysis of r = 0.743 and 0.596, respectively) [30,31]. When exploring the putative target list of PCSK1 and PCSK2 enzymes, which have a more restricted expression pattern than FURIN, we noted that several biological targets, such as proSAAS (ENSG00000102109) and prosomatostatin (ENSG00000157005), can be identified. Therefore, our approach seems to work particularly efficiently when both PCSK and its substrate have generally restricted expression. In contrast, genome-wide approach may fall short in identifying tissue specific substrates for widely expressed PCSK enzymes.

**Figure 5:**
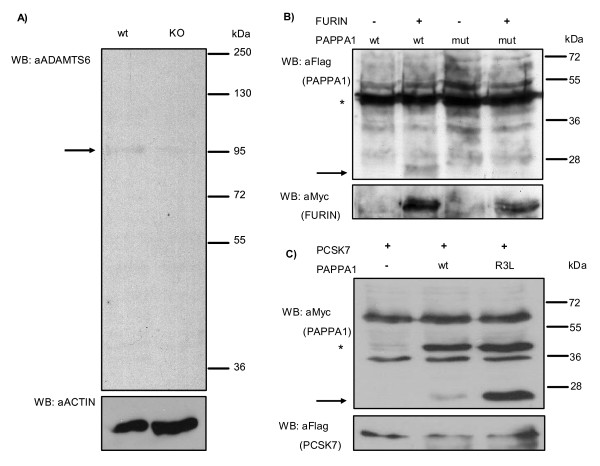
**Proteolytic processing of ADAMTS6 and PAPPA1**. A) Aliquots of CD4+ T cell lysates from wild-type and FURIN knock-out mice were run on SDS-PAGE and ADAMTS6 and ACTIN were detected by Western blot. B and C) 293e cells were transfected with FURIN-myc-his or PCSK7-flag together with wild-type, PCSK cleavage site mutated flag-PAPPA1-myc (mut) or PCSK7-favored leucines harboring flag-PAPPA1-myc (R3L) constructs, and detected anti-flag or anti-Myc antibodies as indicated. Unprocessed PAPPA1 is indicated with an asterisk and processed with an arrow. All experiments were repeated at least twice with similar results.

We validated our approach biochemically by identifying novel PCSK target genes on our lists. We chose to look for novel FURIN processed molecules from both general PCSK and "FURIN specific" target lists. We selected two highly correlating genes with a human-mouse conserved PCSK target sequence ADAMTS6 (ENSG00000049192, correlation value 0.34) and PAPPA1 (ENSG00000182752, correlation value 0.41), which have not previously been experimentally shown to be processed by FURIN. Further, we have previously shown that in FURIN knock-out T cells ADAMTS6 is down-regulated, which is in keeping with our theory that the PCSK target molecules are coordinately regulated with their converting enzyme [31]. In Figure 5A, we blotted for endogenous ADAMTS6 molecule in wild-type and FURIN knock-out CD4+ T cells with an antibody that specifically recognizes the catalytic domain of ADAMTS6. Our results show that the 97 kDA band that represents the processed ADAMTS6 molecule is clearly reduced in FURIN knock-out samples (Figure 5A). Furthermore, in Figure 5B we show that PAPPA1, which contains putative PCSK target sites in its N-terminus is only processed if FURIN cDNA is co-expressed with the N-terminal part of PAPPA1. The mutation of the putative FURIN target sequences in PAPPA1 makes it resistant for FURIN's proteolytic activity. These results unequivocally show that expression correlation analysis combined with PCSK target site prediction can be used to identify novel target molecules for the PCSK enzymes.

Finally, to test whether the enrichment of certain amino acids around the cleavage site would change the target preferences for PCSKs we mutated arginines at P4, P5 and P7 positions of PAPPA1 into PCSK7-favored leucines (Arg24/26/27Leu, Figure 5C). In these overexpression experiments wild-type N-terminus of PAPPA1 was processed by PCKS7 to comparable extent with FURIN, a finding that underscores again the limitations of this approach in identifying specific substrates. However, PAPPA1 construct that harbors the favored leucines (R3L) was much more potently processed by PCSK7 when compared with wild-type PAPPA1. This result confirms that the abovementioned exploration of the cleavage site flanking sequences may indeed give insights to the substrate preferences of a PCSK. However, true *in vivo *identification of such critical amino acids would require an analysis using for example knock-in mice or mutant patient cell lines.

## Conclusions

The biological significance of the PCSK enzymes is indisputable and interfering with their activity holds promise for future therapies in diseases ranging from atherosclerosis to cancers and infections. Therefore, understanding the determinants of the substrate specificity of PCSKs enzymes is of utmost importance. Traditional biochemical experiments where a PCSK is co-expressed *in vitro *with its putative target molecule have certainly improved our understanding on the PCSK function, but can also lead to misinterpretation on the biological role of a PCSK. Our data presented herein shows that most PCSK enzymes are coordinately expressed with their putative target proteins. Exploring this phenomenon can complement the *in vitro *experiments and can also offer insights into the true biological function of these enzymes in health and disease.

## Methods

### Expression data and correlation

Expression data and correlation values used in the analyses were obtained from the GeneSapiens database, described elsewhere (http://www.genesapiens.org, [16]). Briefly, expression correlations of all the PCSK genes with all the other genes in the human genome (n = 17330) were analyzed in large number (n = 1869) of samples. Only healthy samples over the whole spectrum of human tissues (altogether 43 distinct tissue types) were used. Expression correlation values for all the PCSK genes are provided in the Additional File 2.

Data analysis for correlation was done with R. The correlation metric used was Pearson correlation coefficient. The coefficient was calculated using all samples that had expression values for both genes in the analysis, with a minimum requirement of 10 common samples. Additional File 2 provides two correlation coefficients, non-log and log. Log values are correlations for gene expression patterns that have undergone log2 transformation, and non-log values are determined straight from the measured expression values. The non-log values were used in this analysis.

Genes with strong positive correlation in expression with PCSK (top 5%) and strong negative correlation in expression with PCSK (bottom 5%) were extracted from the expression correlation lists for all the PCSK genes (Figure 2, Additional File 2). This resulted 867 (= 17330 × 0.05; 861 for PCSK4 (= 17215 × 0.05)) genes correlating strongly (positively or negatively) with PCSK gene expression. Secondly, as proprotein convertases and their substrates were assumed to be present in the same cell compartments, the presence of a protein secretion signal peptide in correlates (minimum 11 amino acid long) was required. Thirdly, proprotein cleavage sites outside of the signal peptide were predicted with the ProP server (http://www.cbs.dtu.dk/services/ProP/) using the general PC prediction [17]. Narrowing down the top 5% and bottom 5% gene lists with these inclusion criteria of signal peptide and proprotein cleavage site yielded some 67 to 170 highly (positively or negatively) correlating genes per PSCK gene (Table 1, Additional File 2). Highly positively expression correlated genes that fulfilled the inclusion criteria were named as 'putative PCSK targets'.

### PCSK protein modeling

To investigate the interactions of the putative targets with the catalytic cleft, we modelled the different PCSKs with the program Modeller 9v7 using the crystallographic structure of FURIN as template (PDB ID: 1P8J[11]). The models were in good agreement with those previously described [6], kindly provided by Stefan Henrich. The conservation of the substrate-recognition site was evaluated by comparing the sequences of PCs from 26 species, covering altogether 152 amino acid sequences. The sequence alignments were generated using ClustalW2 [32]. The conserved (>95%) residues were visualized into the crystal structure of FURIN using program PyMOL 1.4.

### Substrate recognition sequence

The top-correlates containing signal peptide sequences were subjected to computational screening of PCSK cleavage sites. The cleavage sites found in the putative target proteins were analyzed using the MultiDisp tool that plots the amino acid frequencies (Figure 4, Additional File 7, Additional File 8). The frequencies were also compared to the mean of all putative cleavage sites (Figure 4).

### Chromosomal clustering of targets identified

A clustering analyses for the putative targets identified was performed using CROC software (http://metagenomics.uv.es/CROC, [33]). A sliding window of 20 genes, minimum number of three genes expected per cluster and Benjamini&Hochberg correction for multiple testing were applied for statistical analysis.

### In vitro identification of the FURIN target molecules

DNA sequences encoding the N-terminal amino acids Arg2 to Ala200 of human PAPPA1 with N-terminal FLAG and hemagglutin (HA) tags from GeneArt (http://www.geneart.com) and full-length human FURIN from ATCC were both cloned into pcDNA3.1-myc-his expression vector. PCSK7-flag plasmid was a kind gift from Prof. John Creemers (Center for Human Genetics, K. U. Leuven, Leuven, Belgium). PAPPA1R3L construct (Arg24/26/27Leu) was cloned using QuikChange Mutagenesis Kit (Stratagene). HEK 293e cells were cultured in Dulbecco's modified Eagle's medium (DMEM) supplemented by 10% fetal calf serum (FCS) and 1% penicillin/streptomycin. Cells were transiently transfected with FURIN or PCSK7 and PAPPA1 constructs using TurboFect transfection reagent (Fermentas) according to the manufacturer's instructions. 48 hours post-transfection, cells were washed once with cold phosphate-buffered saline (PBS), and lysed into Triton-X lysis buffer. CD4+ cells from spleen and lymph nodes of wild-type and T-cell specific furin knock-out mice [31] were purified by positive selection using magnetic beads (Miltenyi Biotech) and lysed into Triton-X lysis buffer. Aliquots of cell lysates were run on 12% SDS-PAGE gels. Western blotting was performed using ADAMTS6 (ab50647, Abcam), actin (Millipore), myc and FLAG (Sigma) antibodies.

## Authors' contributions

HT and MP formulated the original research idea and wrote the manuscript. HT and SK performed analyses for correlated genes. SK, TK and VH performed structural analyses and contributed to the manuscript preparation. KP and MP biochemically identified novel FURIN substrates. KO did the correlation analysis for all the PCSK genes.

All authors read, commented and approved the final manuscript.

## Supplementary Material

Additional file 1 **PCSK sequences used for Figure 1**. Altogether 152 PCSK sequences were used. Click here for file

Additional file 2 **Whole genome expression correlations for PCSK1-7 genes**. Non-logarithmic and logarithmic correlations as well as p values and common data points are listed. Genes in blue are bottom 5% genes that fulfill the inclusion criteria of signal peptide and PCSK recognition site. Red ones are the corresponding genes for top 5%. Click here for file

Additional file 3 **Mutual expression correlation between the PCSK gene pairs over the whole spectrum of healthy tissues**. Click here for file

Additional file 4 **Mutual expression correlation between the PCSK gene pairs specified in different anatomical structures**. Click here for file

Additional file 5 **Uniqueness expression correlation values of putative PCSK targets**. The uniqueness values for putative targets were counted as follows: First, the correlating genes were sorted descending by correlation values with specific PCSK gene and ordinals were recorded. Then, the same was done ascending, one by one, for all the other PCSK genes. Finally, the ordinal numbers for each of the correlating putative targets were summed up. The lower the summed value the more unique the expression correlation is for the specific PCSK gene. Click here for file

Additional file 6 **Chromosomal clustering of putative PCSK targets**. Click here for file

Additional file 7 **MultiDisp figures of potential PCSK target sequences**. The sequences were predicted from the group of top-correlated gene translations using the general PC prediction and signal peptide prediction methods on the ProP 1.0 server (http://www.cbs.dtu.dk/services/ProP/). Potential target peptides from signal peptide-containing sequences were restricted to ten residues upstream (P10-P1) and downstream (P1'-P10') of the predicted cleavage site. Amino acid compositions at each site in the groups of potential targets for each PCSK were plotted using MultiDisp (http://bioinf.uta.fi/cgi-bin/MultiDisp.cgi) that scales the character heights based on amino acid frequency. Scissors and dotted line mark the predicted cleavage site. Click here for file

Additional file 8 **Amino acid occurrence of putative target molecules for PCSK5**. Twenty residues (P10 - P10', marked with numbers 10 - 10') around the PCSK cleavage sites of highly correlating genes have been plotted for PCSK5. Top (t) and bottom (b) groups are shown for each amino acid type. Blue color indicates increased occurrence of a particular amino acid residue type in certain position of the putative substrate when all PCSKs are considered, whereas red colors mean low occurrence of a specific amino acid. White indicates an average occurrence frequency of a specific amino acid. The increase or decrease in occurrence is shown as a scale of percentages and a combined data containing all PCSKs has been used as a comparison point. The scaling (-6 percentage to +6 percentage) is shown as a color gradient below the figure. The potentially scissile bond P1-P1' is marked with scissors and dashed line. Click here for file
