# Supplementary material for: Identification of proprotein convertase substrates using genome-wide expression correlation analysis
Source: BMC Genomics. 2011 Dec 20;12:618. doi: 10.1186/1471-2164-12-618 (PMC3258279; doi:10.1186/1471-2164-12-618)
Supplement: Additional file 3 — Mutual expression correlation between the PCSK gene pairs over the whole spectrum of healthy tissues. [file 1471-2164-12-618-S3.PDF]

Supplementary Data 3. Mutual non-log expression correlation (ranking among all the 17330 (17215 for PCSK4) genes) for PCSK genes.

gene	PCSK1	PCSK2	FURIN	PCSK4	PCSK5	PCSK6	PCSK7
**PCSK1**	1 (1.)	0.6766 (88.)	-0.1694 (14114.)	-0.0738 (8603.)	-0.1725 (14266.)	0.0666 (4183.)	-0.1518 (13249.)
**PCSK2**	0.6766 (342.)	1 (1.)	-0.1795 (12573.)	-0.073 (8139.)	-0.2313 (14401.)	0.1014 (4078.)	-0.1474 (11224.)
**FURIN**	-0.1694 (14965.)	-0.1795 (15181.)	1 (1.)	0.1534 (4494.)	-0.0827 (12826.)	0.0791 (7015.)	0.1815 (3726.)
**PCSK4**	-0.0738 (12409.)	-0.073 (12375.)	0.1534 (3698.)	1 (1.)	-0.2112 (16045.)	0.1459 (3929.)	0.0029 (9339.)
**PCSK5**	-0.1725 (15214.)	-0.2313 (16283.)	-0.0827 (12545.)	-0.2112 (15947.)	1 (1.)	-0.187 (15499.)	0.0734 (5101.)
**PCSK6**	0.0666 (5317.)	0.1014 (4217.)	0.0791 (4923.)	0.1459 (3044.)	-0.187 (14649.)	1 (1.)	-0.0603 (10198.)
**PCSK7**	-0.1518 (14668.)	-0.1474 (14539.)	0.1815 (2853.)	0.0029 (9096.)	0.0734 (6174.)	-0.0603 (11607.)	1 (1.)

negative correlations in light shaded background

self correlations in dark shaded background
