# Supplementary material for: Identification of proprotein convertase substrates using genome-wide expression correlation analysis
Source: BMC Genomics. 2011 Dec 20;12:618. doi: 10.1186/1471-2164-12-618 (PMC3258279; doi:10.1186/1471-2164-12-618)
Supplement: Additional file 4 — Mutual expression correlation between the PCSK gene pairs specified in different anatomical structures. [file 1471-2164-12-618-S4.PDF]

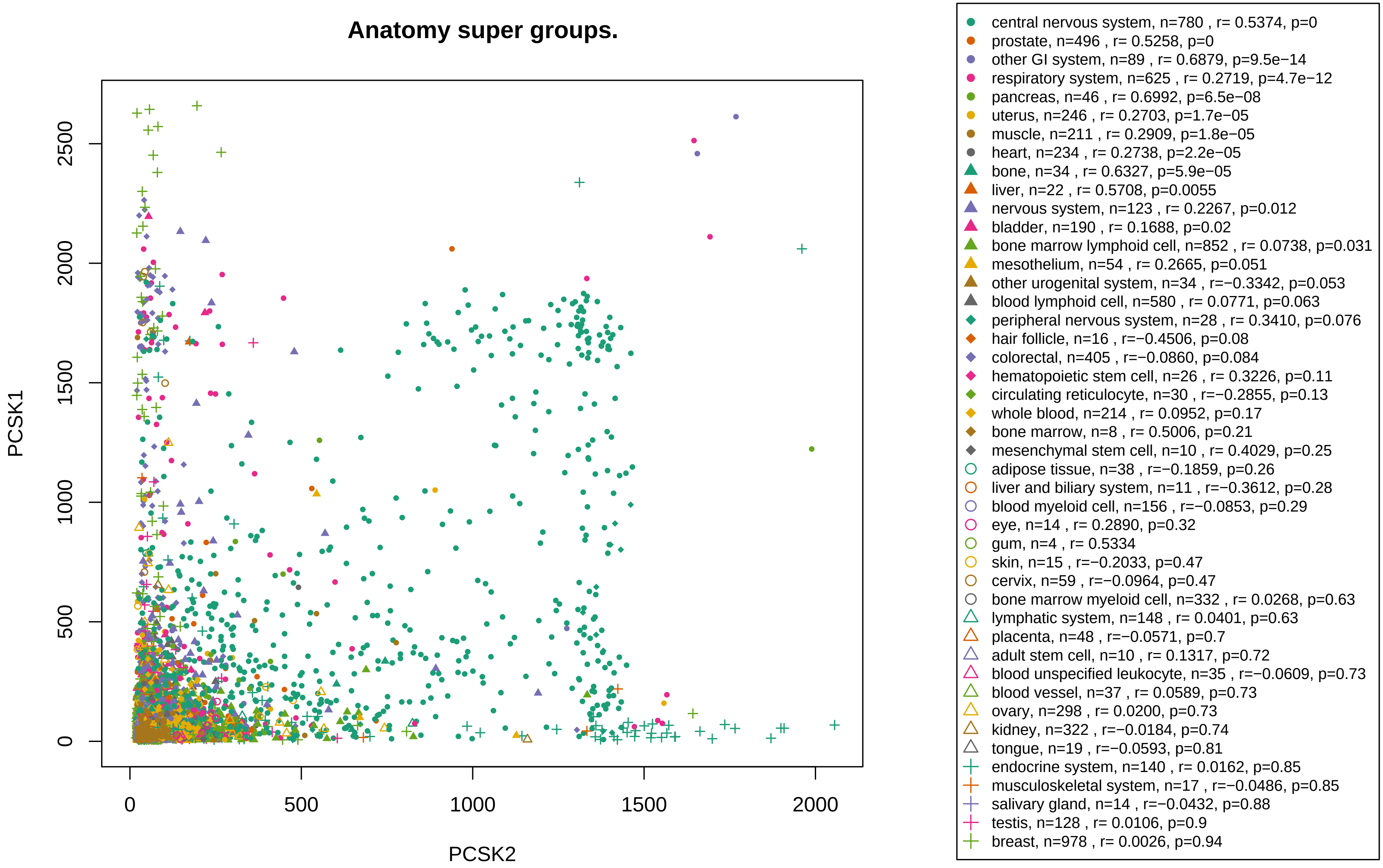
Anatomy super groups.
PCSK1
PCSK2
0
500
1000
1500
2000
2500
central nervous system, n=780 , r= 0.5374, p=0
prostate, n=496 , r= 0.5258, p=0
other GI system, n=89 , r= 0.6879, p=9.5e−14
respiratory system, n=625 , r= 0.2719, p=4.7e−12
pancreas, n=46 , r= 0.6992, p=6.5e−08
uterus, n=246 , r= 0.2703, p=1.7e−05
muscle, n=211 , r= 0.2909, p=1.8e−05
heart, n=234 , r= 0.2738, p=2.2e−05
bone, n=34 , r= 0.6327, p=5.9e−05
liver, n=22 , r= 0.5708, p=0.0055
nervous system, n=123 , r= 0.2267, p=0.012
bladder, n=190 , r= 0.1688, p=0.02
bone marrow lymphoid cell, n=852 , r= 0.0738, p=0.031
mesothelium, n=54 , r= 0.2665, p=0.051
other urogenital system, n=34 , r=−0.3342, p=0.053
blood lymphoid cell, n=580 , r= 0.0771, p=0.063
peripheral nervous system, n=28 , r= 0.3410, p=0.076
hair follicle, n=16 , r=−0.4506, p=0.08
colorectal, n=405 , r=−0.0860, p=0.084
hematopoietic stem cell, n=26 , r= 0.3226, p=0.11
circulating reticulocyte, n=30 , r=−0.2855, p=0.13
whole blood, n=214 , r= 0.0952, p=0.17
bone marrow, n=8 , r= 0.5006, p=0.21
mesenchymal stem cell, n=10 , r= 0.4029, p=0.25
adipose tissue, n=38 , r=−0.1859, p=0.26
liver and biliary system, n=11 , r=−0.3612, p=0.28
blood myeloid cell, n=156 , r=−0.0853, p=0.29
eye, n=14 , r= 0.2890, p=0.32
gum, n=4 , r= 0.5334
skin, n=15 , r=−0.2033, p=0.47
cervix, n=59 , r=−0.0964, p=0.47
bone marrow myeloid cell, n=332 , r= 0.0268, p=0.63
lymphatic system, n=148 , r= 0.0401, p=0.63
placenta, n=48 , r=−0.0571, p=0.7
adult stem cell, n=10 , r= 0.1317, p=0.72
blood unspecified leukocyte, n=35 , r=−0.0609, p=0.73
blood vessel, n=37 , r= 0.0589, p=0.73
ovary, n=298 , r= 0.0200, p=0.73
kidney, n=322 , r=−0.0184, p=0.74
tongue, n=19 , r=−0.0593, p=0.81
endocrine system, n=140 , r= 0.0162, p=0.85
musculoskeletal system, n=17 , r=−0.0486, p=0.85
salivary gland, n=14 , r=−0.0432, p=0.88
testis, n=128 , r= 0.0106, p=0.9
breast, n=978 , r= 0.0026, p=0.94

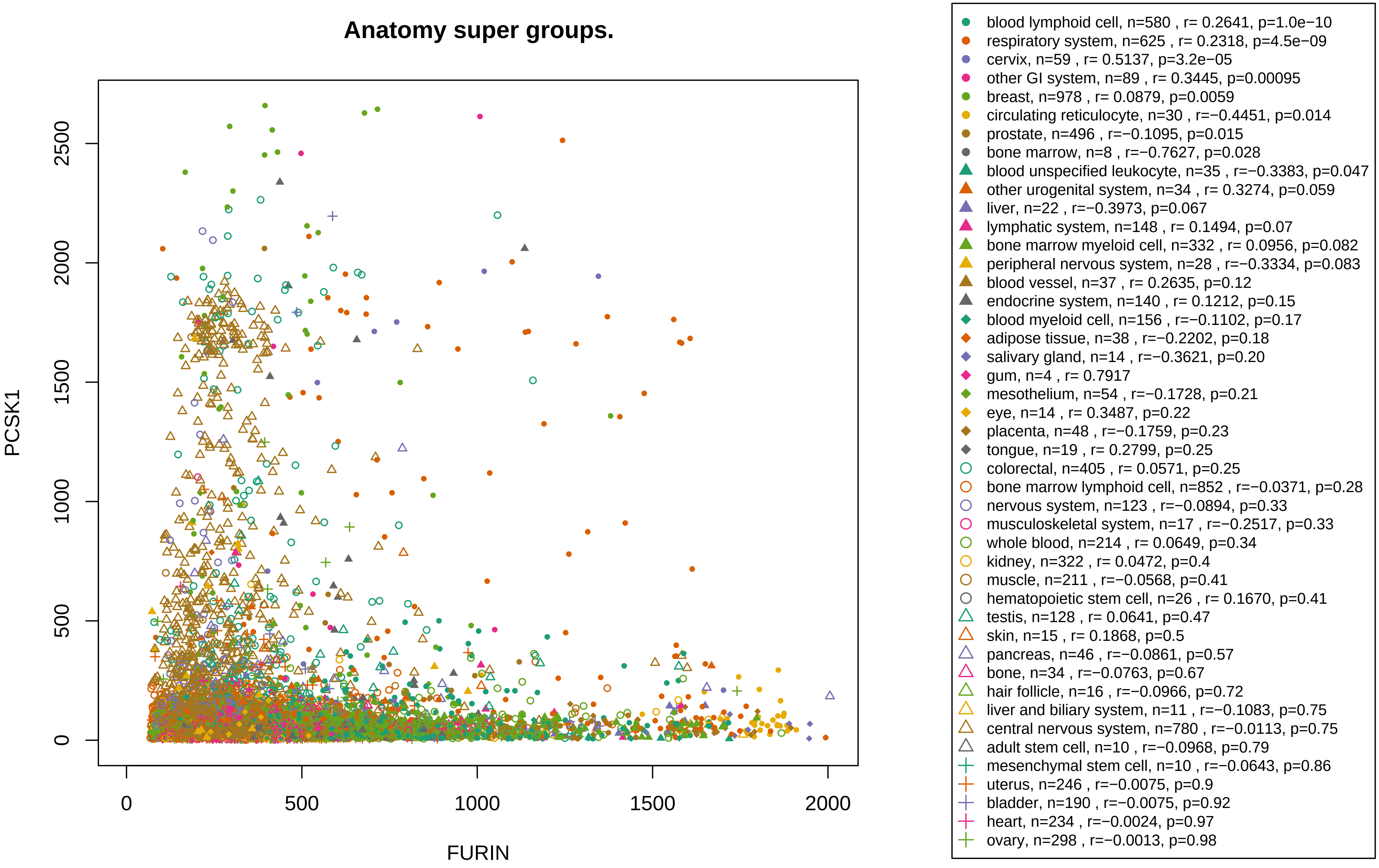
Anatomy super groups.
PCSK1
FURIN
0
500
1000
1500
2000
2500
blood lymphoid cell, n=580 , r= 0.2641, p=1.0e−10
respiratory system, n=625 , r= 0.2318, p=4.5e−09
cervix, n=59 , r= 0.5137, p=3.2e−05
other GI system, n=89 , r= 0.3445, p=0.00095
breast, n=978 , r= 0.0879, p=0.0059
circulating reticulocyte, n=30 , r=−0.4451, p=0.014
prostate, n=496 , r=−0.1095, p=0.015
bone marrow, n=8 , r=−0.7627, p=0.028
blood unspecified leukocyte, n=35 , r=−0.3383, p=0.047
other urogenital system, n=34 , r= 0.3274, p=0.059
liver, n=22 , r=−0.3973, p=0.067
lymphatic system, n=148 , r= 0.1494, p=0.07
bone marrow myeloid cell, n=332 , r= 0.0956, p=0.082
peripheral nervous system, n=28 , r=−0.3334, p=0.083
blood vessel, n=37 , r= 0.2635, p=0.12
endocrine system, n=140 , r= 0.1212, p=0.15
blood myeloid cell, n=156 , r=−0.1102, p=0.17
adipose tissue, n=38 , r=−0.2202, p=0.18
salivary gland, n=14 , r=−0.3621, p=0.20
gum, n=4 , r= 0.7917
mesothelium, n=54 , r=−0.1728, p=0.21
eye, n=14 , r= 0.3487, p=0.22
placenta, n=48 , r=−0.1759, p=0.23
tongue, n=19 , r= 0.2799, p=0.25
colorectal, n=405 , r= 0.0571, p=0.25
bone marrow lymphoid cell, n=852 , r=−0.0371, p=0.28
nervous system, n=123 , r=−0.0894, p=0.33
musculoskeletal system, n=17 , r=−0.2517, p=0.33
whole blood, n=214 , r= 0.0649, p=0.34
kidney, n=322 , r= 0.0472, p=0.4
muscle, n=211 , r=−0.0568, p=0.41
hematopoietic stem cell, n=26 , r= 0.1670, p=0.41
testis, n=128 , r= 0.0641, p=0.47
skin, n=15 , r= 0.1868, p=0.5
pancreas, n=46 , r=−0.0861, p=0.57
bone, n=34 , r=−0.0763, p=0.67
hair follicle, n=16 , r=−0.0966, p=0.72
liver and biliary system, n=11 , r=−0.1083, p=0.75
central nervous system, n=780 , r=−0.0113, p=0.75
adult stem cell, n=10 , r=−0.0968, p=0.79
mesenchymal stem cell, n=10 , r=−0.0643, p=0.86
uterus, n=246 , r=−0.0075, p=0.9
bladder, n=190 , r=−0.0075, p=0.92
heart, n=234 , r=−0.0024, p=0.97
ovary, n=298 , r=−0.0013, p=0.98

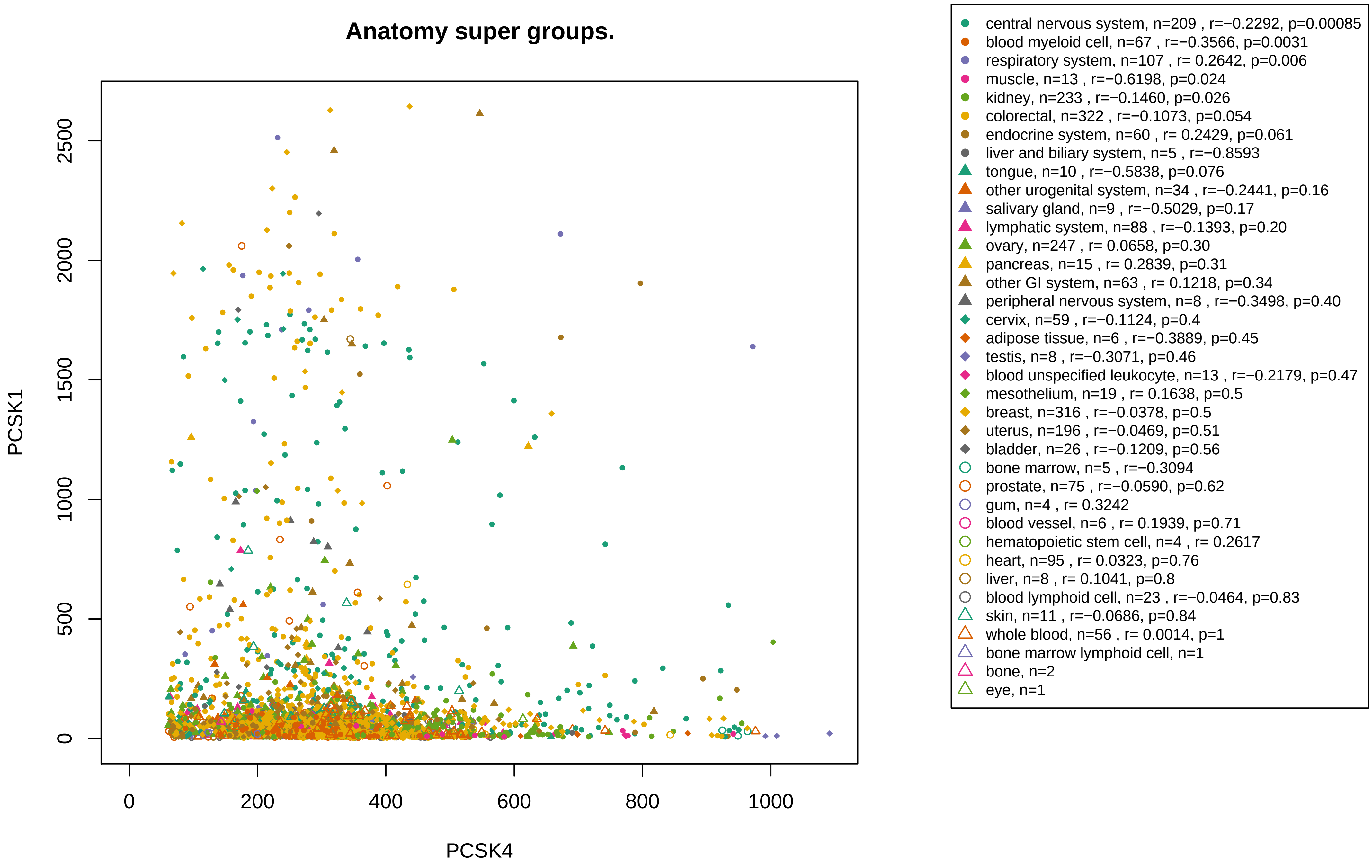
Anatomy super groups.
PCSK1
PCSK4
0
500
1000
1500
2000
2500
0
200
400
600
800
1000
central nervous system, n=209 , r=−0.2292, p=0.00085
blood myeloid cell, n=67 , r=−0.3566, p=0.0031
respiratory system, n=107 , r= 0.2642, p=0.006
muscle, n=13 , r=−0.6198, p=0.024
kidney, n=233 , r=−0.1460, p=0.026
colorectal, n=322 , r=−0.1073, p=0.054
endocrine system, n=60 , r= 0.2429, p=0.061
liver and biliary system, n=5 , r=−0.8593
tongue, n=10 , r=−0.5838, p=0.076
other urogenital system, n=34 , r=−0.2441, p=0.16
salivary gland, n=9 , r=−0.5029, p=0.17
lymphatic system, n=88 , r=−0.1393, p=0.20
ovary, n=247 , r= 0.0658, p=0.30
pancreas, n=15 , r= 0.2839, p=0.31
other GI system, n=63 , r= 0.1218, p=0.34
peripheral nervous system, n=8 , r=−0.3498, p=0.40
cervix, n=59 , r=−0.1124, p=0.4
adipose tissue, n=6 , r=−0.3889, p=0.45
testis, n=8 , r=−0.3071, p=0.46
blood unspecified leukocyte, n=13 , r=−0.2179, p=0.47
mesothelium, n=19 , r= 0.1638, p=0.5
breast, n=316 , r=−0.0378, p=0.5
uterus, n=196 , r=−0.0469, p=0.51
bladder, n=26 , r=−0.1209, p=0.56
bone marrow, n=5 , r=−0.3094
prostate, n=75 , r=−0.0590, p=0.62
gum, n=4 , r= 0.3242
blood vessel, n=6 , r= 0.1939, p=0.71
hematopoietic stem cell, n=4 , r= 0.2617
heart, n=95 , r= 0.0323, p=0.76
liver, n=8 , r= 0.1041, p=0.8
blood lymphoid cell, n=23 , r=−0.0464, p=0.83
skin, n=11 , r=−0.0686, p=0.84
whole blood, n=56 , r= 0.0014, p=1
bone marrow lymphoid cell, n=1
bone, n=2
eye, n=1

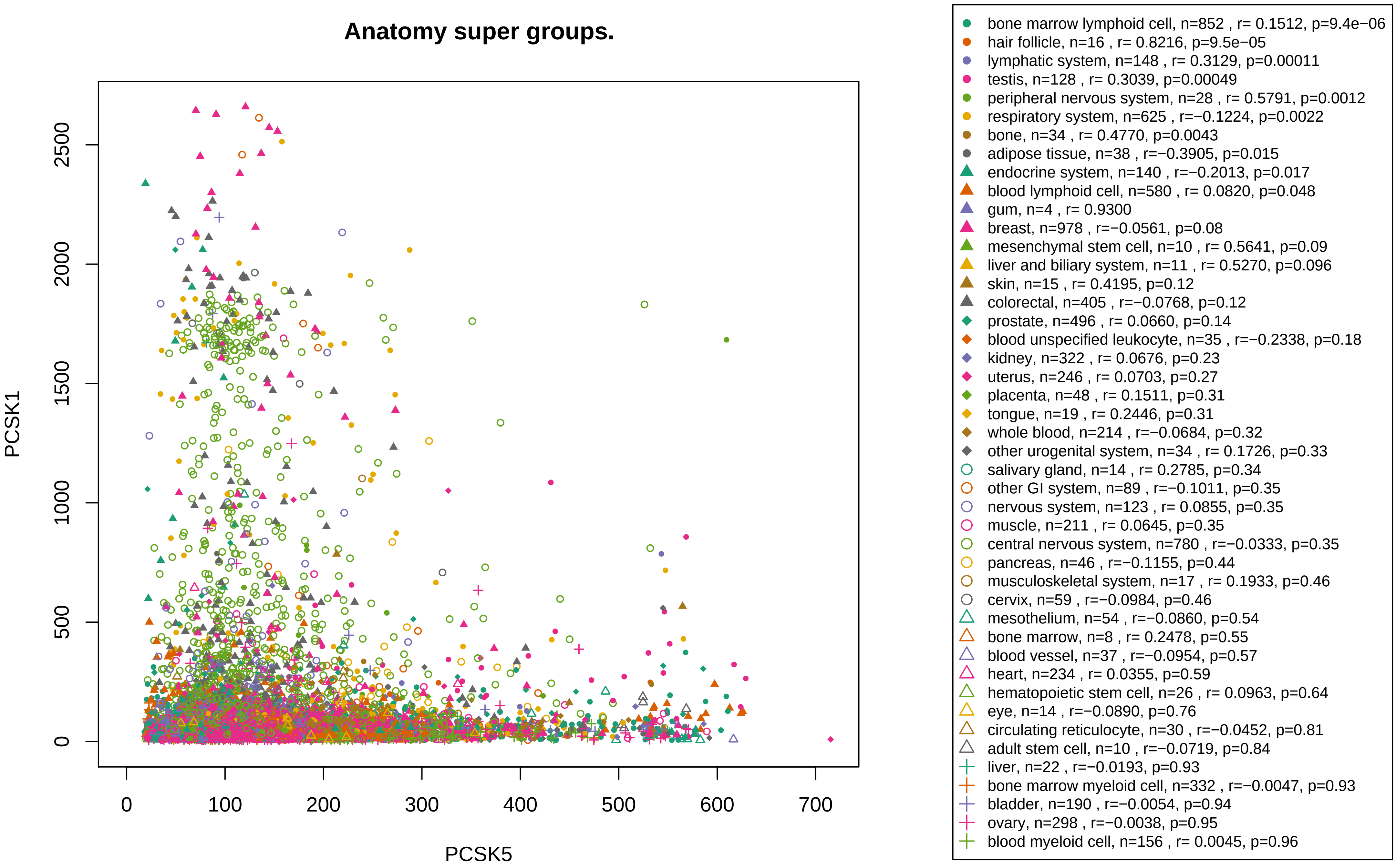
Anatomy super groups.
PCSK1
PCSK5
0
500
1000
1500
2000
2500
0
100
200
300
400
500
600
700
bone marrow lymphoid cell, n=852 , r= 0.1512, p=9.4e−06
hair follicle, n=16 , r= 0.8216, p=9.5e−05
lymphatic system, n=148 , r= 0.3129, p=0.00011
testis, n=128 , r= 0.3039, p=0.00049
peripheral nervous system, n=28 , r= 0.5791, p=0.0012
respiratory system, n=625 , r=−0.1224, p=0.0022
bone, n=34 , r= 0.4770, p=0.0043
adipose tissue, n=38 , r=−0.3905, p=0.015
endocrine system, n=140 , r=−0.2013, p=0.017
blood lymphoid cell, n=580 , r= 0.0820, p=0.048
gum, n=4 , r= 0.9300
breast, n=978 , r=−0.0561, p=0.08
mesenchymal stem cell, n=10 , r= 0.5641, p=0.09
liver and biliary system, n=11 , r= 0.5270, p=0.096
skin, n=15 , r= 0.4195, p=0.12
colorectal, n=405 , r=−0.0768, p=0.12
prostate, n=496 , r= 0.0660, p=0.14
blood unspecified leukocyte, n=35 , r=−0.2338, p=0.18
kidney, n=322 , r= 0.0676, p=0.23
uterus, n=246 , r= 0.0703, p=0.27
placenta, n=48 , r= 0.1511, p=0.31
tongue, n=19 , r= 0.2446, p=0.31
whole blood, n=214 , r=−0.0684, p=0.32
other urogenital system, n=34 , r= 0.1726, p=0.33
salivary gland, n=14 , r= 0.2785, p=0.34
other GI system, n=89 , r=−0.1011, p=0.35
nervous system, n=123 , r= 0.0855, p=0.35
muscle, n=211 , r= 0.0645, p=0.35
central nervous system, n=780 , r=−0.0333, p=0.35
pancreas, n=46 , r=−0.1155, p=0.44
musculoskeletal system, n=17 , r= 0.1933, p=0.46
cervix, n=59 , r=−0.0984, p=0.46
mesothelium, n=54 , r=−0.0860, p=0.54
bone marrow, n=8 , r= 0.2478, p=0.55
blood vessel, n=37 , r=−0.0954, p=0.57
heart, n=234 , r= 0.0355, p=0.59
hematopoietic stem cell, n=26 , r= 0.0963, p=0.64
eye, n=14 , r=−0.0890, p=0.76
circulating reticulocyte, n=30 , r=−0.0452, p=0.81
adult stem cell, n=10 , r=−0.0719, p=0.84
liver, n=22 , r=−0.0193, p=0.93
bone marrow myeloid cell, n=332 , r=−0.0047, p=0.93
bladder, n=190 , r=−0.0054, p=0.94
ovary, n=298 , r=−0.0038, p=0.95
blood myeloid cell, n=156 , r= 0.0045, p=0.96

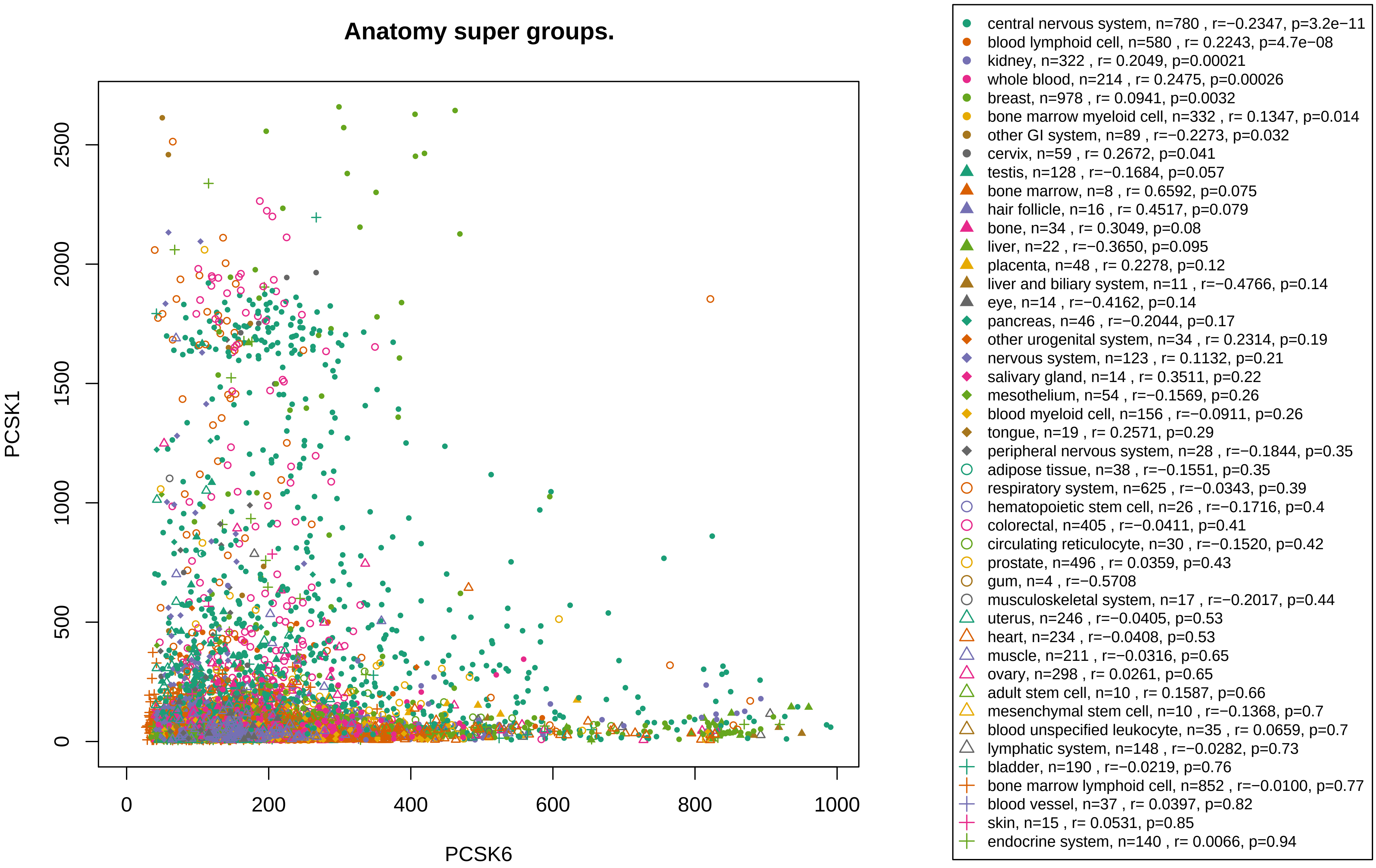
Anatomy super groups.
PCSK1
PCSK6
0
500
1000
1500
2000
2500
0
200
400
600
800
1000
central nervous system, n=780 , r=−0.2347, p=3.2e−11
blood lymphoid cell, n=580 , r= 0.2243, p=4.7e−08
kidney, n=322 , r= 0.2049, p=0.00021
whole blood, n=214 , r= 0.2475, p=0.00026
breast, n=978 , r= 0.0941, p=0.0032
bone marrow myeloid cell, n=332 , r= 0.1347, p=0.014
other GI system, n=89 , r=−0.2273, p=0.032
cervix, n=59 , r= 0.2672, p=0.041
testis, n=128 , r=−0.1684, p=0.057
bone marrow, n=8 , r= 0.6592, p=0.075
hair follicle, n=16 , r= 0.4517, p=0.079
bone, n=34 , r= 0.3049, p=0.08
liver, n=22 , r=−0.3650, p=0.095
placenta, n=48 , r= 0.2278, p=0.12
liver and biliary system, n=11 , r=−0.4766, p=0.14
eye, n=14 , r=−0.4162, p=0.14
pancreas, n=46 , r=−0.2044, p=0.17
other urogenital system, n=34 , r= 0.2314, p=0.19
nervous system, n=123 , r= 0.1132, p=0.21
salivary gland, n=14 , r= 0.3511, p=0.22
mesothelium, n=54 , r=−0.1569, p=0.26
blood myeloid cell, n=156 , r=−0.0911, p=0.26
tongue, n=19 , r= 0.2571, p=0.29
peripheral nervous system, n=28 , r=−0.1844, p=0.35
adipose tissue, n=38 , r=−0.1551, p=0.35
respiratory system, n=625 , r=−0.0343, p=0.39
hematopoietic stem cell, n=26 , r=−0.1716, p=0.4
colorectal, n=405 , r=−0.0411, p=0.41
circulating reticulocyte, n=30 , r=−0.1520, p=0.42
prostate, n=496 , r= 0.0359, p=0.43
gum, n=4 , r=−0.5708
musculoskeletal system, n=17 , r=−0.2017, p=0.44
uterus, n=246 , r=−0.0405, p=0.53
heart, n=234 , r=−0.0408, p=0.53
muscle, n=211 , r=−0.0316, p=0.65
ovary, n=298 , r= 0.0261, p=0.65
adult stem cell, n=10 , r= 0.1587, p=0.66
mesenchymal stem cell, n=10 , r=−0.1368, p=0.7
blood unspecified leukocyte, n=35 , r= 0.0659, p=0.7
lymphatic system, n=148 , r=−0.0282, p=0.73
bladder, n=190 , r=−0.0219, p=0.76
bone marrow lymphoid cell, n=852 , r=−0.0100, p=0.77
blood vessel, n=37 , r= 0.0397, p=0.82
skin, n=15 , r= 0.0531, p=0.85
endocrine system, n=140 , r= 0.0066, p=0.94

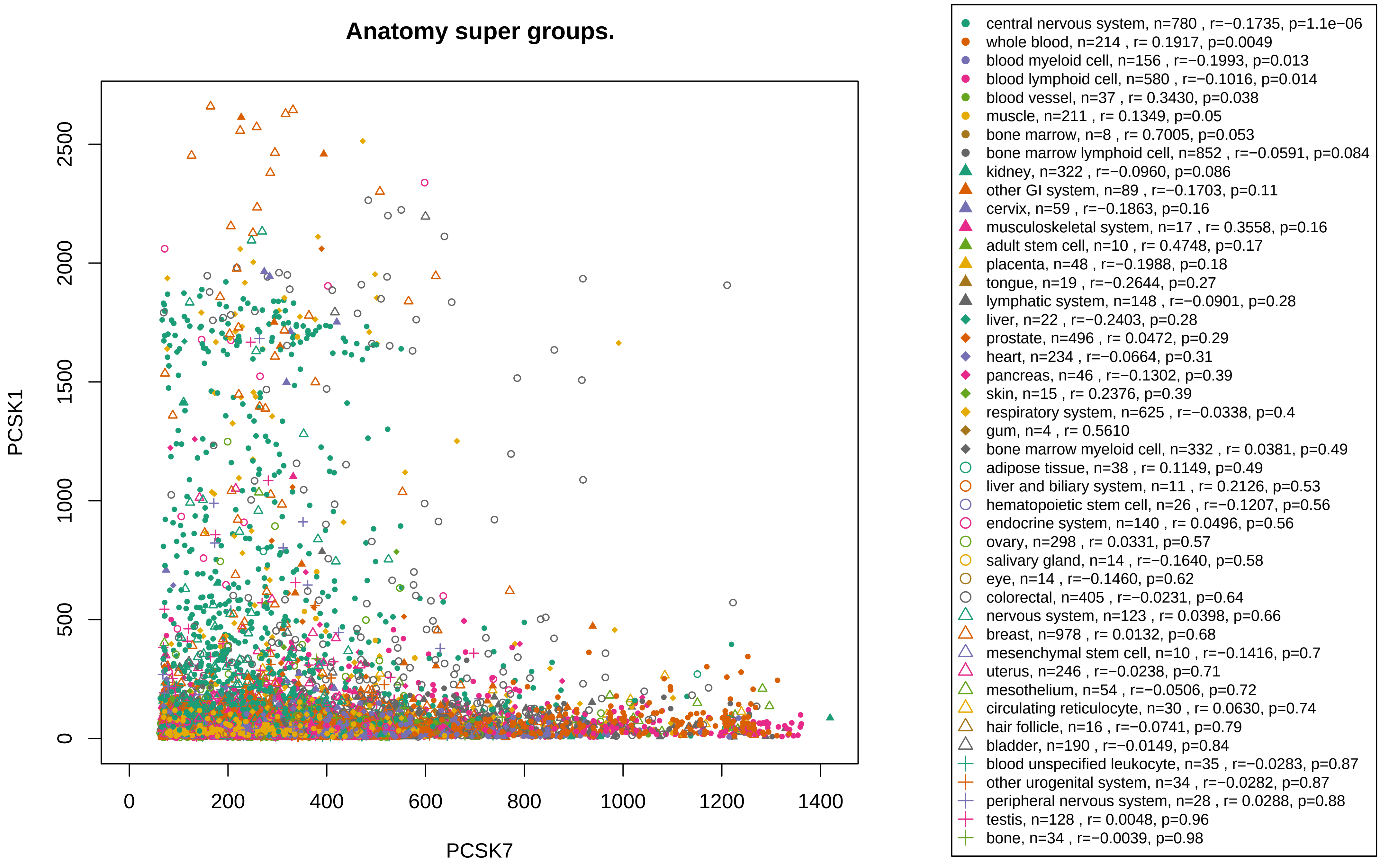
Anatomy super groups.
PCSK1
PCSK7
0
500
1000
1500
2000
2500
0
200
400
600
800
1000
1200
1400
central nervous system, n=780 , r=−0.1735, p=1.1e−06
whole blood, n=214 , r= 0.1917, p=0.0049
blood myeloid cell, n=156 , r=−0.1993, p=0.013
blood lymphoid cell, n=580 , r=−0.1016, p=0.014
blood vessel, n=37 , r= 0.3430, p=0.038
muscle, n=211 , r= 0.1349, p=0.05
bone marrow, n=8 , r= 0.7005, p=0.053
bone marrow lymphoid cell, n=852 , r=−0.0591, p=0.084
kidney, n=322 , r=−0.0960, p=0.086
other GI system, n=89 , r=−0.1703, p=0.11
cervix, n=59 , r=−0.1863, p=0.16
musculoskeletal system, n=17 , r= 0.3558, p=0.16
adult stem cell, n=10 , r= 0.4748, p=0.17
placenta, n=48 , r=−0.1988, p=0.18
tongue, n=19 , r=−0.2644, p=0.27
lymphatic system, n=148 , r=−0.0901, p=0.28
liver, n=22 , r=−0.2403, p=0.28
prostate, n=496 , r= 0.0472, p=0.29
heart, n=234 , r=−0.0664, p=0.31
pancreas, n=46 , r=−0.1302, p=0.39
skin, n=15 , r= 0.2376, p=0.39
respiratory system, n=625 , r=−0.0338, p=0.4
gum, n=4 , r= 0.5610
bone marrow myeloid cell, n=332 , r= 0.0381, p=0.49
adipose tissue, n=38 , r= 0.1149, p=0.49
liver and biliary system, n=11 , r= 0.2126, p=0.53
hematopoietic stem cell, n=26 , r=−0.1207, p=0.56
endocrine system, n=140 , r= 0.0496, p=0.56
ovary, n=298 , r= 0.0331, p=0.57
salivary gland, n=14 , r=−0.1640, p=0.58
eye, n=14 , r=−0.1460, p=0.62
colorectal, n=405 , r=−0.0231, p=0.64
nervous system, n=123 , r= 0.0398, p=0.66
breast, n=978 , r= 0.0132, p=0.68
mesenchymal stem cell, n=10 , r=−0.1416, p=0.7
uterus, n=246 , r=−0.0238, p=0.71
mesothelium, n=54 , r=−0.0506, p=0.72
circulating reticulocyte, n=30 , r= 0.0630, p=0.74
hair follicle, n=16 , r=−0.0741, p=0.79
bladder, n=190 , r=−0.0149, p=0.84
blood unspecified leukocyte, n=35 , r=−0.0283, p=0.87
other urogenital system, n=34 , r=−0.0282, p=0.87
peripheral nervous system, n=28 , r= 0.0288, p=0.88
testis, n=128 , r= 0.0048, p=0.96
bone, n=34 , r=−0.0039, p=0.98

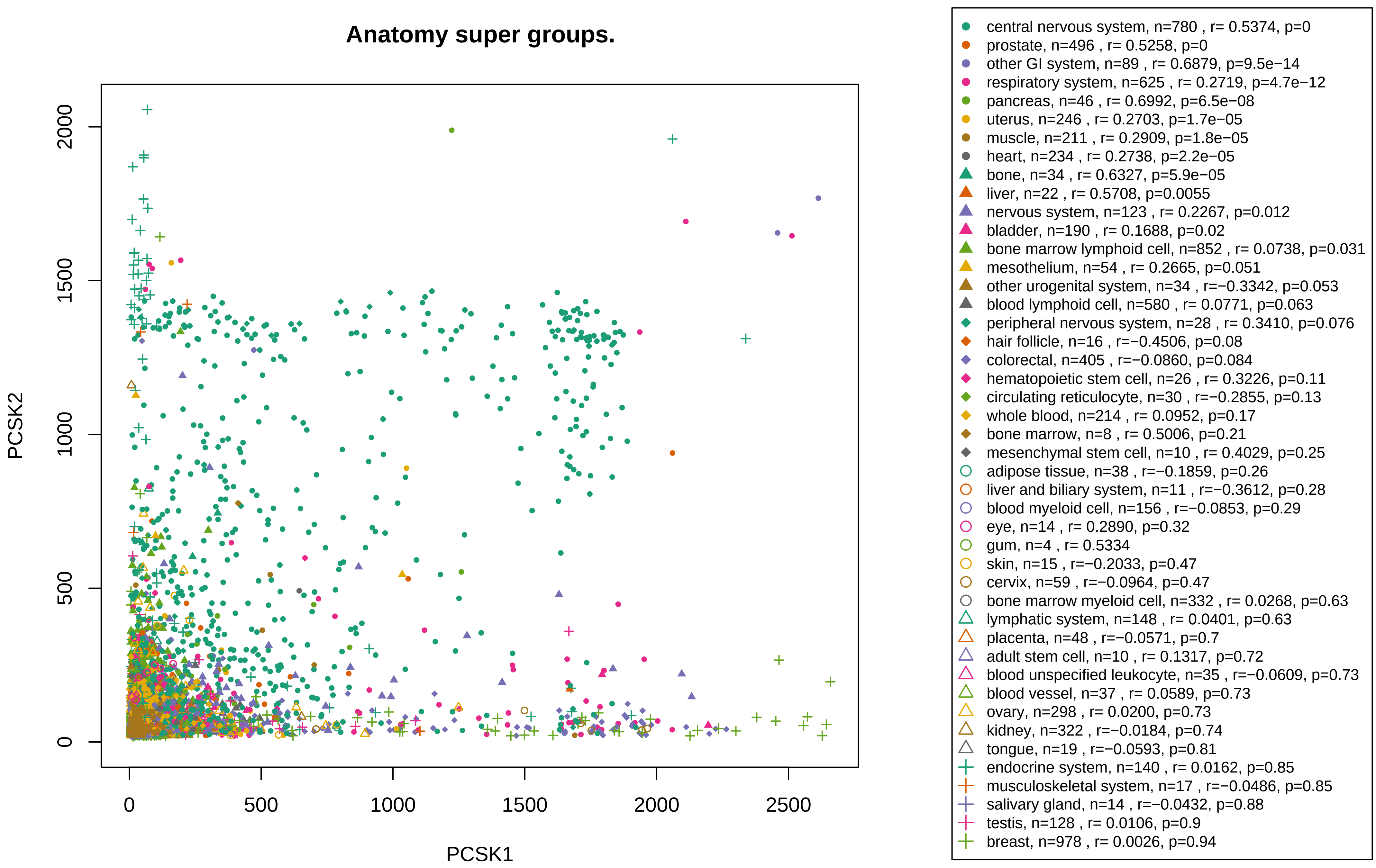

Anatomy super groups.
PCSK2
PCSK1
0
500
1000
1500
2000
2500
central nervous system, n=780 , r= 0.5374, p=0
prostate, n=496 , r= 0.5258, p=0
other GI system, n=89 , r= 0.6879, p=9.5e−14
respiratory system, n=625 , r= 0.2719, p=4.7e−12
pancreas, n=46 , r= 0.6992, p=6.5e−08
uterus, n=246 , r= 0.2703, p=1.7e−05
muscle, n=211 , r= 0.2909, p=1.8e−05
heart, n=234 , r= 0.2738, p=2.2e−05
bone, n=34 , r= 0.6327, p=5.9e−05
liver, n=22 , r= 0.5708, p=0.0055
nervous system, n=123 , r= 0.2267, p=0.012
bladder, n=190 , r= 0.1688, p=0.02
bone marrow lymphoid cell, n=852 , r= 0.0738, p=0.031
mesothelium, n=54 , r= 0.2665, p=0.051
other urogenital system, n=34 , r=−0.3342, p=0.053
blood lymphoid cell, n=580 , r= 0.0771, p=0.063
peripheral nervous system, n=28 , r= 0.3410, p=0.076
hair follicle, n=16 , r=−0.4506, p=0.08
colorectal, n=405 , r=−0.0860, p=0.084
hematopoietic stem cell, n=26 , r= 0.3226, p=0.11
circulating reticulocyte, n=30 , r=−0.2855, p=0.13
whole blood, n=214 , r= 0.0952, p=0.17
bone marrow, n=8 , r= 0.5006, p=0.21
mesenchymal stem cell, n=10 , r= 0.4029, p=0.25
adipose tissue, n=38 , r=−0.1859, p=0.26
liver and biliary system, n=11 , r=−0.3612, p=0.28
blood myeloid cell, n=156 , r=−0.0853, p=0.29
eye, n=14 , r= 0.2890, p=0.32
gum, n=4 , r= 0.5334
skin, n=15 , r=−0.2033, p=0.47
cervix, n=59 , r=−0.0964, p=0.47
bone marrow myeloid cell, n=332 , r= 0.0268, p=0.63
lymphatic system, n=148 , r= 0.0401, p=0.63
placenta, n=48 , r=−0.0571, p=0.7
adult stem cell, n=10 , r= 0.1317, p=0.72
blood unspecified leukocyte, n=35 , r=−0.0609, p=0.73
blood vessel, n=37 , r= 0.0589, p=0.73
ovary, n=298 , r= 0.0200, p=0.73
kidney, n=322 , r=−0.0184, p=0.74
tongue, n=19 , r=−0.0593, p=0.81
endocrine system, n=140 , r= 0.0162, p=0.85
musculoskeletal system, n=17 , r=−0.0486, p=0.85
salivary gland, n=14 , r=−0.0432, p=0.88
testis, n=128 , r= 0.0106, p=0.9
breast, n=978 , r= 0.0026, p=0.94

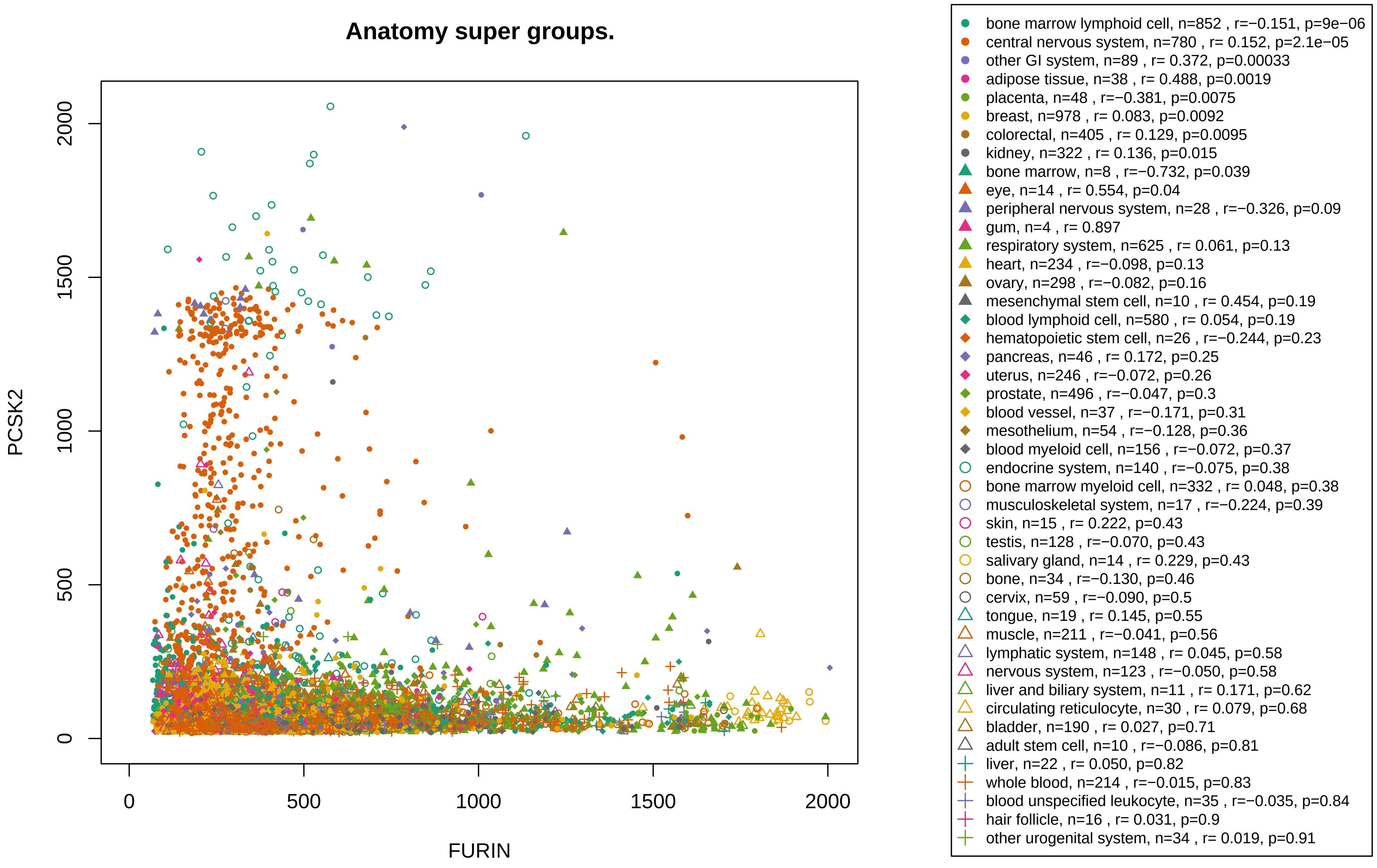

Anatomy super groups.
PCSK2
FURIN
0
500
1000
1500
2000
bone marrow lymphoid cell, n=852 , r=−0.151, p=9e−06
central nervous system, n=780 , r= 0.152, p=2.1e−05
other GI system, n=89 , r= 0.372, p=0.00033
adipose tissue, n=38 , r= 0.488, p=0.0019
placenta, n=48 , r=−0.381, p=0.0075
breast, n=978 , r= 0.083, p=0.0092
colorectal, n=405 , r= 0.129, p=0.0095
kidney, n=322 , r= 0.136, p=0.015
bone marrow, n=8 , r=−0.732, p=0.039
eye, n=14 , r= 0.554, p=0.04
peripheral nervous system, n=28 , r=−0.326, p=0.09
gum, n=4 , r= 0.897
respiratory system, n=625 , r= 0.061, p=0.13
heart, n=234 , r=−0.098, p=0.13
ovary, n=298 , r=−0.082, p=0.16
mesenchymal stem cell, n=10 , r= 0.454, p=0.19
blood lymphoid cell, n=580 , r= 0.054, p=0.19
hematopoietic stem cell, n=26 , r=−0.244, p=0.23
pancreas, n=46 , r= 0.172, p=0.25
uterus, n=246 , r=−0.072, p=0.26
prostate, n=496 , r=−0.047, p=0.3
blood vessel, n=37 , r=−0.171, p=0.31
mesothelium, n=54 , r=−0.128, p=0.36
blood myeloid cell, n=156 , r=−0.072, p=0.37
endocrine system, n=140 , r=−0.075, p=0.38
bone marrow myeloid cell, n=332 , r= 0.048, p=0.38
musculoskeletal system, n=17 , r=−0.224, p=0.39
skin, n=15 , r= 0.222, p=0.43
testis, n=128 , r=−0.070, p=0.43
salivary gland, n=14 , r= 0.229, p=0.43
bone, n=34 , r=−0.130, p=0.46
cervix, n=59 , r=−0.090, p=0.5
tongue, n=19 , r= 0.145, p=0.55
muscle, n=211 , r=−0.041, p=0.56
lymphatic system, n=148 , r= 0.045, p=0.58
nervous system, n=123 , r=−0.050, p=0.58
liver and biliary system, n=11 , r= 0.171, p=0.62
circulating reticulocyte, n=30 , r= 0.079, p=0.68
bladder, n=190 , r= 0.027, p=0.71
adult stem cell, n=10 , r=−0.086, p=0.81
liver, n=22 , r= 0.050, p=0.82
whole blood, n=214 , r=−0.015, p=0.83
blood unspecified leukocyte, n=35 , r=−0.035, p=0.84
hair follicle, n=16 , r= 0.031, p=0.9
other urogenital system, n=34 , r= 0.019, p=0.91

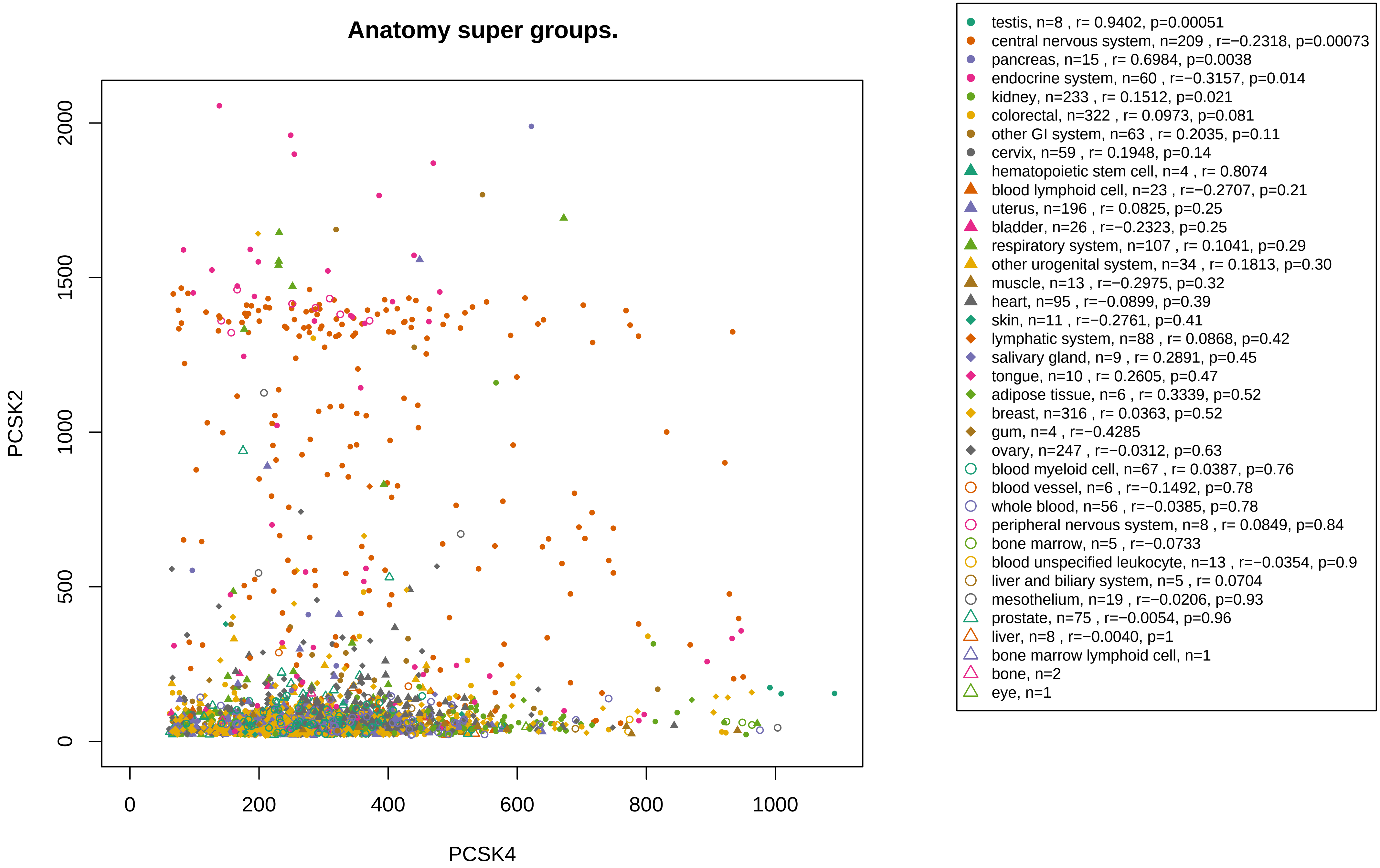
Anatomy super groups.
PCSK2
PCSK4
0
500
1000
1500
2000
0
200
400
600
800
1000
testis, n=8 , r= 0.9402, p=0.00051
central nervous system, n=209 , r=−0.2318, p=0.00073
pancreas, n=15 , r= 0.6984, p=0.0038
endocrine system, n=60 , r=−0.3157, p=0.014
kidney, n=233 , r= 0.1512, p=0.021
colorectal, n=322 , r= 0.0973, p=0.081
other GI system, n=63 , r= 0.2035, p=0.11
cervix, n=59 , r= 0.1948, p=0.14
hematopoietic stem cell, n=4 , r= 0.8074
blood lymphoid cell, n=23 , r=−0.2707, p=0.21
uterus, n=196 , r= 0.0825, p=0.25
bladder, n=26 , r=−0.2323, p=0.25
respiratory system, n=107 , r= 0.1041, p=0.29
other urogenital system, n=34 , r= 0.1813, p=0.30
muscle, n=13 , r=−0.2975, p=0.32
heart, n=95 , r=−0.0899, p=0.39
skin, n=11 , r=−0.2761, p=0.41
lymphatic system, n=88 , r= 0.0868, p=0.42
salivary gland, n=9 , r= 0.2891, p=0.45
tongue, n=10 , r= 0.2605, p=0.47
adipose tissue, n=6 , r= 0.3339, p=0.52
breast, n=316 , r= 0.0363, p=0.52
gum, n=4 , r=−0.4285
ovary, n=247 , r=−0.0312, p=0.63
blood myeloid cell, n=67 , r= 0.0387, p=0.76
blood vessel, n=6 , r=−0.1492, p=0.78
whole blood, n=56 , r=−0.0385, p=0.78
peripheral nervous system, n=8 , r= 0.0849, p=0.84
bone marrow, n=5 , r=−0.0733
blood unspecified leukocyte, n=13 , r=−0.0354, p=0.9
liver and biliary system, n=5 , r= 0.0704
mesothelium, n=19 , r=−0.0206, p=0.93
prostate, n=75 , r=−0.0054, p=0.96
liver, n=8 , r=−0.0040, p=1
bone marrow lymphoid cell, n=1
bone, n=2
eye, n=1

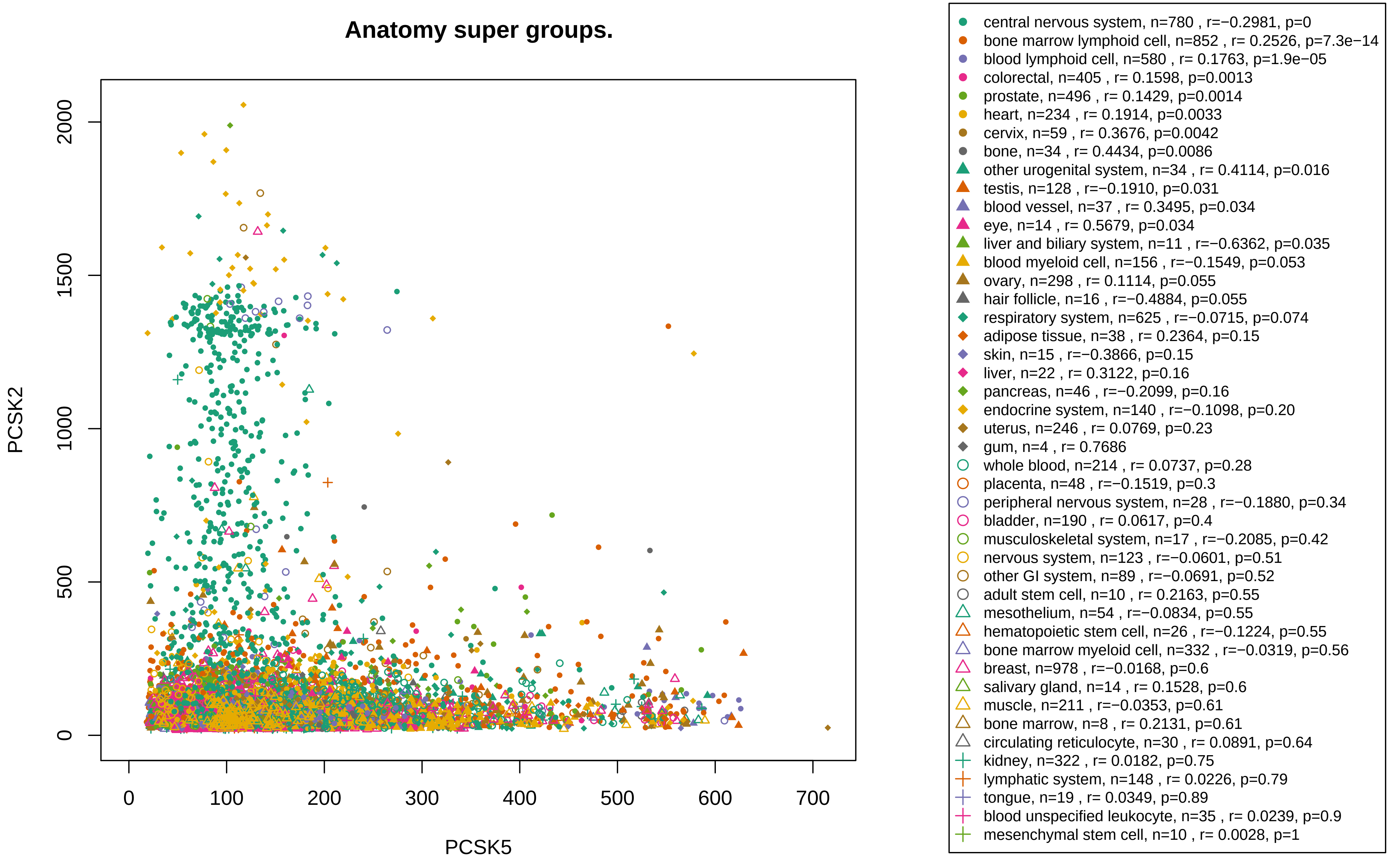
Anatomy super groups.
PCSK2
PCSK5
0
500
1000
1500
2000
0
100
200
300
400
500
600
700
central nervous system, n=780 , r=−0.2981, p=0
bone marrow lymphoid cell, n=852 , r= 0.2526, p=7.3e−14
blood lymphoid cell, n=580 , r= 0.1763, p=1.9e−05
colorectal, n=405 , r= 0.1598, p=0.0013
prostate, n=496 , r= 0.1429, p=0.0014
heart, n=234 , r= 0.1914, p=0.0033
cervix, n=59 , r= 0.3676, p=0.0042
bone, n=34 , r= 0.4434, p=0.0086
other urogenital system, n=34 , r= 0.4114, p=0.016
testis, n=128 , r=−0.1910, p=0.031
blood vessel, n=37 , r= 0.3495, p=0.034
eye, n=14 , r= 0.5679, p=0.034
liver and biliary system, n=11 , r=−0.6362, p=0.035
blood myeloid cell, n=156 , r=−0.1549, p=0.053
ovary, n=298 , r= 0.1114, p=0.055
hair follicle, n=16 , r=−0.4884, p=0.055
respiratory system, n=625 , r=−0.0715, p=0.074
adipose tissue, n=38 , r= 0.2364, p=0.15
skin, n=15 , r=−0.3866, p=0.15
liver, n=22 , r= 0.3122, p=0.16
pancreas, n=46 , r=−0.2099, p=0.16
endocrine system, n=140 , r=−0.1098, p=0.20
uterus, n=246 , r= 0.0769, p=0.23
gum, n=4 , r= 0.7686
whole blood, n=214 , r= 0.0737, p=0.28
placenta, n=48 , r=−0.1519, p=0.3
peripheral nervous system, n=28 , r=−0.1880, p=0.34
bladder, n=190 , r= 0.0617, p=0.4
musculoskeletal system, n=17 , r=−0.2085, p=0.42
nervous system, n=123 , r=−0.0601, p=0.51
other GI system, n=89 , r=−0.0691, p=0.52
adult stem cell, n=10 , r= 0.2163, p=0.55
mesothelium, n=54 , r=−0.0834, p=0.55
hematopoietic stem cell, n=26 , r=−0.1224, p=0.55
bone marrow myeloid cell, n=332 , r=−0.0319, p=0.56
breast, n=978 , r=−0.0168, p=0.6
salivary gland, n=14 , r= 0.1528, p=0.6
muscle, n=211 , r=−0.0353, p=0.61
bone marrow, n=8 , r= 0.2131, p=0.61
circulating reticulocyte, n=30 , r= 0.0891, p=0.64
kidney, n=322 , r= 0.0182, p=0.75
lymphatic system, n=148 , r= 0.0226, p=0.79
tongue, n=19 , r= 0.0349, p=0.89
blood unspecified leukocyte, n=35 , r= 0.0239, p=0.9
mesenchymal stem cell, n=10 , r= 0.0028, p=1

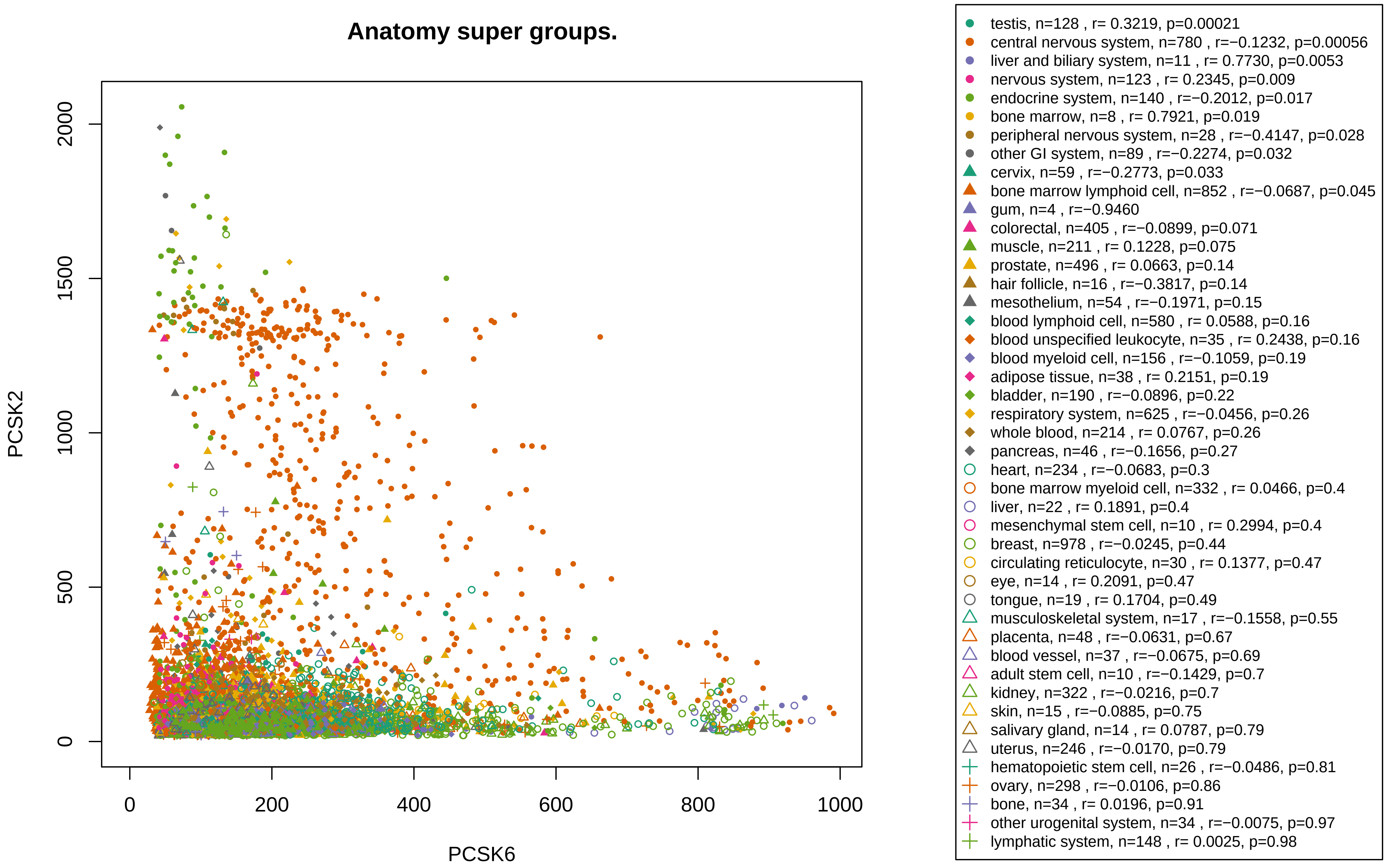
Anatomy super groups.
PCSK2
PCSK6
0
500
1000
1500
2000
0
200
400
600
800
1000
testis, n=128 , r= 0.3219, p=0.00021
central nervous system, n=780 , r=−0.1232, p=0.00056
liver and biliary system, n=11 , r= 0.7730, p=0.0053
nervous system, n=123 , r= 0.2345, p=0.009
endocrine system, n=140 , r=−0.2012, p=0.017
bone marrow, n=8 , r= 0.7921, p=0.019
peripheral nervous system, n=28 , r=−0.4147, p=0.028
other GI system, n=89 , r=−0.2274, p=0.032
cervix, n=59 , r=−0.2773, p=0.033
bone marrow lymphoid cell, n=852 , r=−0.0687, p=0.045
gum, n=4 , r=−0.9460
colorectal, n=405 , r=−0.0899, p=0.071
muscle, n=211 , r= 0.1228, p=0.075
prostate, n=496 , r= 0.0663, p=0.14
hair follicle, n=16 , r=−0.3817, p=0.14
mesothelium, n=54 , r=−0.1971, p=0.15
blood lymphoid cell, n=580 , r= 0.0588, p=0.16
blood unspecified leukocyte, n=35 , r= 0.2438, p=0.16
blood myeloid cell, n=156 , r=−0.1059, p=0.19
adipose tissue, n=38 , r= 0.2151, p=0.19
bladder, n=190 , r=−0.0896, p=0.22
respiratory system, n=625 , r=−0.0456, p=0.26
whole blood, n=214 , r= 0.0767, p=0.26
pancreas, n=46 , r=−0.1656, p=0.27
heart, n=234 , r=−0.0683, p=0.3
bone marrow myeloid cell, n=332 , r= 0.0466, p=0.4
liver, n=22 , r= 0.1891, p=0.4
mesenchymal stem cell, n=10 , r= 0.2994, p=0.4
breast, n=978 , r=−0.0245, p=0.44
circulating reticulocyte, n=30 , r= 0.1377, p=0.47
eye, n=14 , r= 0.2091, p=0.47
tongue, n=19 , r= 0.1704, p=0.49
musculoskeletal system, n=17 , r=−0.1558, p=0.55
placenta, n=48 , r=−0.0631, p=0.67
blood vessel, n=37 , r=−0.0675, p=0.69
adult stem cell, n=10 , r=−0.1429, p=0.7
kidney, n=322 , r=−0.0216, p=0.7
skin, n=15 , r=−0.0885, p=0.75
salivary gland, n=14 , r= 0.0787, p=0.79
uterus, n=246 , r=−0.0170, p=0.79
hematopoietic stem cell, n=26 , r=−0.0486, p=0.81
ovary, n=298 , r=−0.0106, p=0.86
bone, n=34 , r= 0.0196, p=0.91
other urogenital system, n=34 , r=−0.0075, p=0.97
lymphatic system, n=148 , r= 0.0025, p=0.98

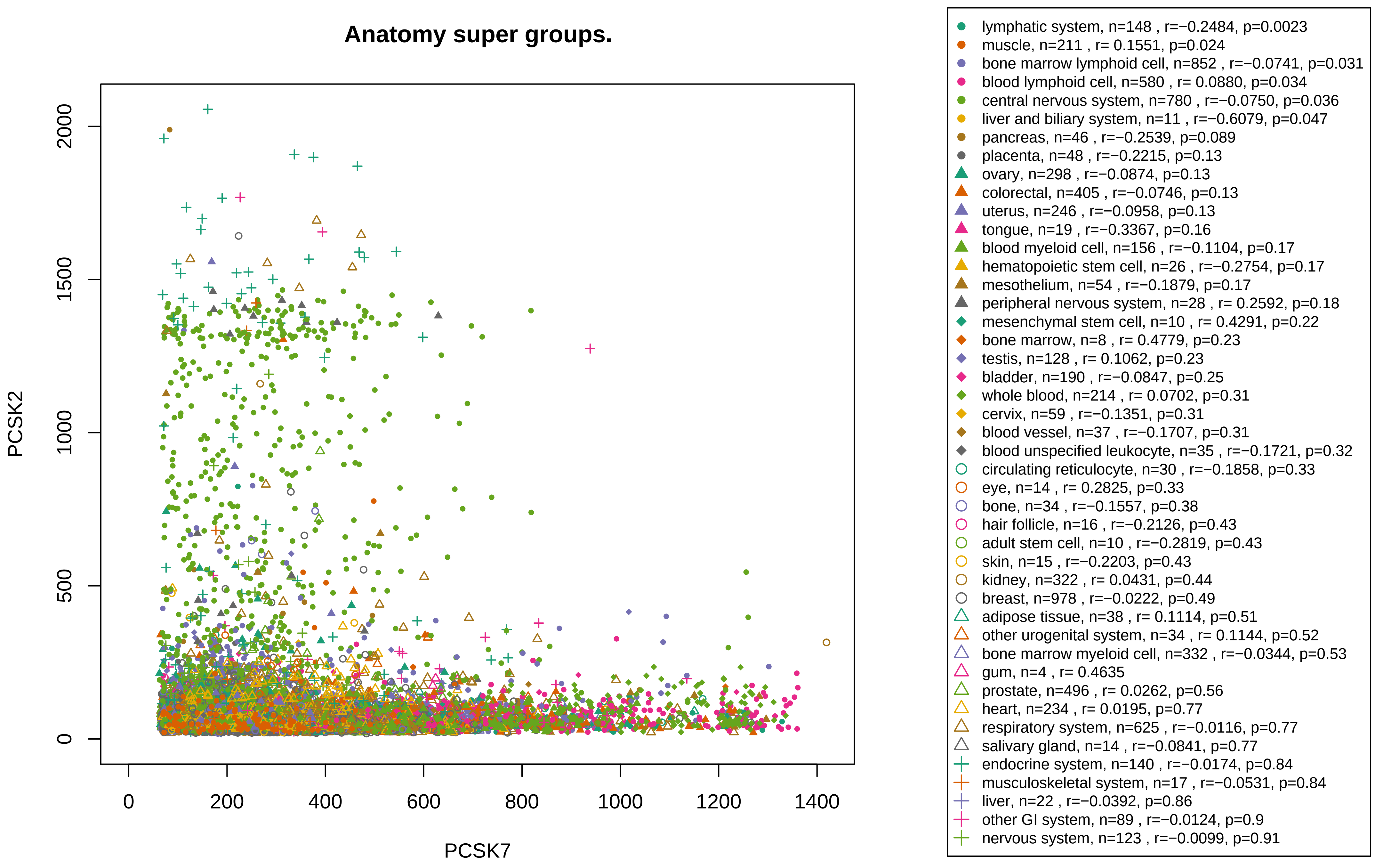

Anatomy super groups.
PCSK2
PCSK7
0
500
1000
1500
2000
0
200
400
600
800
1000
1200
1400
lymphatic system, n=148 , r=−0.2484, p=0.0023
muscle, n=211 , r= 0.1551, p=0.024
bone marrow lymphoid cell, n=852 , r=−0.0741, p=0.031
blood lymphoid cell, n=580 , r= 0.0880, p=0.034
central nervous system, n=780 , r=−0.0750, p=0.036
liver and biliary system, n=11 , r=−0.6079, p=0.047
pancreas, n=46 , r=−0.2539, p=0.089
placenta, n=48 , r=−0.2215, p=0.13
ovary, n=298 , r=−0.0874, p=0.13
colorectal, n=405 , r=−0.0746, p=0.13
uterus, n=246 , r=−0.0958, p=0.13
tongue, n=19 , r=−0.3367, p=0.16
blood myeloid cell, n=156 , r=−0.1104, p=0.17
hematopoietic stem cell, n=26 , r=−0.2754, p=0.17
mesothelium, n=54 , r=−0.1879, p=0.17
peripheral nervous system, n=28 , r= 0.2592, p=0.18
mesenchymal stem cell, n=10 , r= 0.4291, p=0.22
bone marrow, n=8 , r= 0.4779, p=0.23
testis, n=128 , r= 0.1062, p=0.23
bladder, n=190 , r=−0.0847, p=0.25
whole blood, n=214 , r= 0.0702, p=0.31
cervix, n=59 , r=−0.1351, p=0.31
blood vessel, n=37 , r=−0.1707, p=0.31
blood unspecified leukocyte, n=35 , r=−0.1721, p=0.32
circulating reticulocyte, n=30 , r=−0.1858, p=0.33
eye, n=14 , r= 0.2825, p=0.33
bone, n=34 , r=−0.1557, p=0.38
hair follicle, n=16 , r=−0.2126, p=0.43
adult stem cell, n=10 , r=−0.2819, p=0.43
skin, n=15 , r=−0.2203, p=0.43
kidney, n=322 , r= 0.0431, p=0.44
breast, n=978 , r=−0.0222, p=0.49
adipose tissue, n=38 , r= 0.1114, p=0.51
other urogenital system, n=34 , r= 0.1144, p=0.52
bone marrow myeloid cell, n=332 , r=−0.0344, p=0.53
gum, n=4 , r= 0.4635
prostate, n=496 , r= 0.0262, p=0.56
heart, n=234 , r= 0.0195, p=0.77
respiratory system, n=625 , r=−0.0116, p=0.77
salivary gland, n=14 , r=−0.0841, p=0.77
endocrine system, n=140 , r=−0.0174, p=0.84
musculoskeletal system, n=17 , r=−0.0531, p=0.84
liver, n=22 , r=−0.0392, p=0.86
other GI system, n=89 , r=−0.0124, p=0.9
nervous system, n=123 , r=−0.0099, p=0.91

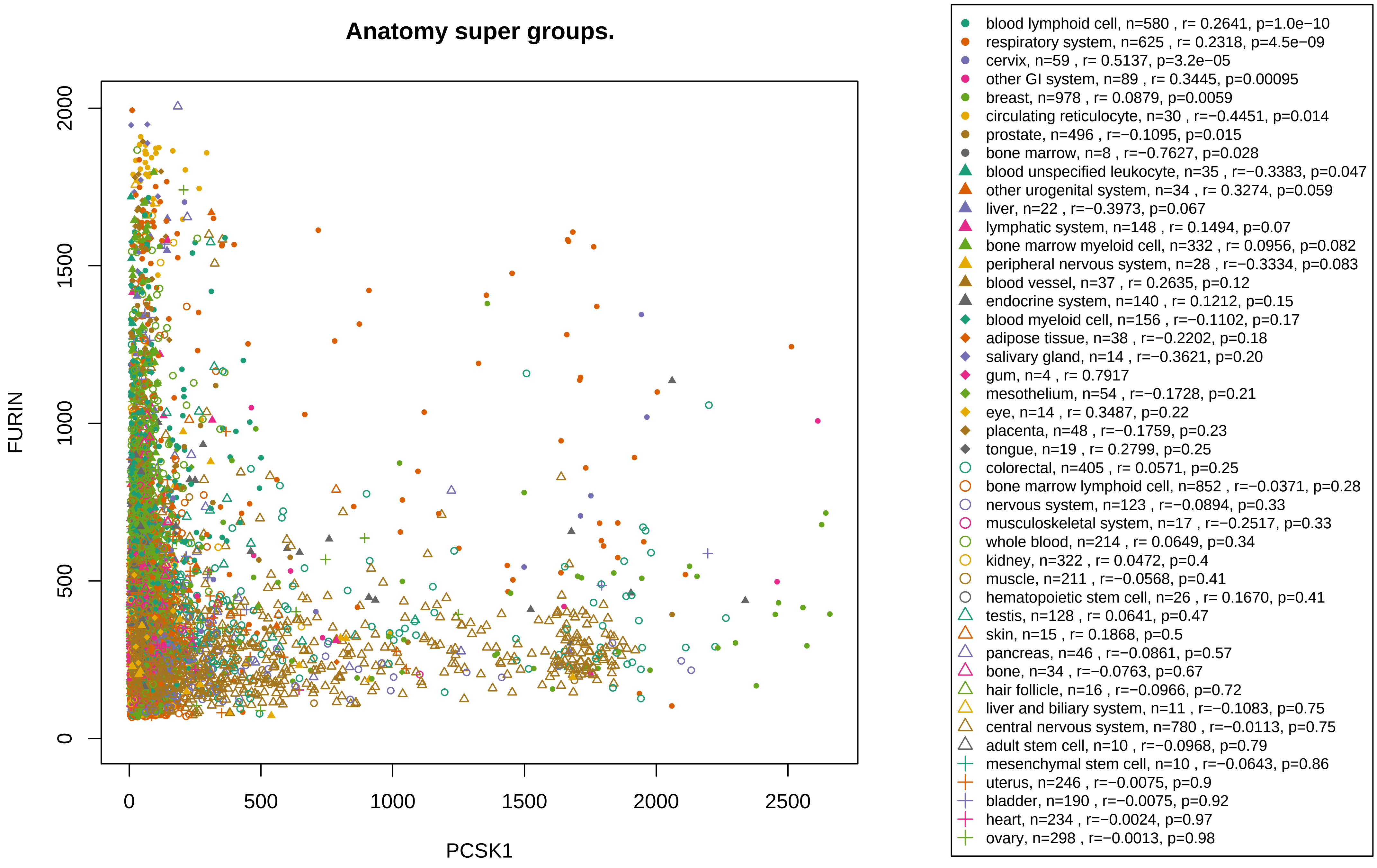
Anatomy super groups.
FURIN
PCSK1
0
500
1000
1500
2000
2500
blood lymphoid cell, n=580 , r= 0.2641, p=1.0e−10
respiratory system, n=625 , r= 0.2318, p=4.5e−09
cervix, n=59 , r= 0.5137, p=3.2e−05
other GI system, n=89 , r= 0.3445, p=0.00095
breast, n=978 , r= 0.0879, p=0.0059
circulating reticulocyte, n=30 , r=−0.4451, p=0.014
prostate, n=496 , r=−0.1095, p=0.015
bone marrow, n=8 , r=−0.7627, p=0.028
blood unspecified leukocyte, n=35 , r=−0.3383, p=0.047
other urogenital system, n=34 , r= 0.3274, p=0.059
liver, n=22 , r=−0.3973, p=0.067
lymphatic system, n=148 , r= 0.1494, p=0.07
bone marrow myeloid cell, n=332 , r= 0.0956, p=0.082
peripheral nervous system, n=28 , r=−0.3334, p=0.083
blood vessel, n=37 , r= 0.2635, p=0.12
endocrine system, n=140 , r= 0.1212, p=0.15
blood myeloid cell, n=156 , r=−0.1102, p=0.17
adipose tissue, n=38 , r=−0.2202, p=0.18
salivary gland, n=14 , r=−0.3621, p=0.20
gum, n=4 , r= 0.7917
mesothelium, n=54 , r=−0.1728, p=0.21
eye, n=14 , r= 0.3487, p=0.22
placenta, n=48 , r=−0.1759, p=0.23
tongue, n=19 , r= 0.2799, p=0.25
colorectal, n=405 , r= 0.0571, p=0.25
bone marrow lymphoid cell, n=852 , r=−0.0371, p=0.28
nervous system, n=123 , r=−0.0894, p=0.33
musculoskeletal system, n=17 , r=−0.2517, p=0.33
whole blood, n=214 , r= 0.0649, p=0.34
kidney, n=322 , r= 0.0472, p=0.4
muscle, n=211 , r=−0.0568, p=0.41
hematopoietic stem cell, n=26 , r= 0.1670, p=0.41
testis, n=128 , r= 0.0641, p=0.47
skin, n=15 , r= 0.1868, p=0.5
pancreas, n=46 , r=−0.0861, p=0.57
bone, n=34 , r=−0.0763, p=0.67
hair follicle, n=16 , r=−0.0966, p=0.72
liver and biliary system, n=11 , r=−0.1083, p=0.75
central nervous system, n=780 , r=−0.0113, p=0.75
adult stem cell, n=10 , r=−0.0968, p=0.79
mesenchymal stem cell, n=10 , r=−0.0643, p=0.86
uterus, n=246 , r=−0.0075, p=0.9
bladder, n=190 , r=−0.0075, p=0.92
heart, n=234 , r=−0.0024, p=0.97
ovary, n=298 , r=−0.0013, p=0.98

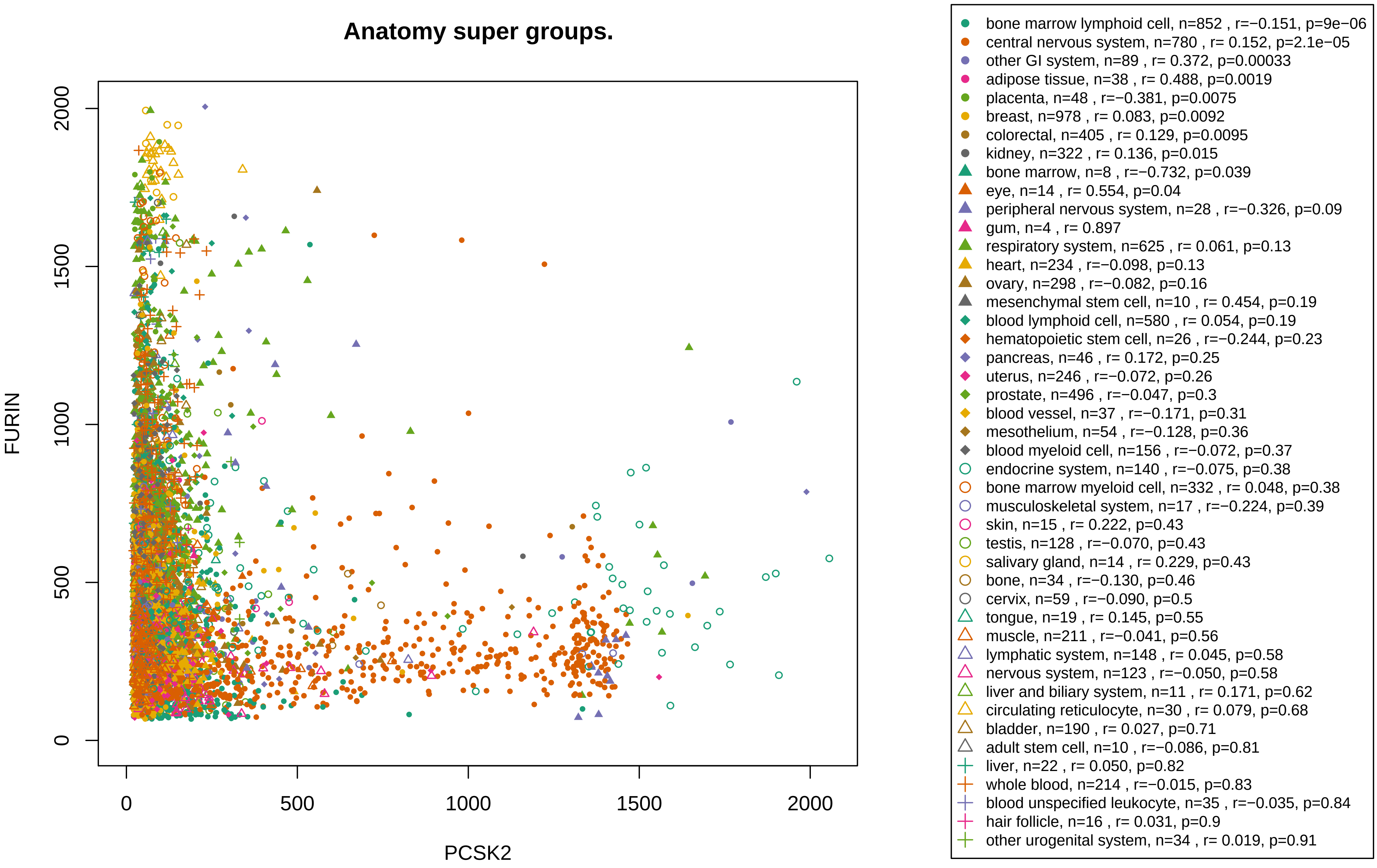
Anatomy super groups.
FURIN
PCSK2
0
500
1000
1500
2000
0
500
1000
1500
2000
bone marrow lymphoid cell, n=852 , r=−0.151, p=9e−06
central nervous system, n=780 , r= 0.152, p=2.1e−05
other GI system, n=89 , r= 0.372, p=0.00033
adipose tissue, n=38 , r= 0.488, p=0.0019
placenta, n=48 , r=−0.381, p=0.0075
breast, n=978 , r= 0.083, p=0.0092
colorectal, n=405 , r= 0.129, p=0.0095
kidney, n=322 , r= 0.136, p=0.015
bone marrow, n=8 , r=−0.732, p=0.039
eye, n=14 , r= 0.554, p=0.04
peripheral nervous system, n=28 , r=−0.326, p=0.09
gum, n=4 , r= 0.897
respiratory system, n=625 , r= 0.061, p=0.13
heart, n=234 , r=−0.098, p=0.13
ovary, n=298 , r=−0.082, p=0.16
mesenchymal stem cell, n=10 , r= 0.454, p=0.19
blood lymphoid cell, n=580 , r= 0.054, p=0.19
hematopoietic stem cell, n=26 , r=−0.244, p=0.23
pancreas, n=46 , r= 0.172, p=0.25
uterus, n=246 , r=−0.072, p=0.26
prostate, n=496 , r=−0.047, p=0.3
blood vessel, n=37 , r=−0.171, p=0.31
mesothelium, n=54 , r=−0.128, p=0.36
blood myeloid cell, n=156 , r=−0.072, p=0.37
endocrine system, n=140 , r=−0.075, p=0.38
bone marrow myeloid cell, n=332 , r= 0.048, p=0.38
musculoskeletal system, n=17 , r=−0.224, p=0.39
skin, n=15 , r= 0.222, p=0.43
testis, n=128 , r=−0.070, p=0.43
salivary gland, n=14 , r= 0.229, p=0.43
bone, n=34 , r=−0.130, p=0.46
cervix, n=59 , r=−0.090, p=0.5
tongue, n=19 , r= 0.145, p=0.55
muscle, n=211 , r=−0.041, p=0.56
lymphatic system, n=148 , r= 0.045, p=0.58
nervous system, n=123 , r=−0.050, p=0.58
liver and biliary system, n=11 , r= 0.171, p=0.62
circulating reticulocyte, n=30 , r= 0.079, p=0.68
bladder, n=190 , r= 0.027, p=0.71
adult stem cell, n=10 , r=−0.086, p=0.81
liver, n=22 , r= 0.050, p=0.82
whole blood, n=214 , r=−0.015, p=0.83
blood unspecified leukocyte, n=35 , r=−0.035, p=0.84
hair follicle, n=16 , r= 0.031, p=0.9
other urogenital system, n=34 , r= 0.019, p=0.91

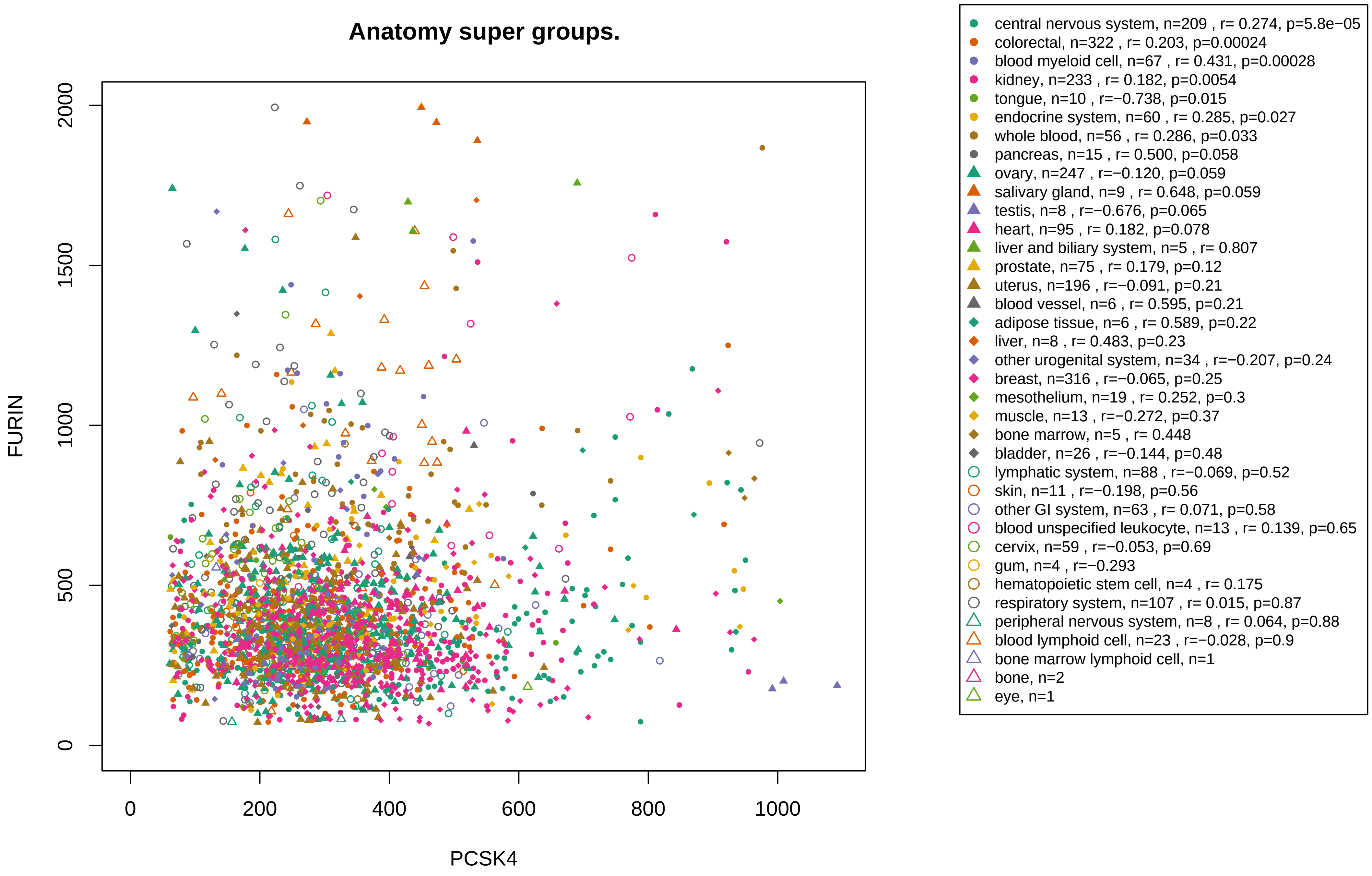

Anatomy super groups.
FURIN
PCSK4
0
500
1000
1500
2000
0
200
400
600
800
1000
central nervous system, n=209 , r= 0.274, p=5.8e−05
colorectal, n=322 , r= 0.203, p=0.00024
blood myeloid cell, n=67 , r= 0.431, p=0.00028
kidney, n=233 , r= 0.182, p=0.0054
tongue, n=10 , r=−0.738, p=0.015
endocrine system, n=60 , r= 0.285, p=0.027
whole blood, n=56 , r= 0.286, p=0.033
pancreas, n=15 , r= 0.500, p=0.058
ovary, n=247 , r=−0.120, p=0.059
salivary gland, n=9 , r= 0.648, p=0.059
testis, n=8 , r=−0.676, p=0.065
heart, n=95 , r= 0.182, p=0.078
liver and biliary system, n=5 , r= 0.807
prostate, n=75 , r= 0.179, p=0.12
uterus, n=196 , r=−0.091, p=0.21
blood vessel, n=6 , r= 0.595, p=0.21
adipose tissue, n=6 , r= 0.589, p=0.22
liver, n=8 , r= 0.483, p=0.23
other urogenital system, n=34 , r=−0.207, p=0.24
breast, n=316 , r=−0.065, p=0.25
mesothelium, n=19 , r= 0.252, p=0.3
muscle, n=13 , r=−0.272, p=0.37
bone marrow, n=5 , r= 0.448
bladder, n=26 , r=−0.144, p=0.48
lymphatic system, n=88 , r=−0.069, p=0.52
skin, n=11 , r=−0.198, p=0.56
other GI system, n=63 , r= 0.071, p=0.58
blood unspecified leukocyte, n=13 , r= 0.139, p=0.65
cervix, n=59 , r=−0.053, p=0.69
gum, n=4 , r=−0.293
hematopoietic stem cell, n=4 , r= 0.175
respiratory system, n=107 , r= 0.015, p=0.87
peripheral nervous system, n=8 , r= 0.064, p=0.88
blood lymphoid cell, n=23 , r=−0.028, p=0.9
bone marrow lymphoid cell, n=1
bone, n=2
eye, n=1

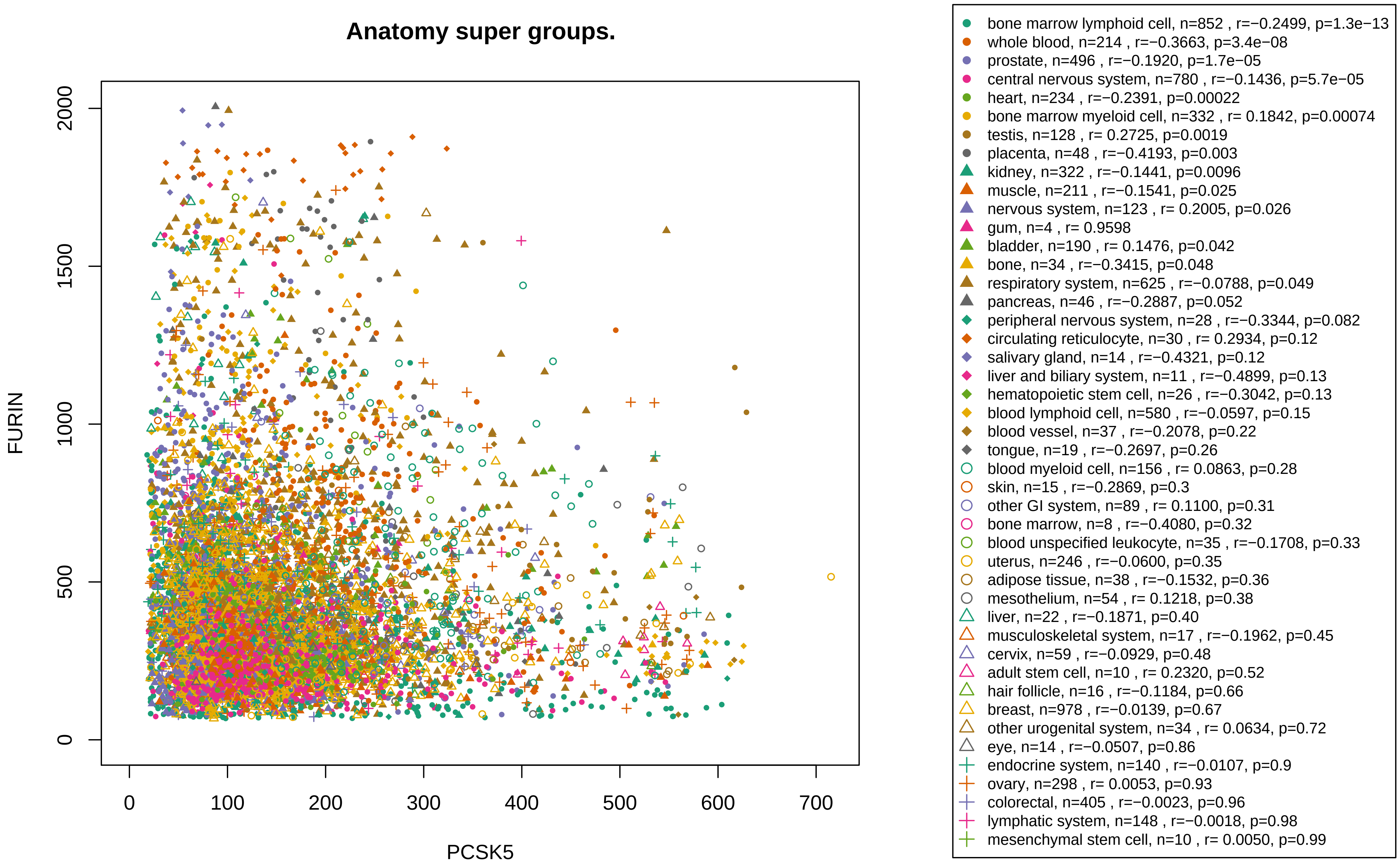
Anatomy super groups.
FURIN
PCSK5
0
500
1000
1500
2000
0
100
200
300
400
500
600
700
bone marrow lymphoid cell, n=852 , r=−0.2499, p=1.3e−13
whole blood, n=214 , r=−0.3663, p=3.4e−08
prostate, n=496 , r=−0.1920, p=1.7e−05
central nervous system, n=780 , r=−0.1436, p=5.7e−05
heart, n=234 , r=−0.2391, p=0.00022
bone marrow myeloid cell, n=332 , r= 0.1842, p=0.00074
testis, n=128 , r= 0.2725, p=0.0019
placenta, n=48 , r=−0.4193, p=0.003
kidney, n=322 , r=−0.1441, p=0.0096
muscle, n=211 , r=−0.1541, p=0.025
nervous system, n=123 , r= 0.2005, p=0.026
gum, n=4 , r= 0.9598
bladder, n=190 , r= 0.1476, p=0.042
bone, n=34 , r=−0.3415, p=0.048
respiratory system, n=625 , r=−0.0788, p=0.049
pancreas, n=46 , r=−0.2887, p=0.052
peripheral nervous system, n=28 , r=−0.3344, p=0.082
circulating reticulocyte, n=30 , r= 0.2934, p=0.12
salivary gland, n=14 , r=−0.4321, p=0.12
liver and biliary system, n=11 , r=−0.4899, p=0.13
hematopoietic stem cell, n=26 , r=−0.3042, p=0.13
blood lymphoid cell, n=580 , r=−0.0597, p=0.15
blood vessel, n=37 , r=−0.2078, p=0.22
tongue, n=19 , r=−0.2697, p=0.26
blood myeloid cell, n=156 , r= 0.0863, p=0.28
skin, n=15 , r=−0.2869, p=0.3
other GI system, n=89 , r= 0.1100, p=0.31
bone marrow, n=8 , r=−0.4080, p=0.32
blood unspecified leukocyte, n=35 , r=−0.1708, p=0.33
uterus, n=246 , r=−0.0600, p=0.35
adipose tissue, n=38 , r=−0.1532, p=0.36
mesothelium, n=54 , r= 0.1218, p=0.38
liver, n=22 , r=−0.1871, p=0.40
musculoskeletal system, n=17 , r=−0.1962, p=0.45
cervix, n=59 , r=−0.0929, p=0.48
adult stem cell, n=10 , r= 0.2320, p=0.52
hair follicle, n=16 , r=−0.1184, p=0.66
breast, n=978 , r=−0.0139, p=0.67
other urogenital system, n=34 , r= 0.0634, p=0.72
eye, n=14 , r=−0.0507, p=0.86
endocrine system, n=140 , r=−0.0107, p=0.9
ovary, n=298 , r= 0.0053, p=0.93
colorectal, n=405 , r=−0.0023, p=0.96
lymphatic system, n=148 , r=−0.0018, p=0.98
mesenchymal stem cell, n=10 , r= 0.0050, p=0.99

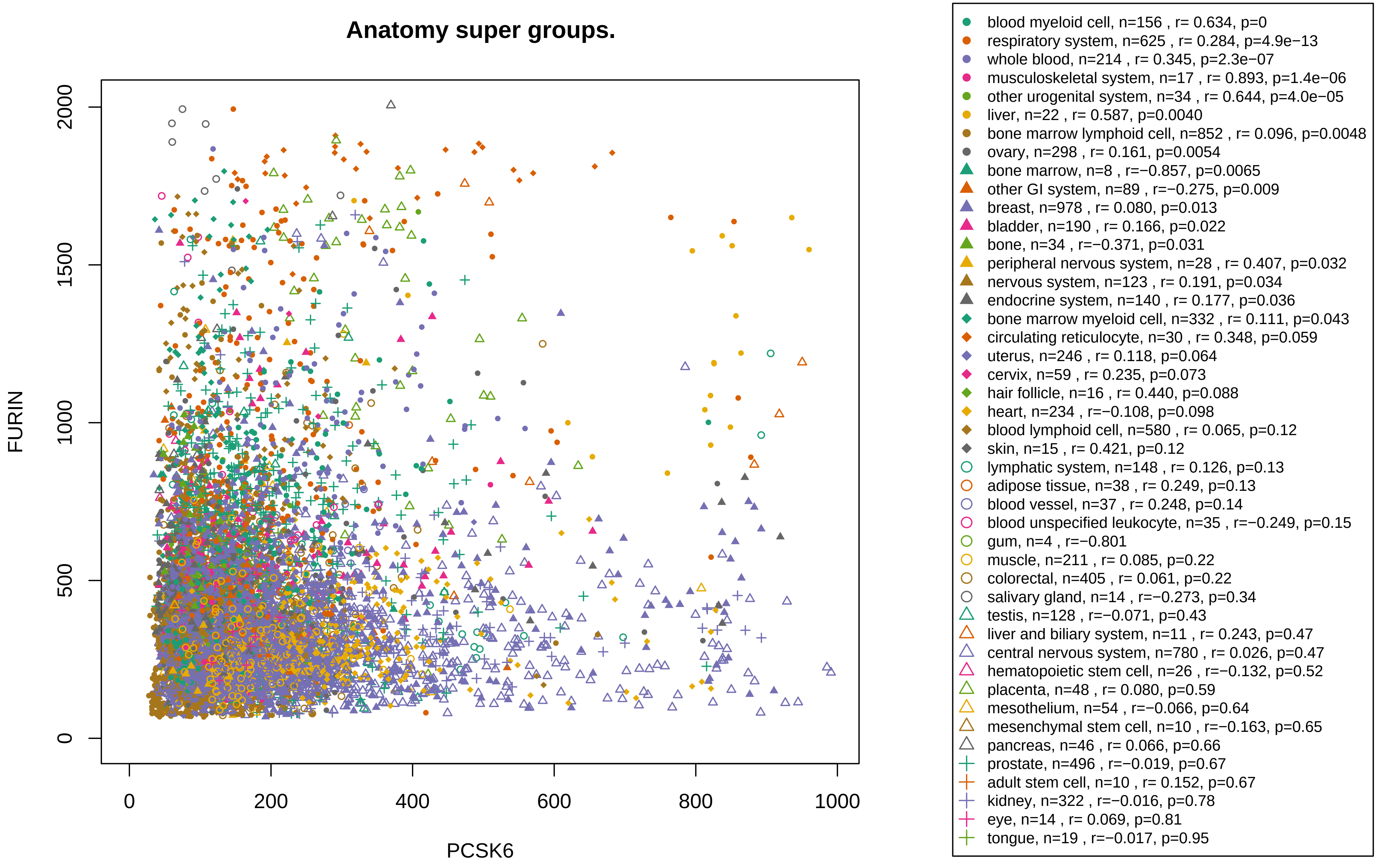
Anatomy super groups.
FURIN
PCSK6
0
500
1000
1500
2000
0
200
400
600
800
1000
blood myeloid cell, n=156 , r= 0.634, p=0
respiratory system, n=625 , r= 0.284, p=4.9e−13
whole blood, n=214 , r= 0.345, p=2.3e−07
musculoskeletal system, n=17 , r= 0.893, p=1.4e−06
other urogenital system, n=34 , r= 0.644, p=4.0e−05
liver, n=22 , r= 0.587, p=0.0040
bone marrow lymphoid cell, n=852 , r= 0.096, p=0.0048
ovary, n=298 , r= 0.161, p=0.0054
bone marrow, n=8 , r=−0.857, p=0.0065
other GI system, n=89 , r=−0.275, p=0.009
breast, n=978 , r= 0.080, p=0.013
bladder, n=190 , r= 0.166, p=0.022
bone, n=34 , r=−0.371, p=0.031
peripheral nervous system, n=28 , r= 0.407, p=0.032
nervous system, n=123 , r= 0.191, p=0.034
endocrine system, n=140 , r= 0.177, p=0.036
bone marrow myeloid cell, n=332 , r= 0.111, p=0.043
circulating reticulocyte, n=30 , r= 0.348, p=0.059
uterus, n=246 , r= 0.118, p=0.064
cervix, n=59 , r= 0.235, p=0.073
hair follicle, n=16 , r= 0.440, p=0.088
heart, n=234 , r=−0.108, p=0.098
blood lymphoid cell, n=580 , r= 0.065, p=0.12
skin, n=15 , r= 0.421, p=0.12
lymphatic system, n=148 , r= 0.126, p=0.13
adipose tissue, n=38 , r= 0.249, p=0.13
blood vessel, n=37 , r= 0.248, p=0.14
blood unspecified leukocyte, n=35 , r=−0.249, p=0.15
gum, n=4 , r=−0.801
muscle, n=211 , r= 0.085, p=0.22
colorectal, n=405 , r= 0.061, p=0.22
salivary gland, n=14 , r=−0.273, p=0.34
testis, n=128 , r=−0.071, p=0.43
liver and biliary system, n=11 , r= 0.243, p=0.47
central nervous system, n=780 , r= 0.026, p=0.47
hematopoietic stem cell, n=26 , r=−0.132, p=0.52
placenta, n=48 , r= 0.080, p=0.59
mesothelium, n=54 , r=−0.066, p=0.64
mesenchymal stem cell, n=10 , r=−0.163, p=0.65
pancreas, n=46 , r= 0.066, p=0.66
prostate, n=496 , r=−0.019, p=0.67
adult stem cell, n=10 , r= 0.152, p=0.67
kidney, n=322 , r=−0.016, p=0.78
eye, n=14 , r= 0.069, p=0.81
tongue, n=19 , r=−0.017, p=0.95

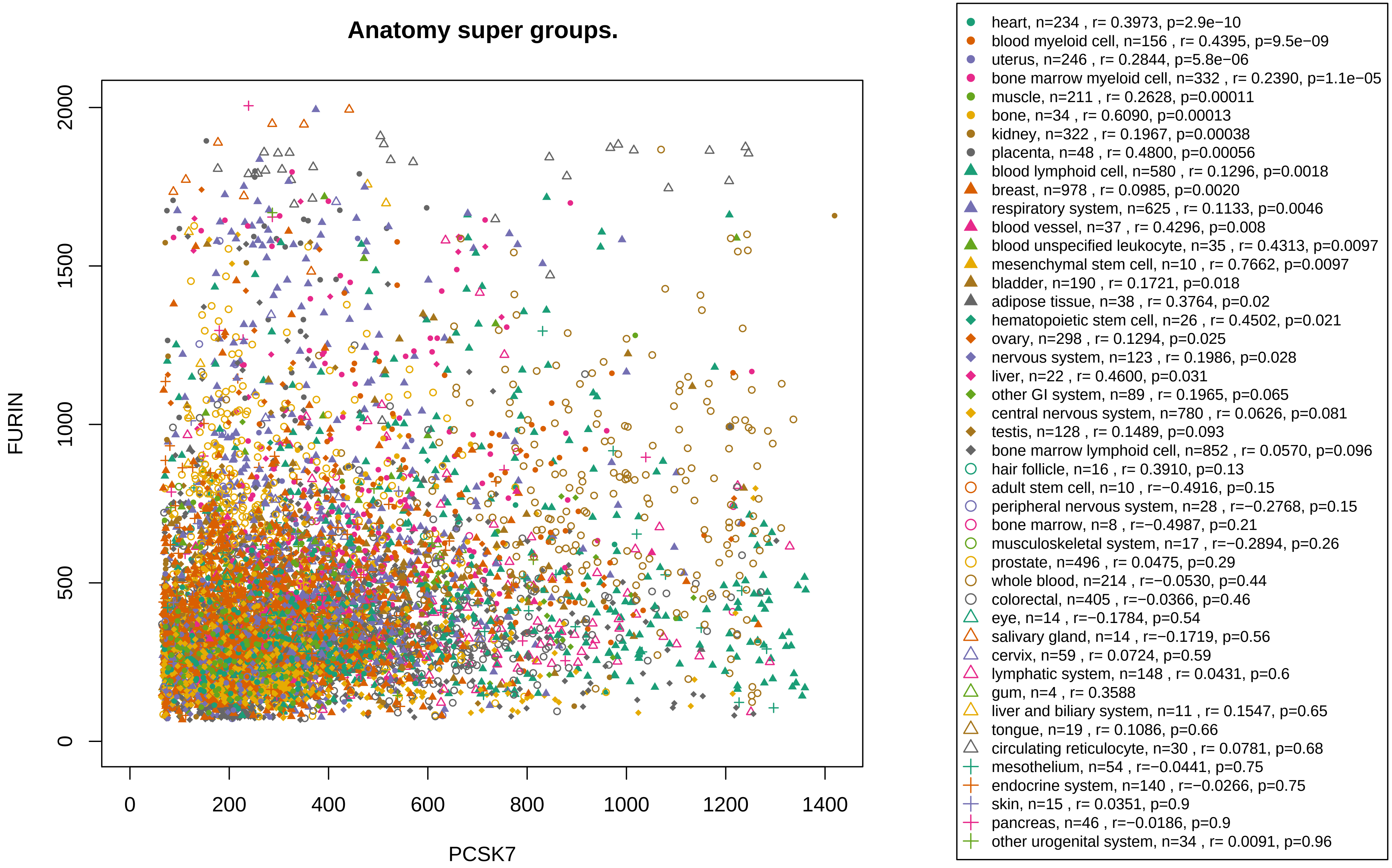

Anatomy super groups.
FURIN
PCSK7
0
500
1000
1500
2000
0
200
400
600
800
1000
1200
1400
heart, n=234 , r= 0.3973, p=2.9e−10
blood myeloid cell, n=156 , r= 0.4395, p=9.5e−09
uterus, n=246 , r= 0.2844, p=5.8e−06
bone marrow myeloid cell, n=332 , r= 0.2390, p=1.1e−05
muscle, n=211 , r= 0.2628, p=0.00011
bone, n=34 , r= 0.6090, p=0.00013
kidney, n=322 , r= 0.1967, p=0.00038
placenta, n=48 , r= 0.4800, p=0.00056
blood lymphoid cell, n=580 , r= 0.1296, p=0.0018
breast, n=978 , r= 0.0985, p=0.0020
respiratory system, n=625 , r= 0.1133, p=0.0046
blood vessel, n=37 , r= 0.4296, p=0.008
blood unspecified leukocyte, n=35 , r= 0.4313, p=0.0097
mesenchymal stem cell, n=10 , r= 0.7662, p=0.0097
bladder, n=190 , r= 0.1721, p=0.018
adipose tissue, n=38 , r= 0.3764, p=0.02
hematopoietic stem cell, n=26 , r= 0.4502, p=0.021
ovary, n=298 , r= 0.1294, p=0.025
nervous system, n=123 , r= 0.1986, p=0.028
liver, n=22 , r= 0.4600, p=0.031
other GI system, n=89 , r= 0.1965, p=0.065
central nervous system, n=780 , r= 0.0626, p=0.081
testis, n=128 , r= 0.1489, p=0.093
bone marrow lymphoid cell, n=852 , r= 0.0570, p=0.096
hair follicle, n=16 , r= 0.3910, p=0.13
adult stem cell, n=10 , r=−0.4916, p=0.15
peripheral nervous system, n=28 , r=−0.2768, p=0.15
bone marrow, n=8 , r=−0.4987, p=0.21
musculoskeletal system, n=17 , r=−0.2894, p=0.26
prostate, n=496 , r= 0.0475, p=0.29
whole blood, n=214 , r=−0.0530, p=0.44
colorectal, n=405 , r=−0.0366, p=0.46
eye, n=14 , r=−0.1784, p=0.54
salivary gland, n=14 , r=−0.1719, p=0.56
cervix, n=59 , r= 0.0724, p=0.59
lymphatic system, n=148 , r= 0.0431, p=0.6
gum, n=4 , r= 0.3588
liver and biliary system, n=11 , r= 0.1547, p=0.65
tongue, n=19 , r= 0.1086, p=0.66
circulating reticulocyte, n=30 , r= 0.0781, p=0.68
mesothelium, n=54 , r=−0.0441, p=0.75
endocrine system, n=140 , r=−0.0266, p=0.75
skin, n=15 , r= 0.0351, p=0.9
pancreas, n=46 , r=−0.0186, p=0.9
other urogenital system, n=34 , r= 0.0091, p=0.96

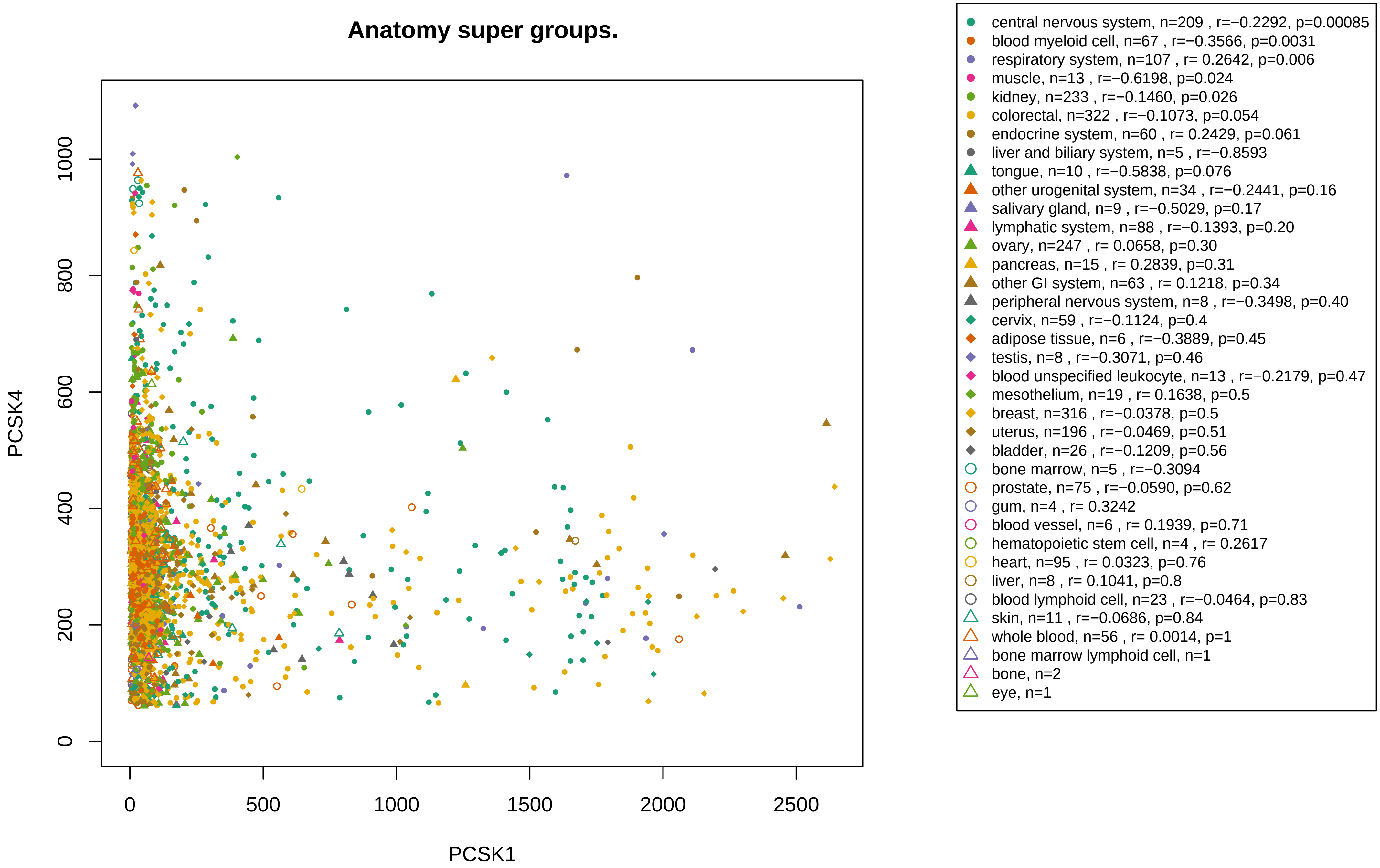
Anatomy super groups.
PCSK4
PCSK1
0
200
400
600
800
1000
0
500
1000
1500
2000
2500
central nervous system, n=209 , r=−0.2292, p=0.00085
blood myeloid cell, n=67 , r=−0.3566, p=0.0031
respiratory system, n=107 , r= 0.2642, p=0.006
muscle, n=13 , r=−0.6198, p=0.024
kidney, n=233 , r=−0.1460, p=0.026
colorectal, n=322 , r=−0.1073, p=0.054
endocrine system, n=60 , r= 0.2429, p=0.061
liver and biliary system, n=5 , r=−0.8593
tongue, n=10 , r=−0.5838, p=0.076
other urogenital system, n=34 , r=−0.2441, p=0.16
salivary gland, n=9 , r=−0.5029, p=0.17
lymphatic system, n=88 , r=−0.1393, p=0.20
ovary, n=247 , r= 0.0658, p=0.30
pancreas, n=15 , r= 0.2839, p=0.31
other GI system, n=63 , r= 0.1218, p=0.34
peripheral nervous system, n=8 , r=−0.3498, p=0.40
cervix, n=59 , r=−0.1124, p=0.4
adipose tissue, n=6 , r=−0.3889, p=0.45
testis, n=8 , r=−0.3071, p=0.46
blood unspecified leukocyte, n=13 , r=−0.2179, p=0.47
mesothelium, n=19 , r= 0.1638, p=0.5
breast, n=316 , r=−0.0378, p=0.5
uterus, n=196 , r=−0.0469, p=0.51
bladder, n=26 , r=−0.1209, p=0.56
bone marrow, n=5 , r=−0.3094
prostate, n=75 , r=−0.0590, p=0.62
gum, n=4 , r= 0.3242
blood vessel, n=6 , r= 0.1939, p=0.71
hematopoietic stem cell, n=4 , r= 0.2617
heart, n=95 , r= 0.0323, p=0.76
liver, n=8 , r= 0.1041, p=0.8
blood lymphoid cell, n=23 , r=−0.0464, p=0.83
skin, n=11 , r=−0.0686, p=0.84
whole blood, n=56 , r= 0.0014, p=1
bone marrow lymphoid cell, n=1
bone, n=2
eye, n=1

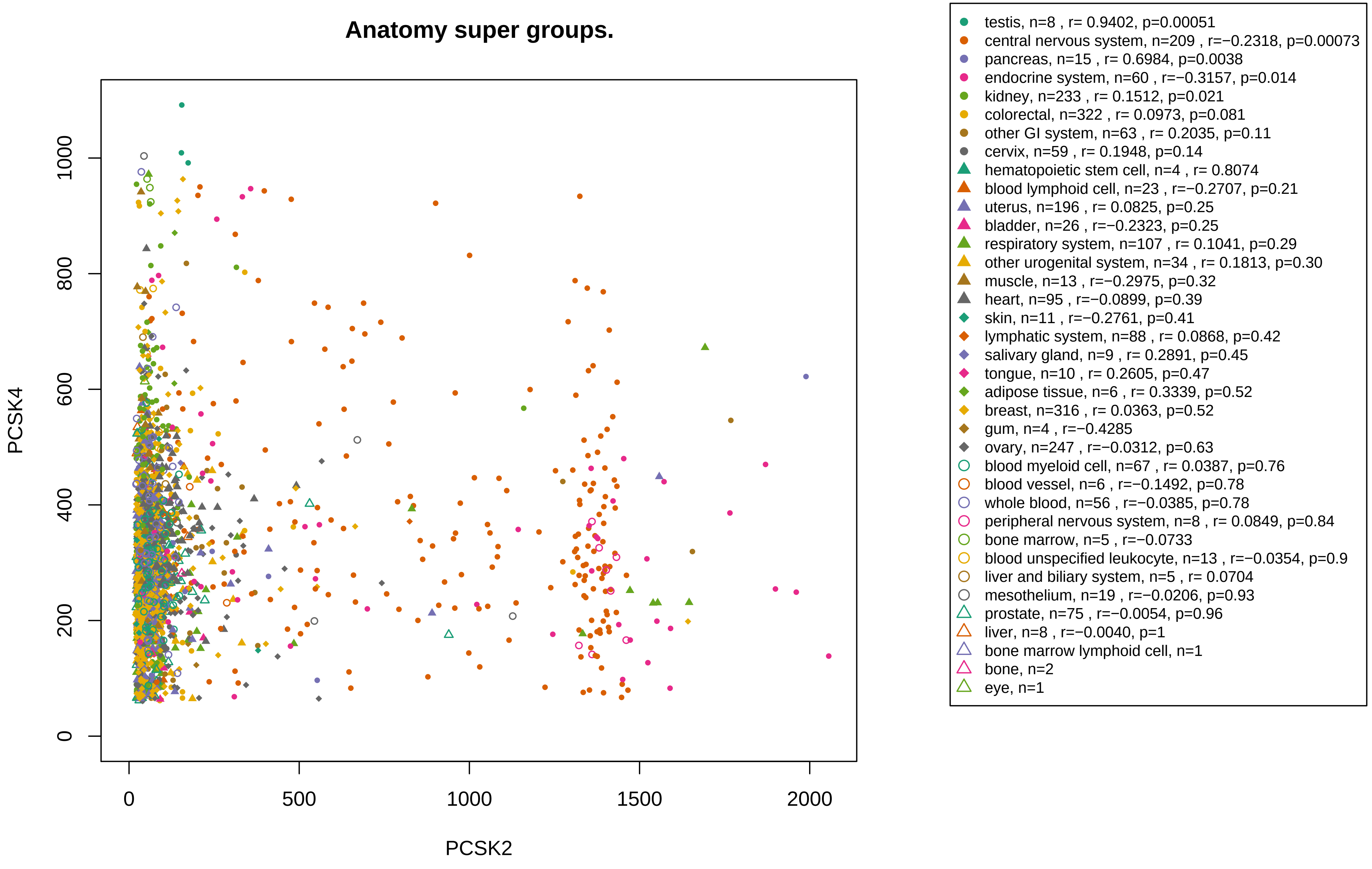

Anatomy super groups.
PCSK4
PCSK2
0
200
400
600
800
1000
0
500
1000
1500
2000
testis, n=8 , r= 0.9402, p=0.00051
central nervous system, n=209 , r=−0.2318, p=0.00073
pancreas, n=15 , r= 0.6984, p=0.0038
endocrine system, n=60 , r=−0.3157, p=0.014
kidney, n=233 , r= 0.1512, p=0.021
colorectal, n=322 , r= 0.0973, p=0.081
other GI system, n=63 , r= 0.2035, p=0.11
cervix, n=59 , r= 0.1948, p=0.14
hematopoietic stem cell, n=4 , r= 0.8074
blood lymphoid cell, n=23 , r=−0.2707, p=0.21
uterus, n=196 , r= 0.0825, p=0.25
bladder, n=26 , r=−0.2323, p=0.25
respiratory system, n=107 , r= 0.1041, p=0.29
other urogenital system, n=34 , r= 0.1813, p=0.30
muscle, n=13 , r=−0.2975, p=0.32
heart, n=95 , r=−0.0899, p=0.39
skin, n=11 , r=−0.2761, p=0.41
lymphatic system, n=88 , r= 0.0868, p=0.42
salivary gland, n=9 , r= 0.2891, p=0.45
tongue, n=10 , r= 0.2605, p=0.47
adipose tissue, n=6 , r= 0.3339, p=0.52
breast, n=316 , r= 0.0363, p=0.52
gum, n=4 , r=−0.4285
ovary, n=247 , r=−0.0312, p=0.63
blood myeloid cell, n=67 , r= 0.0387, p=0.76
blood vessel, n=6 , r=−0.1492, p=0.78
whole blood, n=56 , r=−0.0385, p=0.78
peripheral nervous system, n=8 , r= 0.0849, p=0.84
bone marrow, n=5 , r=−0.0733
blood unspecified leukocyte, n=13 , r=−0.0354, p=0.9
liver and biliary system, n=5 , r= 0.0704
mesothelium, n=19 , r=−0.0206, p=0.93
prostate, n=75 , r=−0.0054, p=0.96
liver, n=8 , r=−0.0040, p=1
bone marrow lymphoid cell, n=1
bone, n=2
eye, n=1

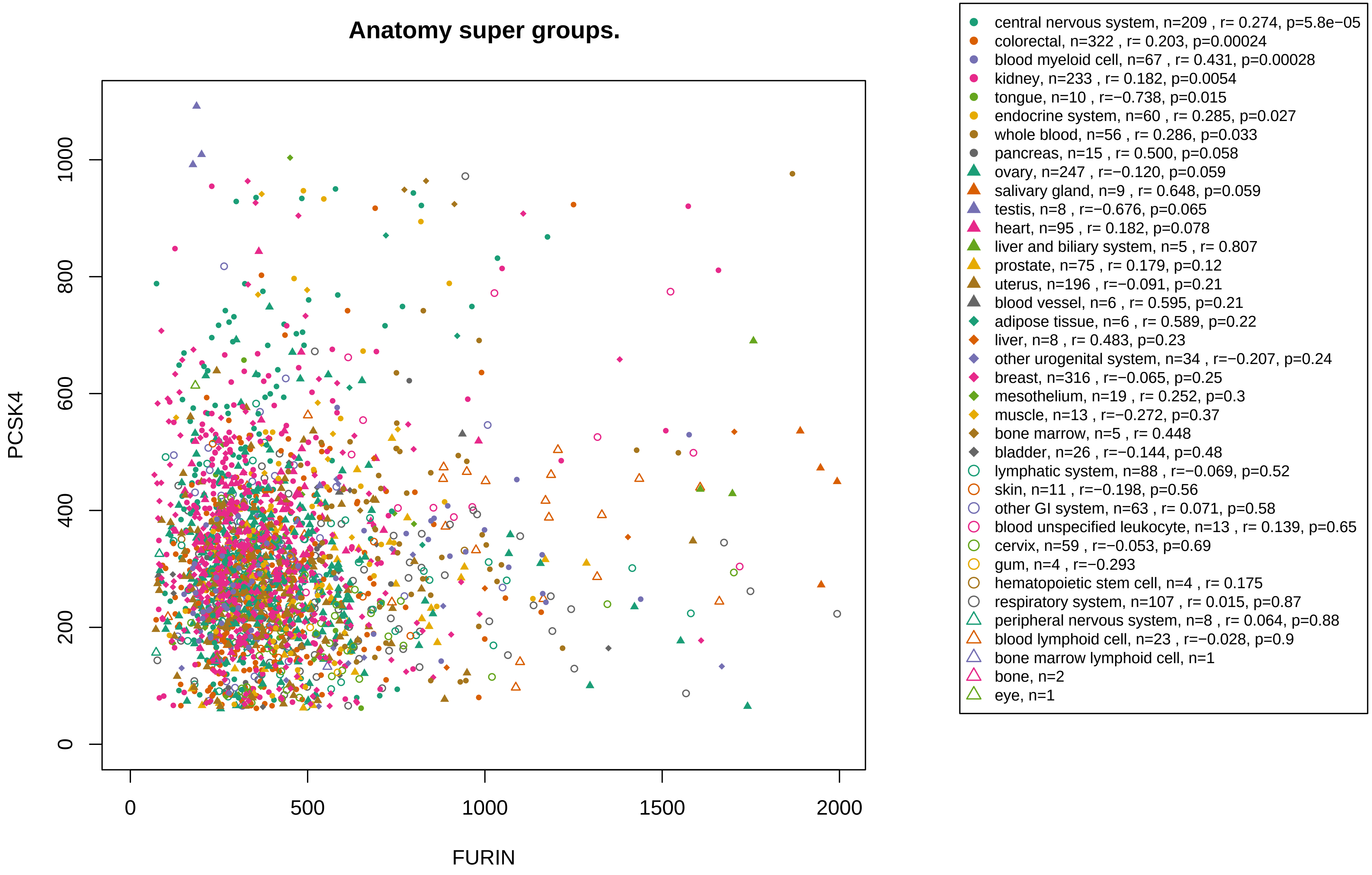
Anatomy super groups.
PCSK4
FURIN
0
200
400
600
800
1000
0
500
1000
1500
2000
central nervous system, n=209 , r= 0.274, p=5.8e−05
colorectal, n=322 , r= 0.203, p=0.00024
blood myeloid cell, n=67 , r= 0.431, p=0.00028
kidney, n=233 , r= 0.182, p=0.0054
tongue, n=10 , r=−0.738, p=0.015
endocrine system, n=60 , r= 0.285, p=0.027
whole blood, n=56 , r= 0.286, p=0.033
pancreas, n=15 , r= 0.500, p=0.058
ovary, n=247 , r=−0.120, p=0.059
salivary gland, n=9 , r= 0.648, p=0.059
testis, n=8 , r=−0.676, p=0.065
heart, n=95 , r= 0.182, p=0.078
liver and biliary system, n=5 , r= 0.807
prostate, n=75 , r= 0.179, p=0.12
uterus, n=196 , r=−0.091, p=0.21
blood vessel, n=6 , r= 0.595, p=0.21
adipose tissue, n=6 , r= 0.589, p=0.22
liver, n=8 , r= 0.483, p=0.23
other urogenital system, n=34 , r=−0.207, p=0.24
breast, n=316 , r=−0.065, p=0.25
mesothelium, n=19 , r= 0.252, p=0.3
muscle, n=13 , r=−0.272, p=0.37
bone marrow, n=5 , r= 0.448
bladder, n=26 , r=−0.144, p=0.48
lymphatic system, n=88 , r=−0.069, p=0.52
skin, n=11 , r=−0.198, p=0.56
other GI system, n=63 , r= 0.071, p=0.58
blood unspecified leukocyte, n=13 , r= 0.139, p=0.65
cervix, n=59 , r=−0.053, p=0.69
gum, n=4 , r=−0.293
hematopoietic stem cell, n=4 , r= 0.175
respiratory system, n=107 , r= 0.015, p=0.87
peripheral nervous system, n=8 , r= 0.064, p=0.88
blood lymphoid cell, n=23 , r=−0.028, p=0.9
bone marrow lymphoid cell, n=1
bone, n=2
eye, n=1

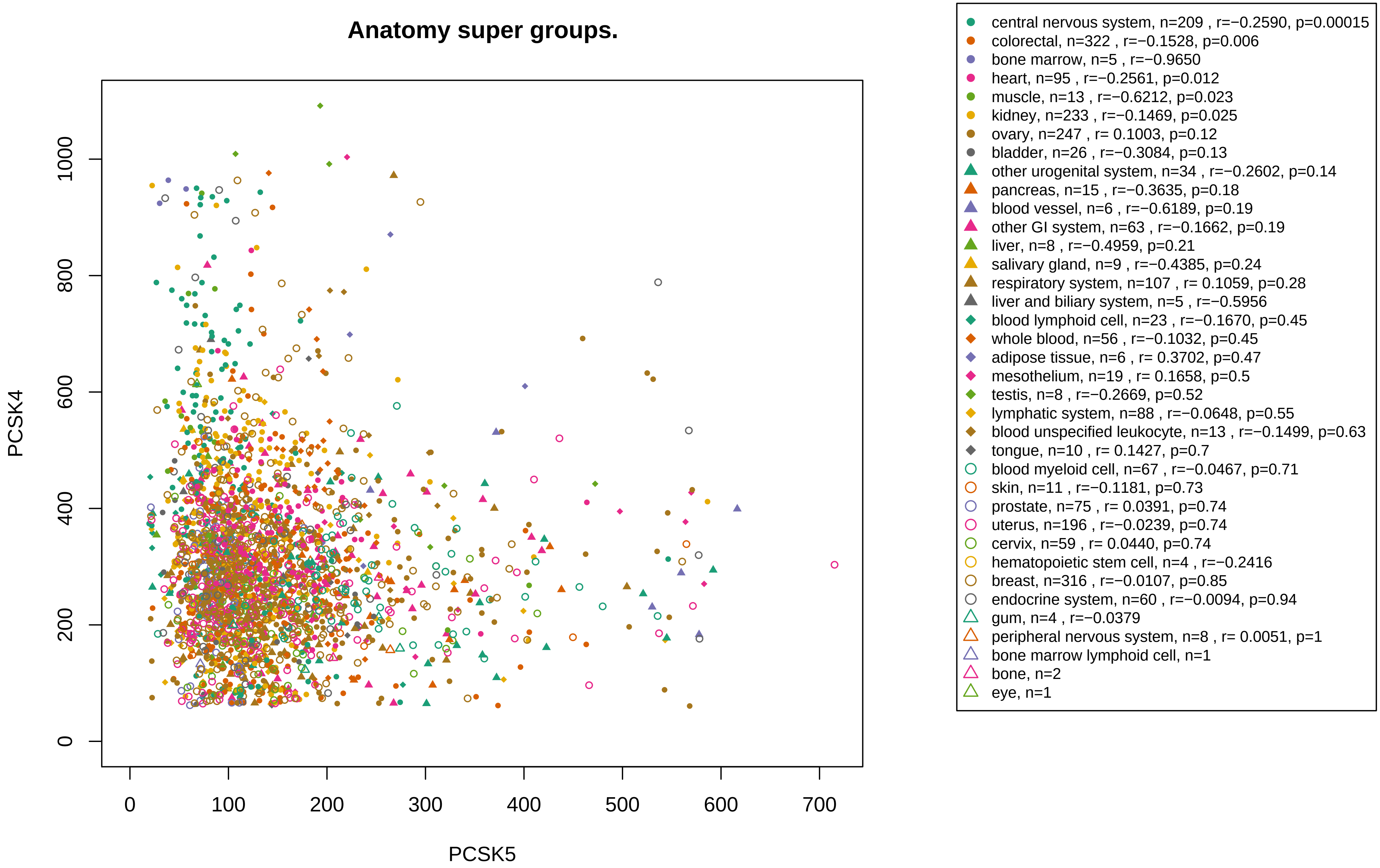
Anatomy super groups.
PCSK4
PCSK5
0
200
400
600
800
1000
0
100
200
300
400
500
600
700
central nervous system, n=209 , r=−0.2590, p=0.00015
colorectal, n=322 , r=−0.1528, p=0.006
bone marrow, n=5 , r=−0.9650
heart, n=95 , r=−0.2561, p=0.012
muscle, n=13 , r=−0.6212, p=0.023
kidney, n=233 , r=−0.1469, p=0.025
ovary, n=247 , r= 0.1003, p=0.12
bladder, n=26 , r=−0.3084, p=0.13
other urogenital system, n=34 , r=−0.2602, p=0.14
pancreas, n=15 , r=−0.3635, p=0.18
blood vessel, n=6 , r=−0.6189, p=0.19
other GI system, n=63 , r=−0.1662, p=0.19
liver, n=8 , r=−0.4959, p=0.21
salivary gland, n=9 , r=−0.4385, p=0.24
respiratory system, n=107 , r= 0.1059, p=0.28
liver and biliary system, n=5 , r=−0.5956
blood lymphoid cell, n=23 , r=−0.1670, p=0.45
whole blood, n=56 , r=−0.1032, p=0.45
adipose tissue, n=6 , r= 0.3702, p=0.47
mesothelium, n=19 , r= 0.1658, p=0.5
testis, n=8 , r=−0.2669, p=0.52
lymphatic system, n=88 , r=−0.0648, p=0.55
blood unspecified leukocyte, n=13 , r=−0.1499, p=0.63
tongue, n=10 , r= 0.1427, p=0.7
blood myeloid cell, n=67 , r=−0.0467, p=0.71
skin, n=11 , r=−0.1181, p=0.73
prostate, n=75 , r= 0.0391, p=0.74
uterus, n=196 , r=−0.0239, p=0.74
cervix, n=59 , r= 0.0440, p=0.74
hematopoietic stem cell, n=4 , r=−0.2416
breast, n=316 , r=−0.0107, p=0.85
endocrine system, n=60 , r=−0.0094, p=0.94
gum, n=4 , r=−0.0379
peripheral nervous system, n=8 , r= 0.0051, p=1
bone marrow lymphoid cell, n=1
bone, n=2
eye, n=1

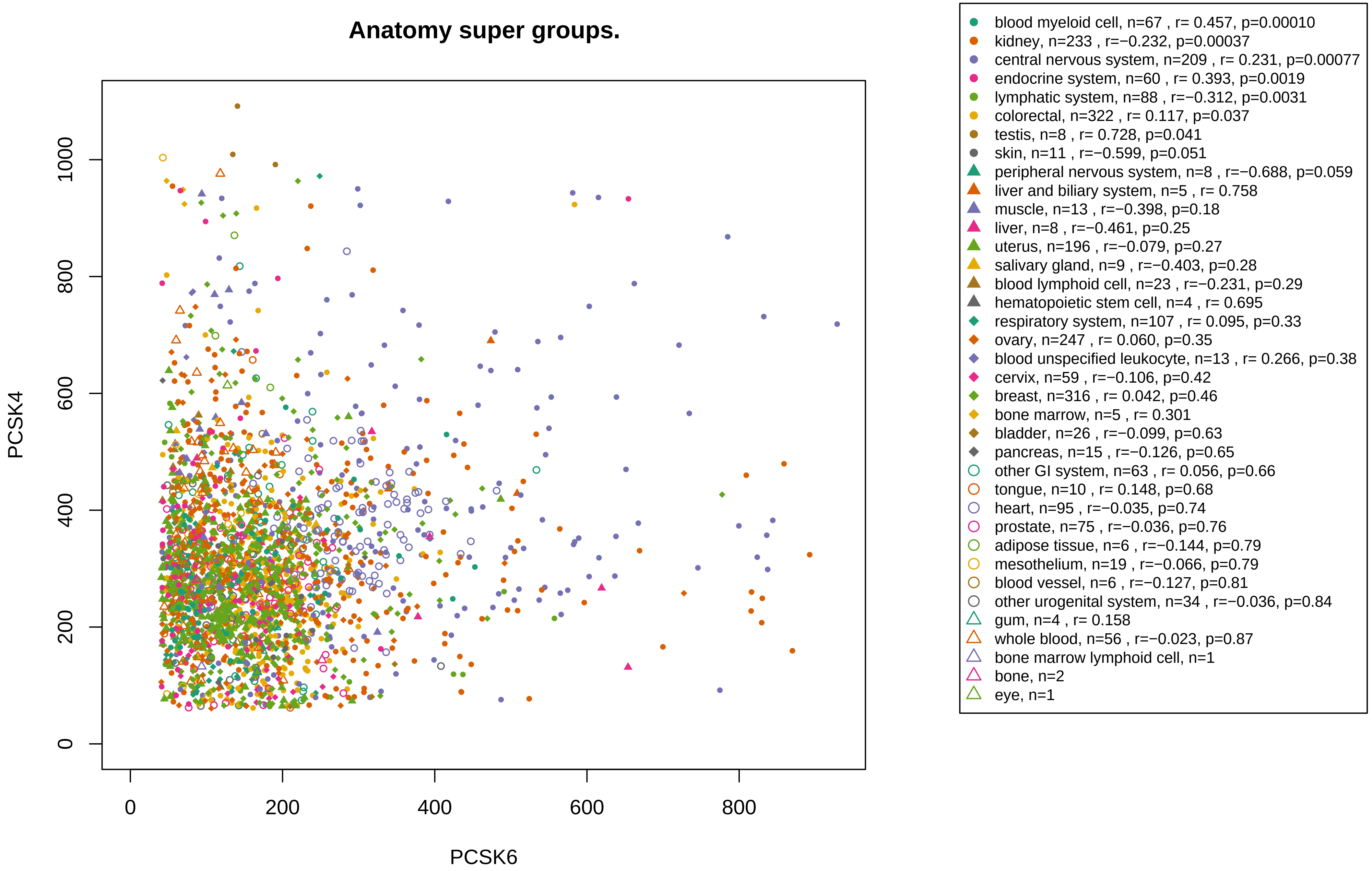
Anatomy super groups.
PCSK4
PCSK6
0
200
400
600
800
1000
blood myeloid cell, n=67 , r= 0.457, p=0.00010
kidney, n=233 , r=−0.232, p=0.00037
central nervous system, n=209 , r= 0.231, p=0.00077
endocrine system, n=60 , r= 0.393, p=0.0019
lymphatic system, n=88 , r=−0.312, p=0.0031
colorectal, n=322 , r= 0.117, p=0.037
testis, n=8 , r= 0.728, p=0.041
skin, n=11 , r=−0.599, p=0.051
peripheral nervous system, n=8 , r=−0.688, p=0.059
liver and biliary system, n=5 , r= 0.758
muscle, n=13 , r=−0.398, p=0.18
liver, n=8 , r=−0.461, p=0.25
uterus, n=196 , r=−0.079, p=0.27
salivary gland, n=9 , r=−0.403, p=0.28
blood lymphoid cell, n=23 , r=−0.231, p=0.29
hematopoietic stem cell, n=4 , r= 0.695
respiratory system, n=107 , r= 0.095, p=0.33
ovary, n=247 , r= 0.060, p=0.35
blood unspecified leukocyte, n=13 , r= 0.266, p=0.38
cervix, n=59 , r=−0.106, p=0.42
breast, n=316 , r= 0.042, p=0.46
bone marrow, n=5 , r= 0.301
bladder, n=26 , r=−0.099, p=0.63
pancreas, n=15 , r=−0.126, p=0.65
other GI system, n=63 , r= 0.056, p=0.66
tongue, n=10 , r= 0.148, p=0.68
heart, n=95 , r=−0.035, p=0.74
prostate, n=75 , r=−0.036, p=0.76
adipose tissue, n=6 , r=−0.144, p=0.79
mesothelium, n=19 , r=−0.066, p=0.79
blood vessel, n=6 , r=−0.127, p=0.81
other urogenital system, n=34 , r=−0.036, p=0.84
gum, n=4 , r= 0.158
whole blood, n=56 , r=−0.023, p=0.87
bone marrow lymphoid cell, n=1
bone, n=2
eye, n=1

Anatomy super groups.

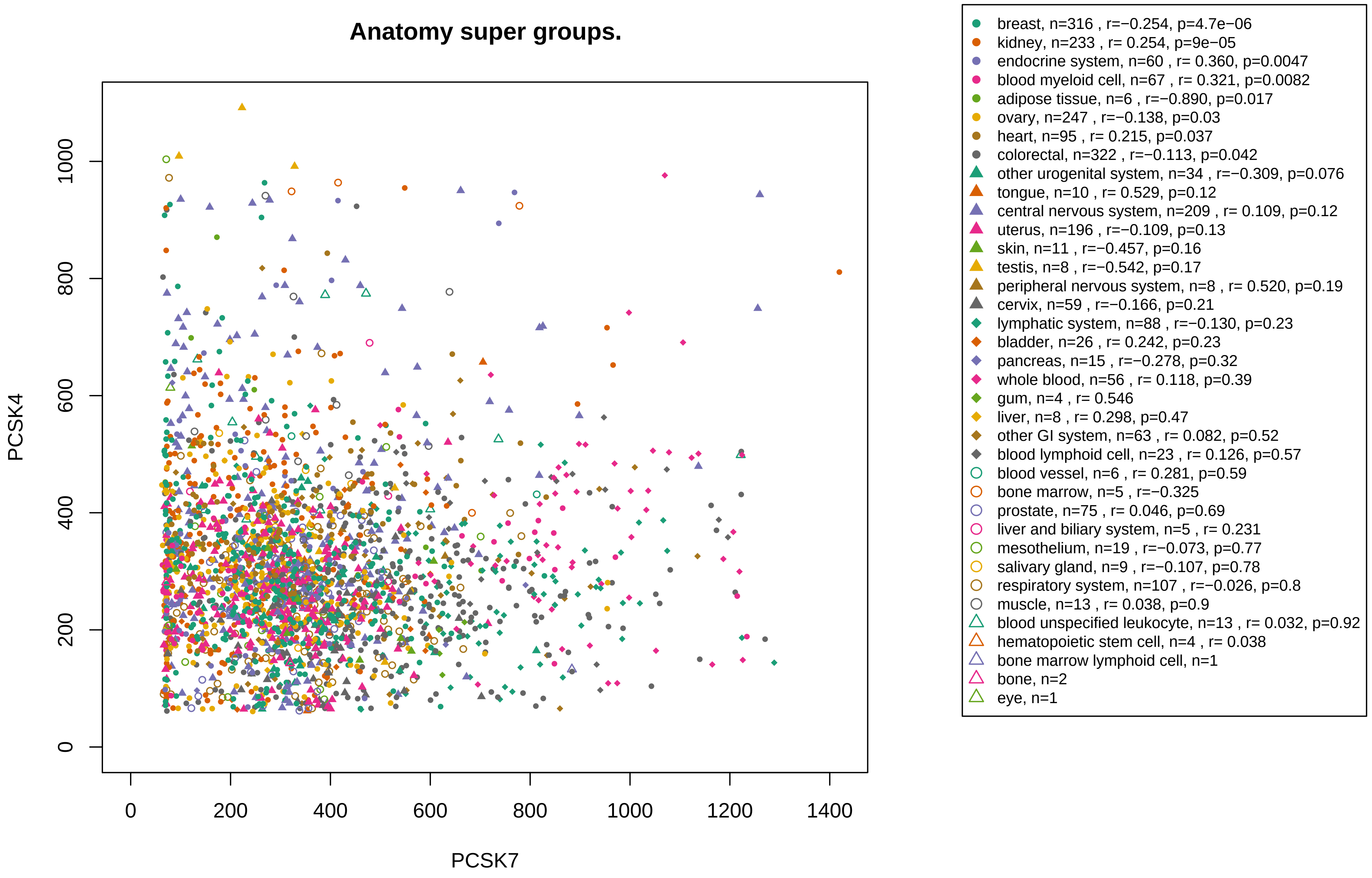

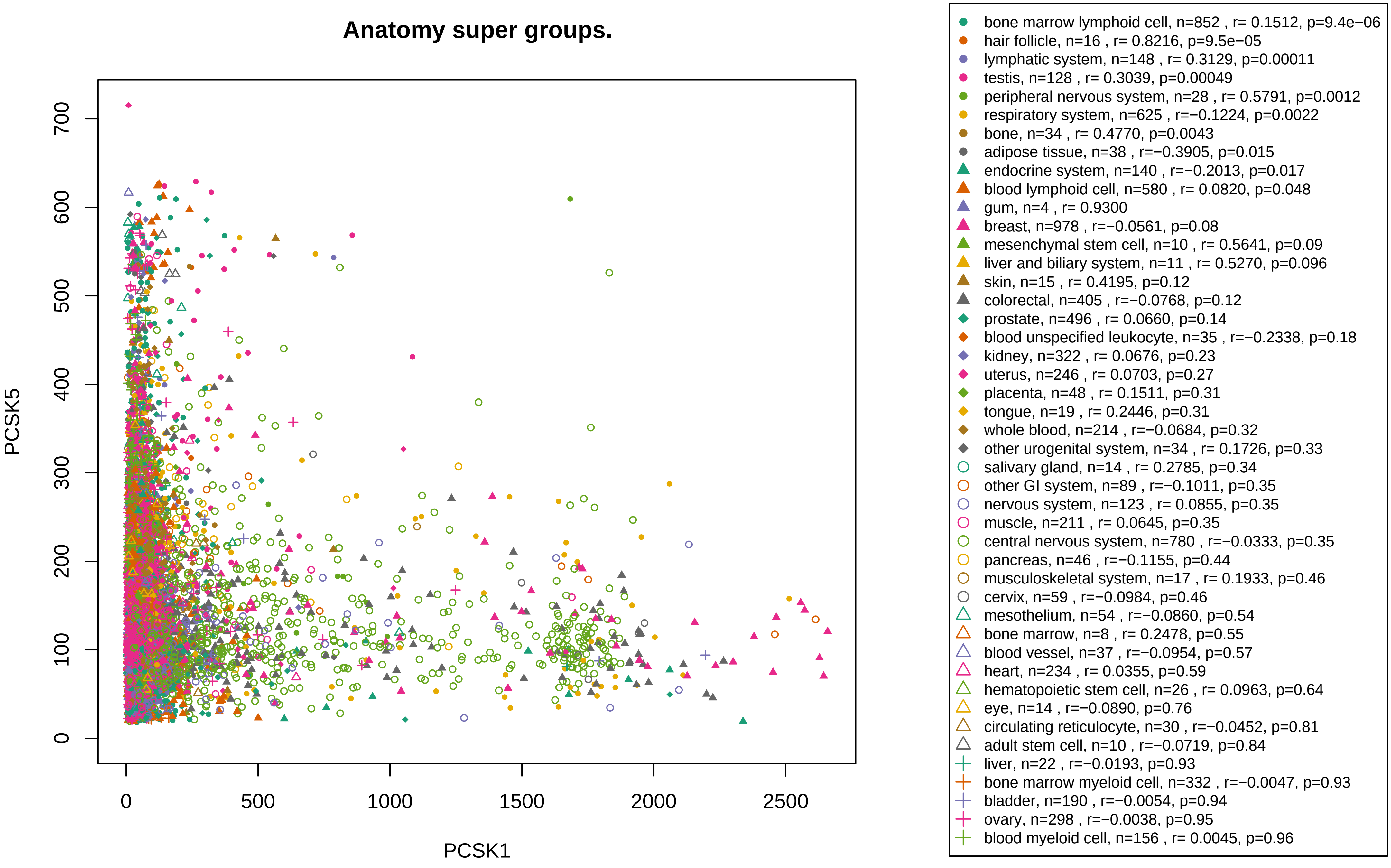
Anatomy super groups.
PCSK5
PCSK1
0
100
200
300
400
500
600
700
0
500
1000
1500
2000
2500
bone marrow lymphoid cell, n=852 , r= 0.1512, p=9.4e−06
hair follicle, n=16 , r= 0.8216, p=9.5e−05
lymphatic system, n=148 , r= 0.3129, p=0.00011
testis, n=128 , r= 0.3039, p=0.00049
peripheral nervous system, n=28 , r= 0.5791, p=0.0012
respiratory system, n=625 , r=−0.1224, p=0.0022
bone, n=34 , r= 0.4770, p=0.0043
adipose tissue, n=38 , r=−0.3905, p=0.015
endocrine system, n=140 , r=−0.2013, p=0.017
blood lymphoid cell, n=580 , r= 0.0820, p=0.048
gum, n=4 , r= 0.9300
breast, n=978 , r=−0.0561, p=0.08
mesenchymal stem cell, n=10 , r= 0.5641, p=0.09
liver and biliary system, n=11 , r= 0.5270, p=0.096
skin, n=15 , r= 0.4195, p=0.12
colorectal, n=405 , r=−0.0768, p=0.12
prostate, n=496 , r= 0.0660, p=0.14
blood unspecified leukocyte, n=35 , r=−0.2338, p=0.18
kidney, n=322 , r= 0.0676, p=0.23
uterus, n=246 , r= 0.0703, p=0.27
placenta, n=48 , r= 0.1511, p=0.31
tongue, n=19 , r= 0.2446, p=0.31
whole blood, n=214 , r=−0.0684, p=0.32
other urogenital system, n=34 , r= 0.1726, p=0.33
salivary gland, n=14 , r= 0.2785, p=0.34
other GI system, n=89 , r=−0.1011, p=0.35
nervous system, n=123 , r= 0.0855, p=0.35
muscle, n=211 , r= 0.0645, p=0.35
central nervous system, n=780 , r=−0.0333, p=0.35
pancreas, n=46 , r=−0.1155, p=0.44
musculoskeletal system, n=17 , r= 0.1933, p=0.46
cervix, n=59 , r=−0.0984, p=0.46
mesothelium, n=54 , r=−0.0860, p=0.54
bone marrow, n=8 , r= 0.2478, p=0.55
blood vessel, n=37 , r=−0.0954, p=0.57
heart, n=234 , r= 0.0355, p=0.59
hematopoietic stem cell, n=26 , r= 0.0963, p=0.64
eye, n=14 , r=−0.0890, p=0.76
circulating reticulocyte, n=30 , r=−0.0452, p=0.81
adult stem cell, n=10 , r=−0.0719, p=0.84
liver, n=22 , r=−0.0193, p=0.93
bone marrow myeloid cell, n=332 , r=−0.0047, p=0.93
bladder, n=190 , r=−0.0054, p=0.94
ovary, n=298 , r=−0.0038, p=0.95
blood myeloid cell, n=156 , r= 0.0045, p=0.96

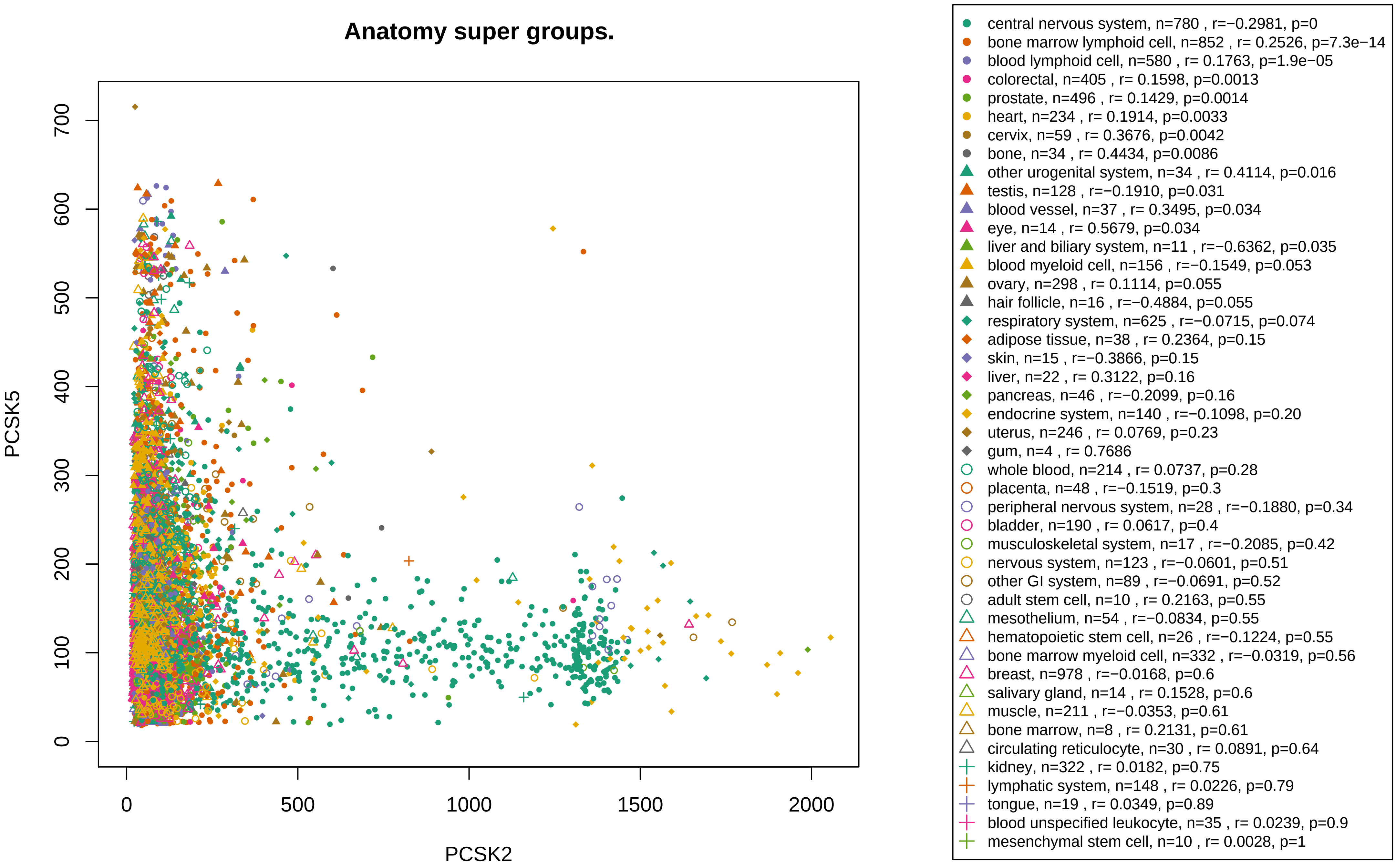
Anatomy super groups.
PCSK5
PCSK2
0
100
200
300
400
500
600
700
0
500
1000
1500
2000
central nervous system, n=780 , r=−0.2981, p=0
bone marrow lymphoid cell, n=852 , r= 0.2526, p=7.3e−14
blood lymphoid cell, n=580 , r= 0.1763, p=1.9e−05
colorectal, n=405 , r= 0.1598, p=0.0013
prostate, n=496 , r= 0.1429, p=0.0014
heart, n=234 , r= 0.1914, p=0.0033
cervix, n=59 , r= 0.3676, p=0.0042
bone, n=34 , r= 0.4434, p=0.0086
other urogenital system, n=34 , r= 0.4114, p=0.016
testis, n=128 , r=−0.1910, p=0.031
blood vessel, n=37 , r= 0.3495, p=0.034
eye, n=14 , r= 0.5679, p=0.034
liver and biliary system, n=11 , r=−0.6362, p=0.035
blood myeloid cell, n=156 , r=−0.1549, p=0.053
ovary, n=298 , r= 0.1114, p=0.055
hair follicle, n=16 , r=−0.4884, p=0.055
respiratory system, n=625 , r=−0.0715, p=0.074
adipose tissue, n=38 , r= 0.2364, p=0.15
skin, n=15 , r=−0.3866, p=0.15
liver, n=22 , r= 0.3122, p=0.16
pancreas, n=46 , r=−0.2099, p=0.16
endocrine system, n=140 , r=−0.1098, p=0.20
uterus, n=246 , r= 0.0769, p=0.23
gum, n=4 , r= 0.7686
whole blood, n=214 , r= 0.0737, p=0.28
placenta, n=48 , r=−0.1519, p=0.3
peripheral nervous system, n=28 , r=−0.1880, p=0.34
bladder, n=190 , r= 0.0617, p=0.4
musculoskeletal system, n=17 , r=−0.2085, p=0.42
nervous system, n=123 , r=−0.0601, p=0.51
other GI system, n=89 , r=−0.0691, p=0.52
adult stem cell, n=10 , r= 0.2163, p=0.55
mesothelium, n=54 , r=−0.0834, p=0.55
hematopoietic stem cell, n=26 , r=−0.1224, p=0.55
bone marrow myeloid cell, n=332 , r=−0.0319, p=0.56
breast, n=978 , r=−0.0168, p=0.6
salivary gland, n=14 , r= 0.1528, p=0.6
muscle, n=211 , r=−0.0353, p=0.61
bone marrow, n=8 , r= 0.2131, p=0.61
circulating reticulocyte, n=30 , r= 0.0891, p=0.64
kidney, n=322 , r= 0.0182, p=0.75
lymphatic system, n=148 , r= 0.0226, p=0.79
tongue, n=19 , r= 0.0349, p=0.89
blood unspecified leukocyte, n=35 , r= 0.0239, p=0.9
mesenchymal stem cell, n=10 , r= 0.0028, p=1

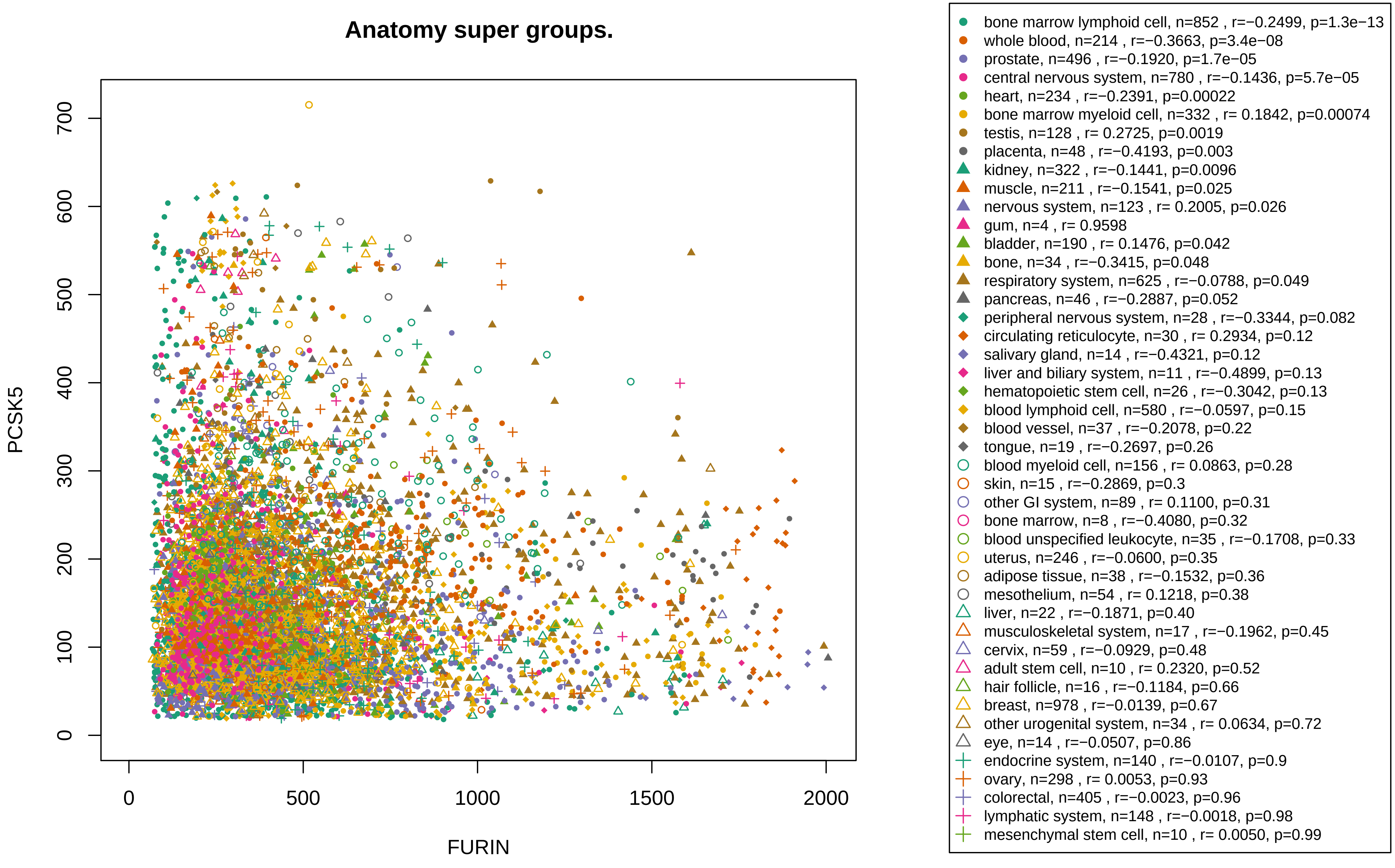

Anatomy super groups.
PCSK5
FURIN
0
100
200
300
400
500
600
700
0
500
1000
1500
2000
bone marrow lymphoid cell, n=852 , r=−0.2499, p=1.3e−13
whole blood, n=214 , r=−0.3663, p=3.4e−08
prostate, n=496 , r=−0.1920, p=1.7e−05
central nervous system, n=780 , r=−0.1436, p=5.7e−05
heart, n=234 , r=−0.2391, p=0.00022
bone marrow myeloid cell, n=332 , r= 0.1842, p=0.00074
testis, n=128 , r= 0.2725, p=0.0019
placenta, n=48 , r=−0.4193, p=0.003
kidney, n=322 , r=−0.1441, p=0.0096
muscle, n=211 , r=−0.1541, p=0.025
nervous system, n=123 , r= 0.2005, p=0.026
gum, n=4 , r= 0.9598
bladder, n=190 , r= 0.1476, p=0.042
bone, n=34 , r=−0.3415, p=0.048
respiratory system, n=625 , r=−0.0788, p=0.049
pancreas, n=46 , r=−0.2887, p=0.052
peripheral nervous system, n=28 , r=−0.3344, p=0.082
circulating reticulocyte, n=30 , r= 0.2934, p=0.12
salivary gland, n=14 , r=−0.4321, p=0.12
liver and biliary system, n=11 , r=−0.4899, p=0.13
hematopoietic stem cell, n=26 , r=−0.3042, p=0.13
blood lymphoid cell, n=580 , r=−0.0597, p=0.15
blood vessel, n=37 , r=−0.2078, p=0.22
tongue, n=19 , r=−0.2697, p=0.26
blood myeloid cell, n=156 , r= 0.0863, p=0.28
skin, n=15 , r=−0.2869, p=0.3
other GI system, n=89 , r= 0.1100, p=0.31
bone marrow, n=8 , r=−0.4080, p=0.32
blood unspecified leukocyte, n=35 , r=−0.1708, p=0.33
uterus, n=246 , r=−0.0600, p=0.35
adipose tissue, n=38 , r=−0.1532, p=0.36
mesothelium, n=54 , r= 0.1218, p=0.38
liver, n=22 , r=−0.1871, p=0.40
musculoskeletal system, n=17 , r=−0.1962, p=0.45
cervix, n=59 , r=−0.0929, p=0.48
adult stem cell, n=10 , r= 0.2320, p=0.52
hair follicle, n=16 , r=−0.1184, p=0.66
breast, n=978 , r=−0.0139, p=0.67
other urogenital system, n=34 , r= 0.0634, p=0.72
eye, n=14 , r=−0.0507, p=0.86
endocrine system, n=140 , r=−0.0107, p=0.9
ovary, n=298 , r= 0.0053, p=0.93
colorectal, n=405 , r=−0.0023, p=0.96
lymphatic system, n=148 , r=−0.0018, p=0.98
mesenchymal stem cell, n=10 , r= 0.0050, p=0.99

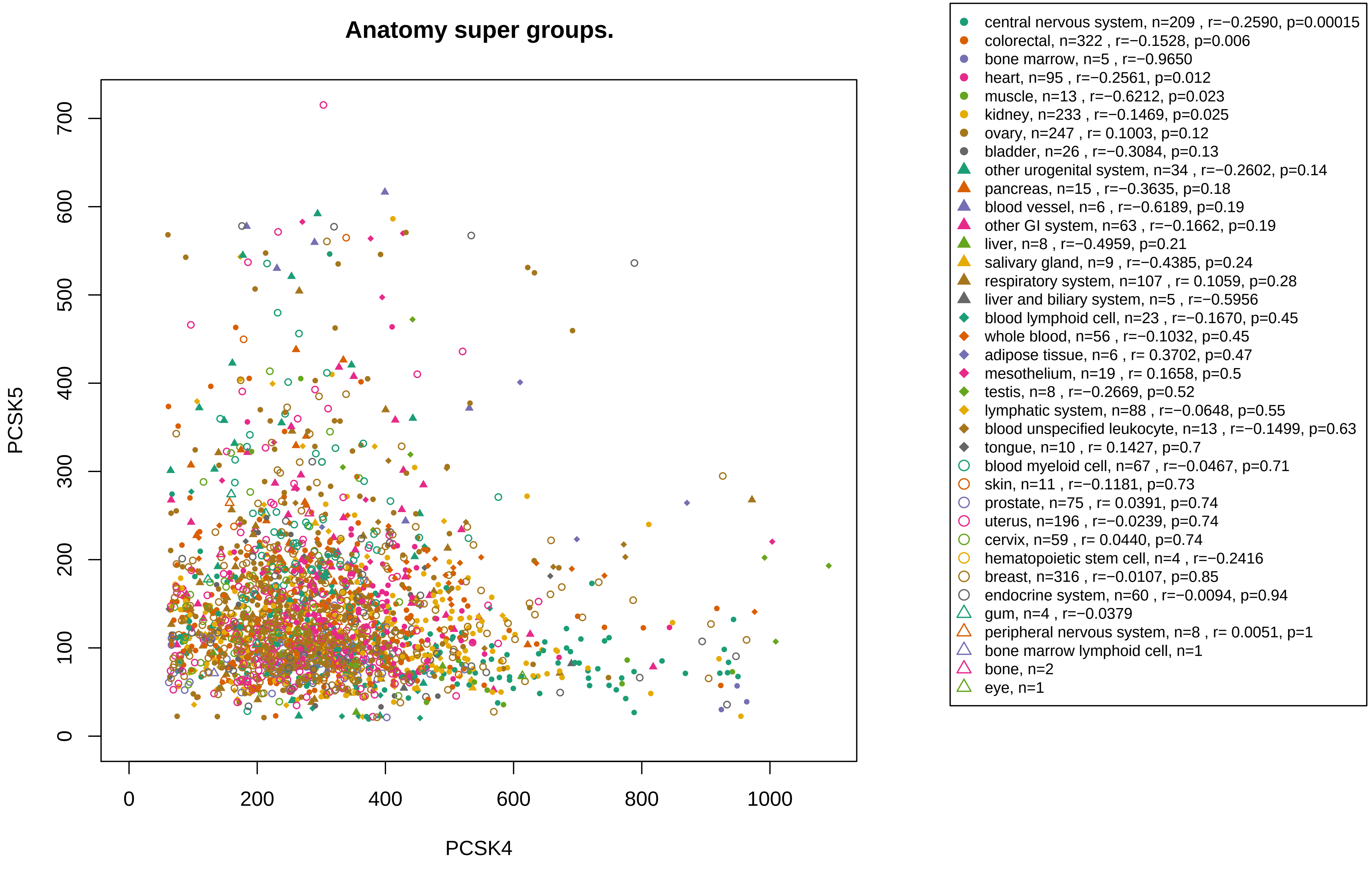
Anatomy super groups.
PCSK5
PCSK4
0
100
200
300
400
500
600
700
0
200
400
600
800
1000
central nervous system, n=209 , r=−0.2590, p=0.00015
colorectal, n=322 , r=−0.1528, p=0.006
bone marrow, n=5 , r=−0.9650
heart, n=95 , r=−0.2561, p=0.012
muscle, n=13 , r=−0.6212, p=0.023
kidney, n=233 , r=−0.1469, p=0.025
ovary, n=247 , r= 0.1003, p=0.12
bladder, n=26 , r=−0.3084, p=0.13
other urogenital system, n=34 , r=−0.2602, p=0.14
pancreas, n=15 , r=−0.3635, p=0.18
blood vessel, n=6 , r=−0.6189, p=0.19
other GI system, n=63 , r=−0.1662, p=0.19
liver, n=8 , r=−0.4959, p=0.21
salivary gland, n=9 , r=−0.4385, p=0.24
respiratory system, n=107 , r= 0.1059, p=0.28
liver and biliary system, n=5 , r=−0.5956
blood lymphoid cell, n=23 , r=−0.1670, p=0.45
whole blood, n=56 , r=−0.1032, p=0.45
adipose tissue, n=6 , r= 0.3702, p=0.47
mesothelium, n=19 , r= 0.1658, p=0.5
testis, n=8 , r=−0.2669, p=0.52
lymphatic system, n=88 , r=−0.0648, p=0.55
blood unspecified leukocyte, n=13 , r=−0.1499, p=0.63
tongue, n=10 , r= 0.1427, p=0.7
blood myeloid cell, n=67 , r=−0.0467, p=0.71
skin, n=11 , r=−0.1181, p=0.73
prostate, n=75 , r= 0.0391, p=0.74
uterus, n=196 , r=−0.0239, p=0.74
cervix, n=59 , r= 0.0440, p=0.74
hematopoietic stem cell, n=4 , r=−0.2416
breast, n=316 , r=−0.0107, p=0.85
endocrine system, n=60 , r=−0.0094, p=0.94
gum, n=4 , r=−0.0379
peripheral nervous system, n=8 , r= 0.0051, p=1
bone marrow lymphoid cell, n=1
bone, n=2
eye, n=1

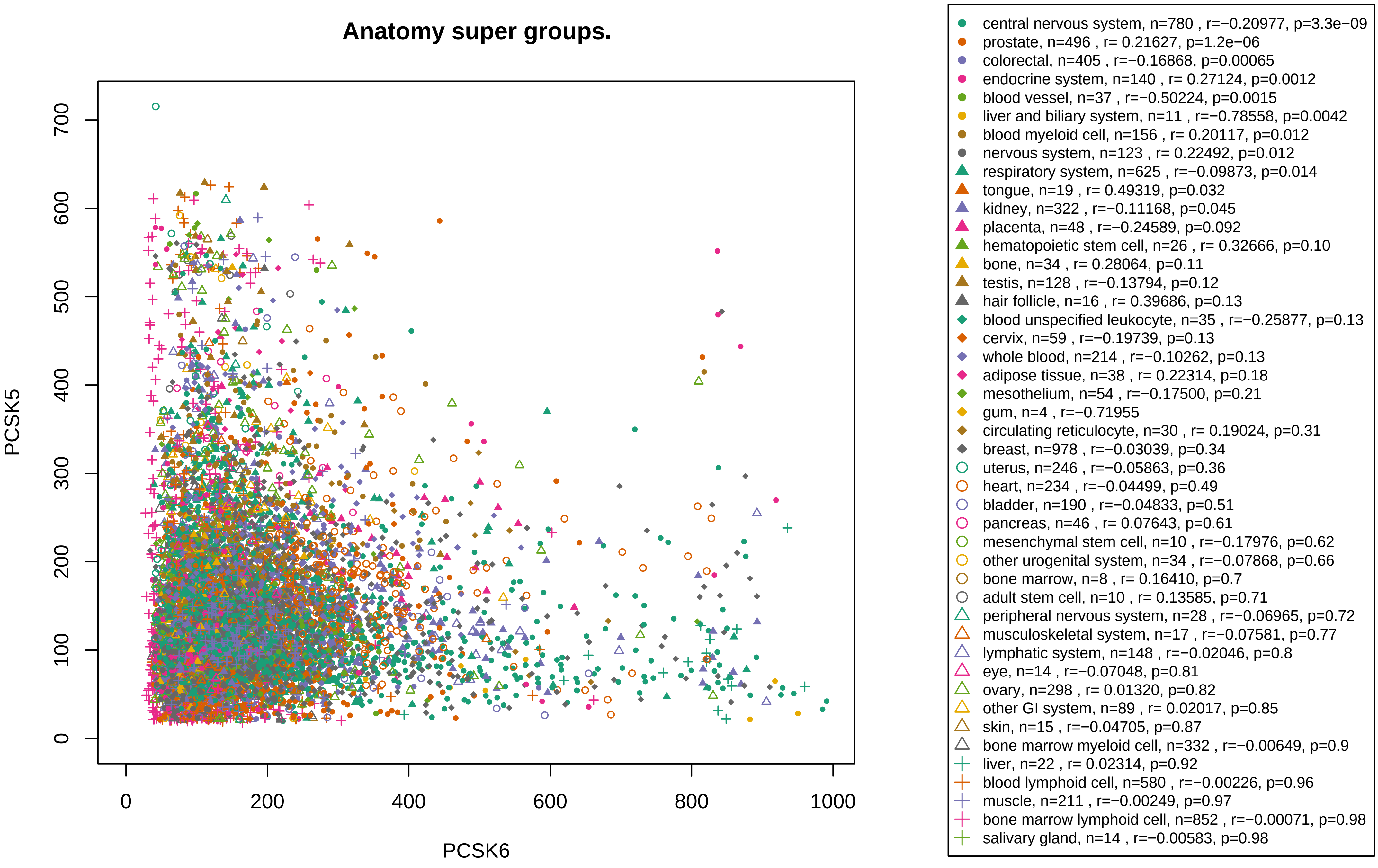
Anatomy super groups.
PCSK5
PCSK6
0
100
200
300
400
500
600
700
0
200
400
600
800
1000
central nervous system, n=780 , r=−0.20977, p=3.3e−09
prostate, n=496 , r= 0.21627, p=1.2e−06
colorectal, n=405 , r=−0.16868, p=0.00065
endocrine system, n=140 , r= 0.27124, p=0.0012
blood vessel, n=37 , r=−0.50224, p=0.0015
liver and biliary system, n=11 , r=−0.78558, p=0.0042
blood myeloid cell, n=156 , r= 0.20117, p=0.012
nervous system, n=123 , r= 0.22492, p=0.012
respiratory system, n=625 , r=−0.09873, p=0.014
tongue, n=19 , r= 0.49319, p=0.032
kidney, n=322 , r=−0.11168, p=0.045
placenta, n=48 , r=−0.24589, p=0.092
hematopoietic stem cell, n=26 , r= 0.32666, p=0.10
bone, n=34 , r= 0.28064, p=0.11
testis, n=128 , r=−0.13794, p=0.12
hair follicle, n=16 , r= 0.39686, p=0.13
blood unspecified leukocyte, n=35 , r=−0.25877, p=0.13
cervix, n=59 , r=−0.19739, p=0.13
whole blood, n=214 , r=−0.10262, p=0.13
adipose tissue, n=38 , r= 0.22314, p=0.18
mesothelium, n=54 , r=−0.17500, p=0.21
gum, n=4 , r=−0.71955
circulating reticulocyte, n=30 , r= 0.19024, p=0.31
breast, n=978 , r=−0.03039, p=0.34
uterus, n=246 , r=−0.05863, p=0.36
heart, n=234 , r=−0.04499, p=0.49
bladder, n=190 , r=−0.04833, p=0.51
pancreas, n=46 , r= 0.07643, p=0.61
mesenchymal stem cell, n=10 , r=−0.17976, p=0.62
other urogenital system, n=34 , r=−0.07868, p=0.66
bone marrow, n=8 , r= 0.16410, p=0.7
adult stem cell, n=10 , r= 0.13585, p=0.71
peripheral nervous system, n=28 , r=−0.06965, p=0.72
musculoskeletal system, n=17 , r=−0.07581, p=0.77
lymphatic system, n=148 , r=−0.02046, p=0.8
eye, n=14 , r=−0.07048, p=0.81
ovary, n=298 , r= 0.01320, p=0.82
other GI system, n=89 , r= 0.02017, p=0.85
skin, n=15 , r=−0.04705, p=0.87
bone marrow myeloid cell, n=332 , r=−0.00649, p=0.9
liver, n=22 , r= 0.02314, p=0.92
blood lymphoid cell, n=580 , r=−0.00226, p=0.96
muscle, n=211 , r=−0.00249, p=0.97
bone marrow lymphoid cell, n=852 , r=−0.00071, p=0.98
salivary gland, n=14 , r=−0.00583, p=0.98

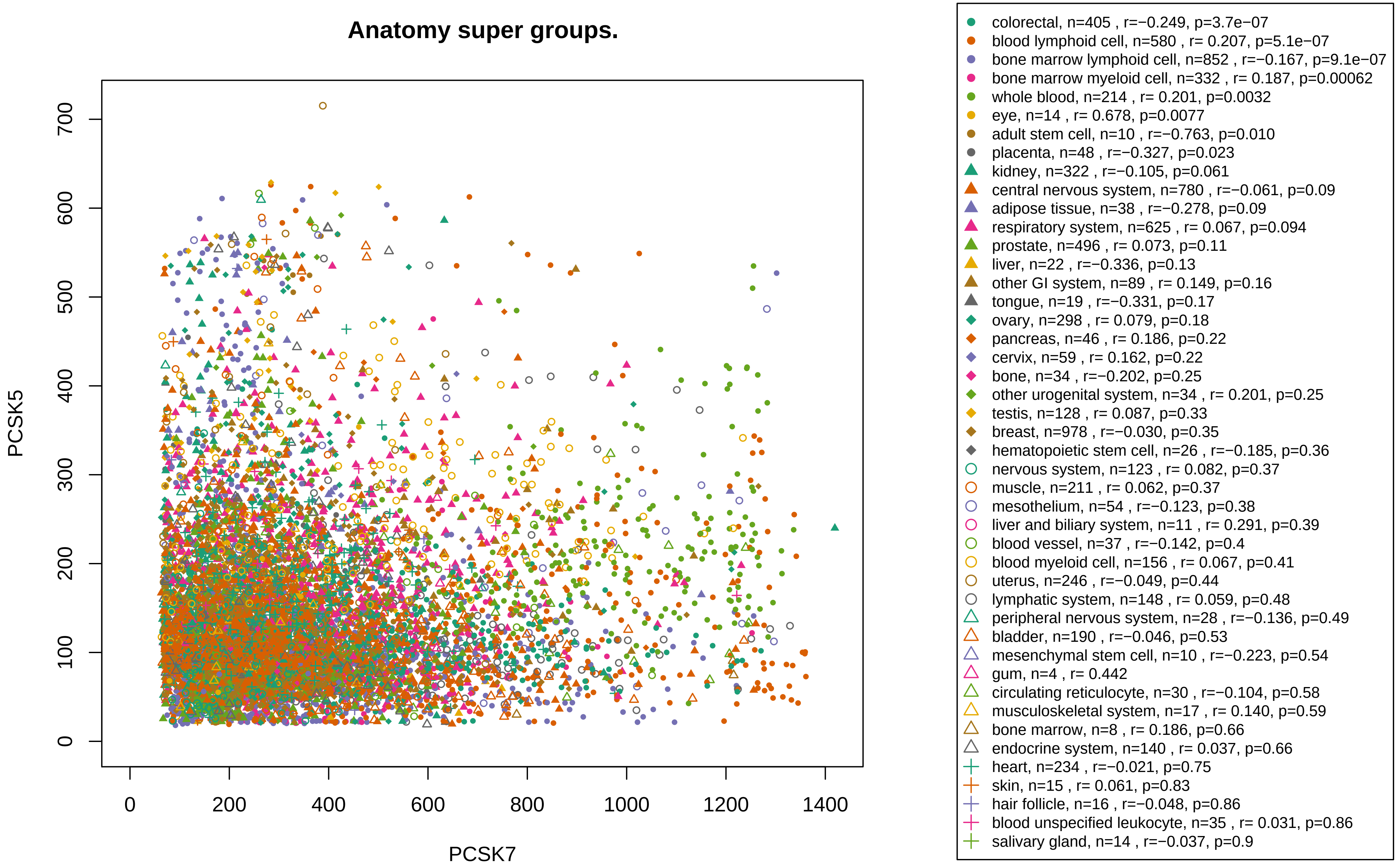

Anatomy super groups.
PCSK5
PCSK7
0
100
200
300
400
500
600
700
0
200
400
600
800
1000
1200
1400
colorectal, n=405 , r=−0.249, p=3.7e−07
blood lymphoid cell, n=580 , r= 0.207, p=5.1e−07
bone marrow lymphoid cell, n=852 , r=−0.167, p=9.1e−07
bone marrow myeloid cell, n=332 , r= 0.187, p=0.00062
whole blood, n=214 , r= 0.201, p=0.0032
eye, n=14 , r= 0.678, p=0.0077
adult stem cell, n=10 , r=−0.763, p=0.010
placenta, n=48 , r=−0.327, p=0.023
kidney, n=322 , r=−0.105, p=0.061
central nervous system, n=780 , r=−0.061, p=0.09
adipose tissue, n=38 , r=−0.278, p=0.09
respiratory system, n=625 , r= 0.067, p=0.094
prostate, n=496 , r= 0.073, p=0.11
liver, n=22 , r=−0.336, p=0.13
other GI system, n=89 , r= 0.149, p=0.16
tongue, n=19 , r=−0.331, p=0.17
ovary, n=298 , r= 0.079, p=0.18
pancreas, n=46 , r= 0.186, p=0.22
cervix, n=59 , r= 0.162, p=0.22
bone, n=34 , r=−0.202, p=0.25
other urogenital system, n=34 , r= 0.201, p=0.25
testis, n=128 , r= 0.087, p=0.33
breast, n=978 , r=−0.030, p=0.35
hematopoietic stem cell, n=26 , r=−0.185, p=0.36
nervous system, n=123 , r= 0.082, p=0.37
muscle, n=211 , r= 0.062, p=0.37
mesothelium, n=54 , r=−0.123, p=0.38
liver and biliary system, n=11 , r= 0.291, p=0.39
blood vessel, n=37 , r=−0.142, p=0.4
blood myeloid cell, n=156 , r= 0.067, p=0.41
uterus, n=246 , r=−0.049, p=0.44
lymphatic system, n=148 , r= 0.059, p=0.48
peripheral nervous system, n=28 , r=−0.136, p=0.49
bladder, n=190 , r=−0.046, p=0.53
mesenchymal stem cell, n=10 , r=−0.223, p=0.54
gum, n=4 , r= 0.442
circulating reticulocyte, n=30 , r=−0.104, p=0.58
musculoskeletal system, n=17 , r= 0.140, p=0.59
bone marrow, n=8 , r= 0.186, p=0.66
endocrine system, n=140 , r= 0.037, p=0.66
heart, n=234 , r=−0.021, p=0.75
skin, n=15 , r= 0.061, p=0.83
hair follicle, n=16 , r=−0.048, p=0.86
blood unspecified leukocyte, n=35 , r= 0.031, p=0.86
salivary gland, n=14 , r=−0.037, p=0.9

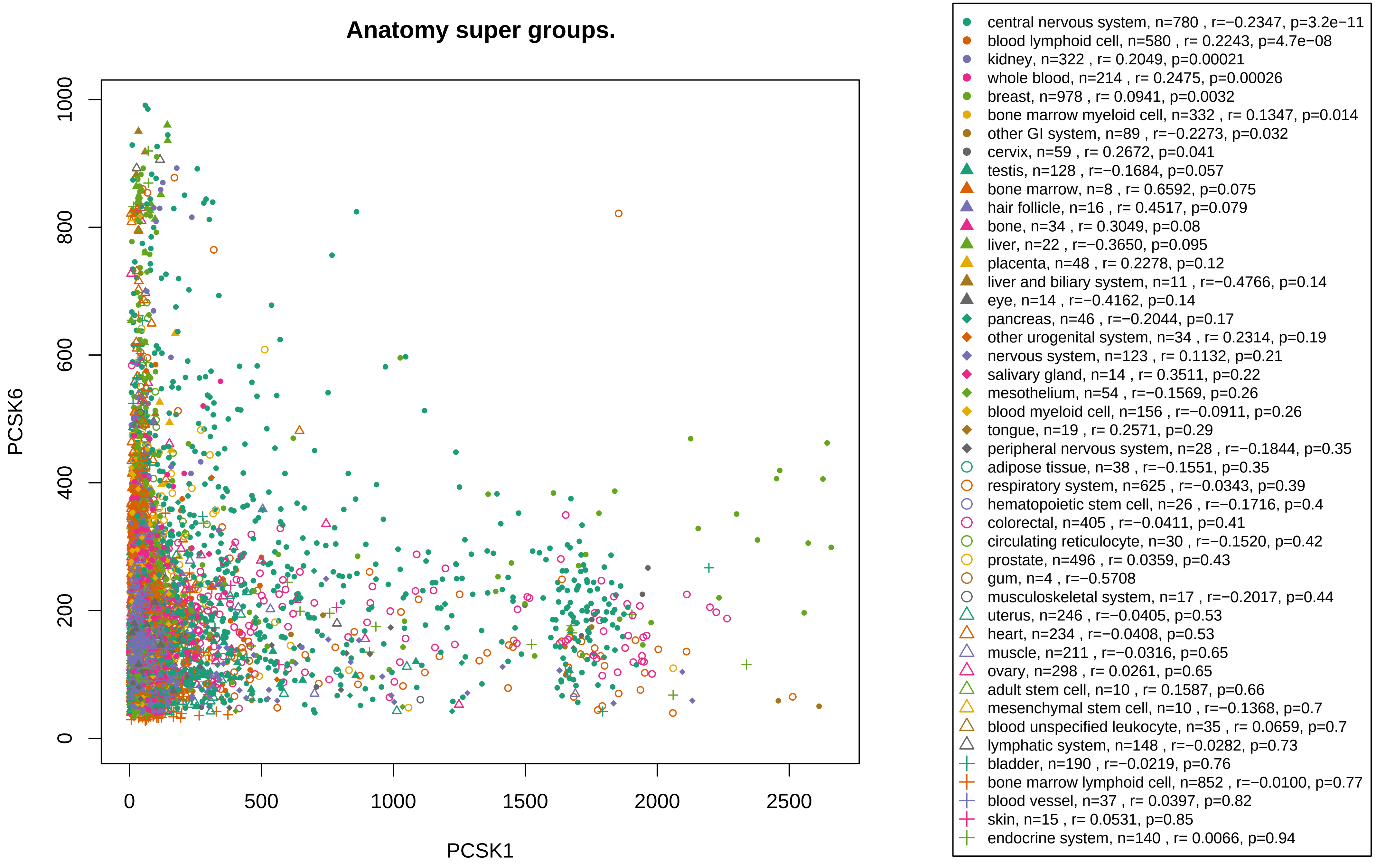

Anatomy super groups.
PCSK6
PCSK1
0
200
400
600
800
1000
0
500
1000
1500
2000
2500
central nervous system, n=780 , r=−0.2347, p=3.2e−11
blood lymphoid cell, n=580 , r= 0.2243, p=4.7e−08
kidney, n=322 , r= 0.2049, p=0.00021
whole blood, n=214 , r= 0.2475, p=0.00026
breast, n=978 , r= 0.0941, p=0.0032
bone marrow myeloid cell, n=332 , r= 0.1347, p=0.014
other GI system, n=89 , r=−0.2273, p=0.032
cervix, n=59 , r= 0.2672, p=0.041
testis, n=128 , r=−0.1684, p=0.057
bone marrow, n=8 , r= 0.6592, p=0.075
hair follicle, n=16 , r= 0.4517, p=0.079
bone, n=34 , r= 0.3049, p=0.08
liver, n=22 , r=−0.3650, p=0.095
placenta, n=48 , r= 0.2278, p=0.12
liver and biliary system, n=11 , r=−0.4766, p=0.14
eye, n=14 , r=−0.4162, p=0.14
pancreas, n=46 , r=−0.2044, p=0.17
other urogenital system, n=34 , r= 0.2314, p=0.19
nervous system, n=123 , r= 0.1132, p=0.21
salivary gland, n=14 , r= 0.3511, p=0.22
mesothelium, n=54 , r=−0.1569, p=0.26
blood myeloid cell, n=156 , r=−0.0911, p=0.26
tongue, n=19 , r= 0.2571, p=0.29
peripheral nervous system, n=28 , r=−0.1844, p=0.35
adipose tissue, n=38 , r=−0.1551, p=0.35
respiratory system, n=625 , r=−0.0343, p=0.39
hematopoietic stem cell, n=26 , r=−0.1716, p=0.4
colorectal, n=405 , r=−0.0411, p=0.41
circulating reticulocyte, n=30 , r=−0.1520, p=0.42
prostate, n=496 , r= 0.0359, p=0.43
gum, n=4 , r=−0.5708
musculoskeletal system, n=17 , r=−0.2017, p=0.44
uterus, n=246 , r=−0.0405, p=0.53
heart, n=234 , r=−0.0408, p=0.53
muscle, n=211 , r=−0.0316, p=0.65
ovary, n=298 , r= 0.0261, p=0.65
adult stem cell, n=10 , r= 0.1587, p=0.66
mesenchymal stem cell, n=10 , r=−0.1368, p=0.7
blood unspecified leukocyte, n=35 , r= 0.0659, p=0.7
lymphatic system, n=148 , r=−0.0282, p=0.73
bladder, n=190 , r=−0.0219, p=0.76
bone marrow lymphoid cell, n=852 , r=−0.0100, p=0.77
blood vessel, n=37 , r= 0.0397, p=0.82
skin, n=15 , r= 0.0531, p=0.85
endocrine system, n=140 , r= 0.0066, p=0.94

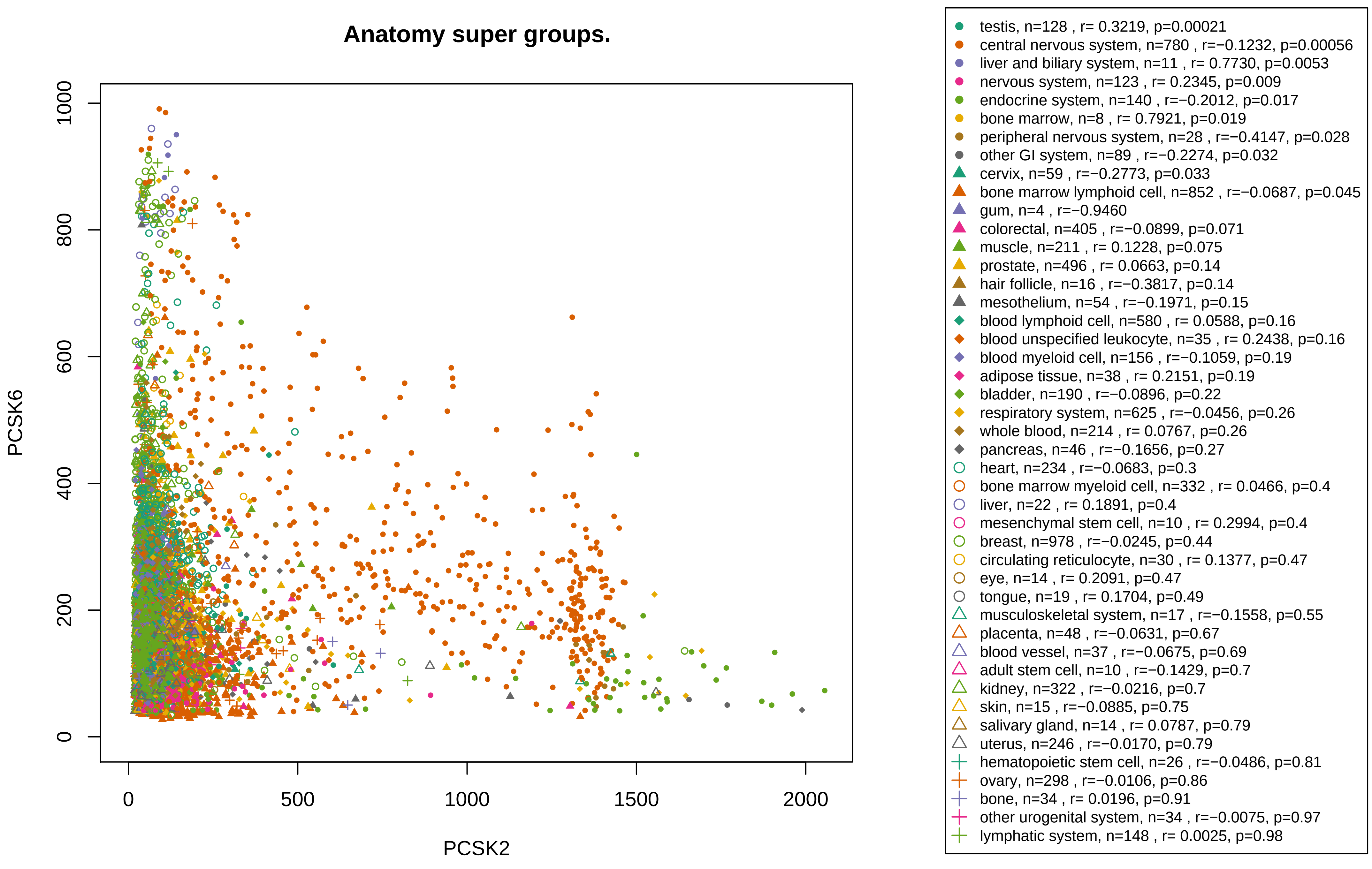
Anatomy super groups.
PCSK6
PCSK2
0
200
400
600
800
1000
0
500
1000
1500
2000
testis, n=128 , r= 0.3219, p=0.00021
central nervous system, n=780 , r=−0.1232, p=0.00056
liver and biliary system, n=11 , r= 0.7730, p=0.0053
nervous system, n=123 , r= 0.2345, p=0.009
endocrine system, n=140 , r=−0.2012, p=0.017
bone marrow, n=8 , r= 0.7921, p=0.019
peripheral nervous system, n=28 , r=−0.4147, p=0.028
other GI system, n=89 , r=−0.2274, p=0.032
cervix, n=59 , r=−0.2773, p=0.033
bone marrow lymphoid cell, n=852 , r=−0.0687, p=0.045
gum, n=4 , r=−0.9460
colorectal, n=405 , r=−0.0899, p=0.071
muscle, n=211 , r= 0.1228, p=0.075
prostate, n=496 , r= 0.0663, p=0.14
hair follicle, n=16 , r=−0.3817, p=0.14
mesothelium, n=54 , r=−0.1971, p=0.15
blood lymphoid cell, n=580 , r= 0.0588, p=0.16
blood unspecified leukocyte, n=35 , r= 0.2438, p=0.16
blood myeloid cell, n=156 , r=−0.1059, p=0.19
adipose tissue, n=38 , r= 0.2151, p=0.19
bladder, n=190 , r=−0.0896, p=0.22
respiratory system, n=625 , r=−0.0456, p=0.26
whole blood, n=214 , r= 0.0767, p=0.26
pancreas, n=46 , r=−0.1656, p=0.27
heart, n=234 , r=−0.0683, p=0.3
bone marrow myeloid cell, n=332 , r= 0.0466, p=0.4
liver, n=22 , r= 0.1891, p=0.4
mesenchymal stem cell, n=10 , r= 0.2994, p=0.4
breast, n=978 , r=−0.0245, p=0.44
circulating reticulocyte, n=30 , r= 0.1377, p=0.47
eye, n=14 , r= 0.2091, p=0.47
tongue, n=19 , r= 0.1704, p=0.49
musculoskeletal system, n=17 , r=−0.1558, p=0.55
placenta, n=48 , r=−0.0631, p=0.67
blood vessel, n=37 , r=−0.0675, p=0.69
adult stem cell, n=10 , r=−0.1429, p=0.7
kidney, n=322 , r=−0.0216, p=0.7
skin, n=15 , r=−0.0885, p=0.75
salivary gland, n=14 , r= 0.0787, p=0.79
uterus, n=246 , r=−0.0170, p=0.79
hematopoietic stem cell, n=26 , r=−0.0486, p=0.81
ovary, n=298 , r=−0.0106, p=0.86
bone, n=34 , r= 0.0196, p=0.91
other urogenital system, n=34 , r=−0.0075, p=0.97
lymphatic system, n=148 , r= 0.0025, p=0.98

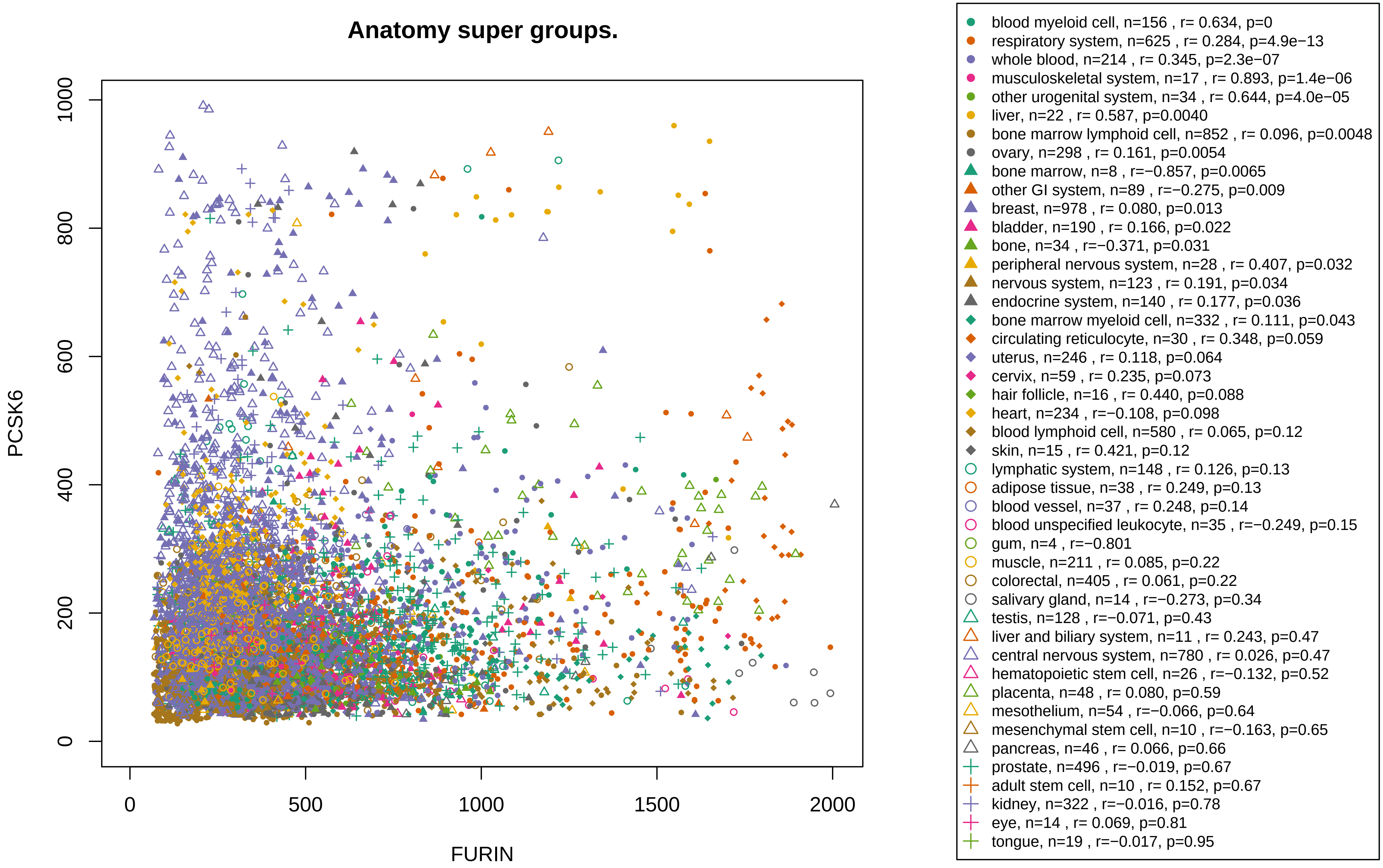

Anatomy super groups.
PCSK6
FURIN
0
200
400
600
800
1000
0
500
1000
1500
2000
blood myeloid cell, n=156 , r= 0.634, p=0
respiratory system, n=625 , r= 0.284, p=4.9e−13
whole blood, n=214 , r= 0.345, p=2.3e−07
musculoskeletal system, n=17 , r= 0.893, p=1.4e−06
other urogenital system, n=34 , r= 0.644, p=4.0e−05
liver, n=22 , r= 0.587, p=0.0040
bone marrow lymphoid cell, n=852 , r= 0.096, p=0.0048
ovary, n=298 , r= 0.161, p=0.0054
bone marrow, n=8 , r=−0.857, p=0.0065
other GI system, n=89 , r=−0.275, p=0.009
breast, n=978 , r= 0.080, p=0.013
bladder, n=190 , r= 0.166, p=0.022
bone, n=34 , r=−0.371, p=0.031
peripheral nervous system, n=28 , r= 0.407, p=0.032
nervous system, n=123 , r= 0.191, p=0.034
endocrine system, n=140 , r= 0.177, p=0.036
bone marrow myeloid cell, n=332 , r= 0.111, p=0.043
circulating reticulocyte, n=30 , r= 0.348, p=0.059
uterus, n=246 , r= 0.118, p=0.064
cervix, n=59 , r= 0.235, p=0.073
hair follicle, n=16 , r= 0.440, p=0.088
heart, n=234 , r=−0.108, p=0.098
blood lymphoid cell, n=580 , r= 0.065, p=0.12
skin, n=15 , r= 0.421, p=0.12
lymphatic system, n=148 , r= 0.126, p=0.13
adipose tissue, n=38 , r= 0.249, p=0.13
blood vessel, n=37 , r= 0.248, p=0.14
blood unspecified leukocyte, n=35 , r=−0.249, p=0.15
gum, n=4 , r=−0.801
muscle, n=211 , r= 0.085, p=0.22
colorectal, n=405 , r= 0.061, p=0.22
salivary gland, n=14 , r=−0.273, p=0.34
testis, n=128 , r=−0.071, p=0.43
liver and biliary system, n=11 , r= 0.243, p=0.47
central nervous system, n=780 , r= 0.026, p=0.47
hematopoietic stem cell, n=26 , r=−0.132, p=0.52
placenta, n=48 , r= 0.080, p=0.59
mesothelium, n=54 , r=−0.066, p=0.64
mesenchymal stem cell, n=10 , r=−0.163, p=0.65
pancreas, n=46 , r= 0.066, p=0.66
prostate, n=496 , r=−0.019, p=0.67
adult stem cell, n=10 , r= 0.152, p=0.67
kidney, n=322 , r=−0.016, p=0.78
eye, n=14 , r= 0.069, p=0.81
tongue, n=19 , r=−0.017, p=0.95

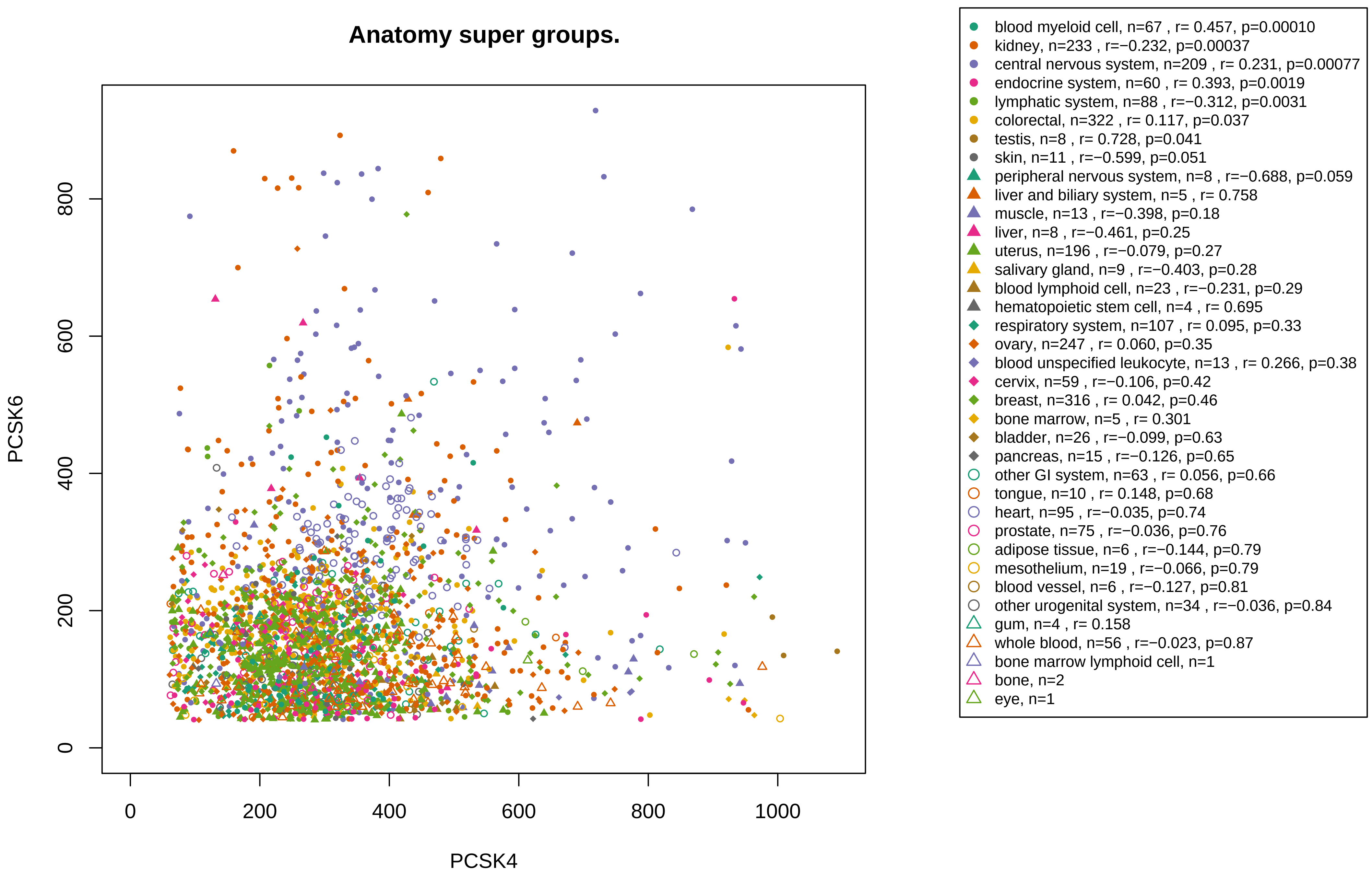

Anatomy super groups.
PCSK6
PCSK4
0
200
400
600
800
1000
blood myeloid cell, n=67 , r= 0.457, p=0.00010
kidney, n=233 , r=−0.232, p=0.00037
central nervous system, n=209 , r= 0.231, p=0.00077
endocrine system, n=60 , r= 0.393, p=0.0019
lymphatic system, n=88 , r=−0.312, p=0.0031
colorectal, n=322 , r= 0.117, p=0.037
testis, n=8 , r= 0.728, p=0.041
skin, n=11 , r=−0.599, p=0.051
peripheral nervous system, n=8 , r=−0.688, p=0.059
liver and biliary system, n=5 , r= 0.758
muscle, n=13 , r=−0.398, p=0.18
liver, n=8 , r=−0.461, p=0.25
uterus, n=196 , r=−0.079, p=0.27
salivary gland, n=9 , r=−0.403, p=0.28
blood lymphoid cell, n=23 , r=−0.231, p=0.29
hematopoietic stem cell, n=4 , r= 0.695
respiratory system, n=107 , r= 0.095, p=0.33
ovary, n=247 , r= 0.060, p=0.35
blood unspecified leukocyte, n=13 , r= 0.266, p=0.38
cervix, n=59 , r=−0.106, p=0.42
breast, n=316 , r= 0.042, p=0.46
bone marrow, n=5 , r= 0.301
bladder, n=26 , r=−0.099, p=0.63
pancreas, n=15 , r=−0.126, p=0.65
other GI system, n=63 , r= 0.056, p=0.66
tongue, n=10 , r= 0.148, p=0.68
heart, n=95 , r=−0.035, p=0.74
prostate, n=75 , r=−0.036, p=0.76
adipose tissue, n=6 , r=−0.144, p=0.79
mesothelium, n=19 , r=−0.066, p=0.79
blood vessel, n=6 , r=−0.127, p=0.81
other urogenital system, n=34 , r=−0.036, p=0.84
gum, n=4 , r= 0.158
whole blood, n=56 , r=−0.023, p=0.87
bone marrow lymphoid cell, n=1
bone, n=2
eye, n=1

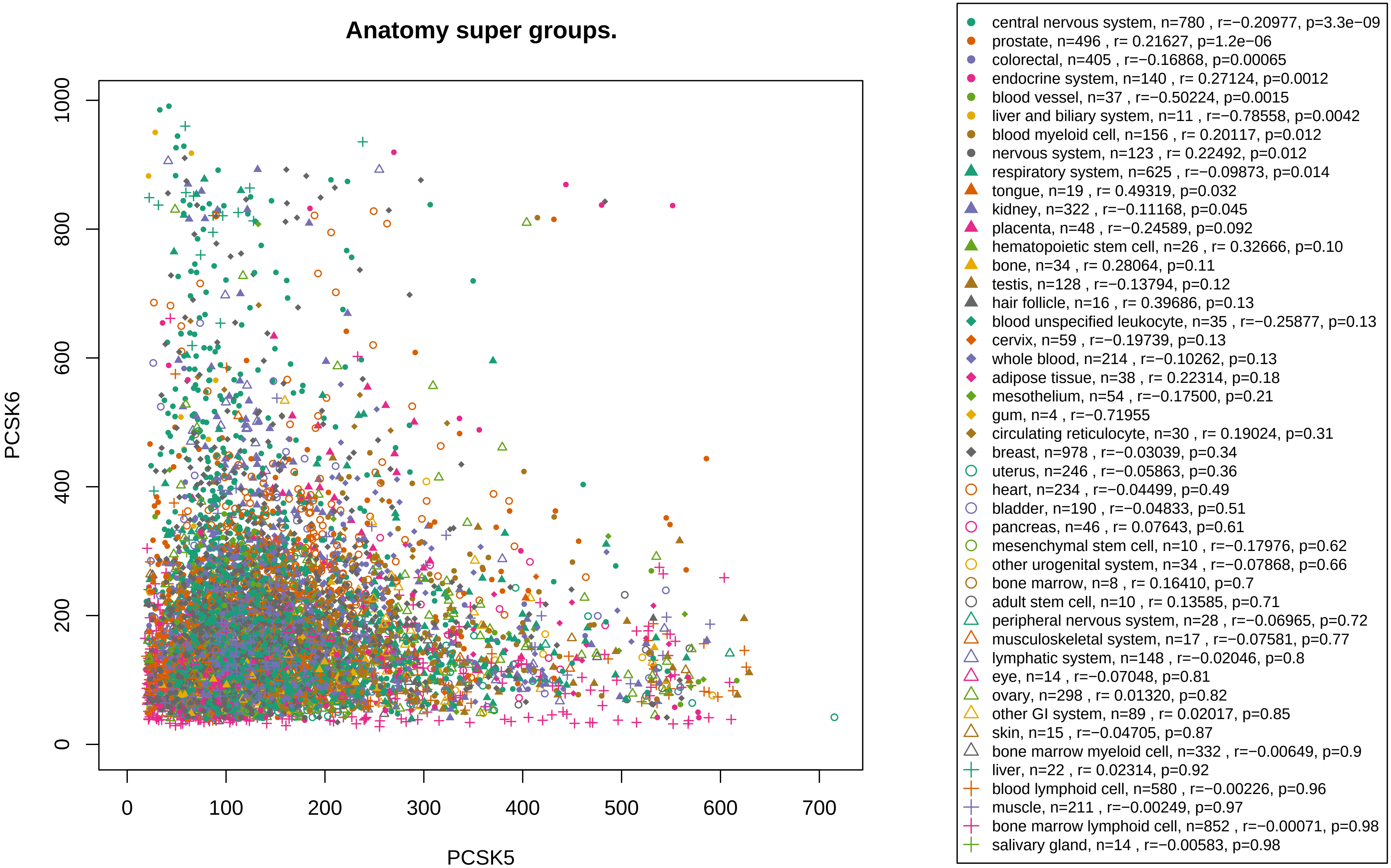
Anatomy super groups.
PCSK6
PCSK5
0
200
400
600
800
1000
0
100
200
300
400
500
600
700
central nervous system, n=780 , r=−0.20977, p=3.3e−09
prostate, n=496 , r= 0.21627, p=1.2e−06
colorectal, n=405 , r=−0.16868, p=0.00065
endocrine system, n=140 , r= 0.27124, p=0.0012
blood vessel, n=37 , r=−0.50224, p=0.0015
liver and biliary system, n=11 , r=−0.78558, p=0.0042
blood myeloid cell, n=156 , r= 0.20117, p=0.012
nervous system, n=123 , r= 0.22492, p=0.012
respiratory system, n=625 , r=−0.09873, p=0.014
tongue, n=19 , r= 0.49319, p=0.032
kidney, n=322 , r=−0.11168, p=0.045
placenta, n=48 , r=−0.24589, p=0.092
hematopoietic stem cell, n=26 , r= 0.32666, p=0.10
bone, n=34 , r= 0.28064, p=0.11
testis, n=128 , r=−0.13794, p=0.12
hair follicle, n=16 , r= 0.39686, p=0.13
blood unspecified leukocyte, n=35 , r=−0.25877, p=0.13
cervix, n=59 , r=−0.19739, p=0.13
whole blood, n=214 , r=−0.10262, p=0.13
adipose tissue, n=38 , r= 0.22314, p=0.18
mesothelium, n=54 , r=−0.17500, p=0.21
gum, n=4 , r=−0.71955
circulating reticulocyte, n=30 , r= 0.19024, p=0.31
breast, n=978 , r=−0.03039, p=0.34
uterus, n=246 , r=−0.05863, p=0.36
heart, n=234 , r=−0.04499, p=0.49
bladder, n=190 , r=−0.04833, p=0.51
pancreas, n=46 , r= 0.07643, p=0.61
mesenchymal stem cell, n=10 , r=−0.17976, p=0.62
other urogenital system, n=34 , r=−0.07868, p=0.66
bone marrow, n=8 , r= 0.16410, p=0.7
adult stem cell, n=10 , r= 0.13585, p=0.71
peripheral nervous system, n=28 , r=−0.06965, p=0.72
musculoskeletal system, n=17 , r=−0.07581, p=0.77
lymphatic system, n=148 , r=−0.02046, p=0.8
eye, n=14 , r=−0.07048, p=0.81
ovary, n=298 , r= 0.01320, p=0.82
other GI system, n=89 , r= 0.02017, p=0.85
skin, n=15 , r=−0.04705, p=0.87
bone marrow myeloid cell, n=332 , r=−0.00649, p=0.9
liver, n=22 , r= 0.02314, p=0.92
blood lymphoid cell, n=580 , r=−0.00226, p=0.96
muscle, n=211 , r=−0.00249, p=0.97
bone marrow lymphoid cell, n=852 , r=−0.00071, p=0.98
salivary gland, n=14 , r=−0.00583, p=0.98

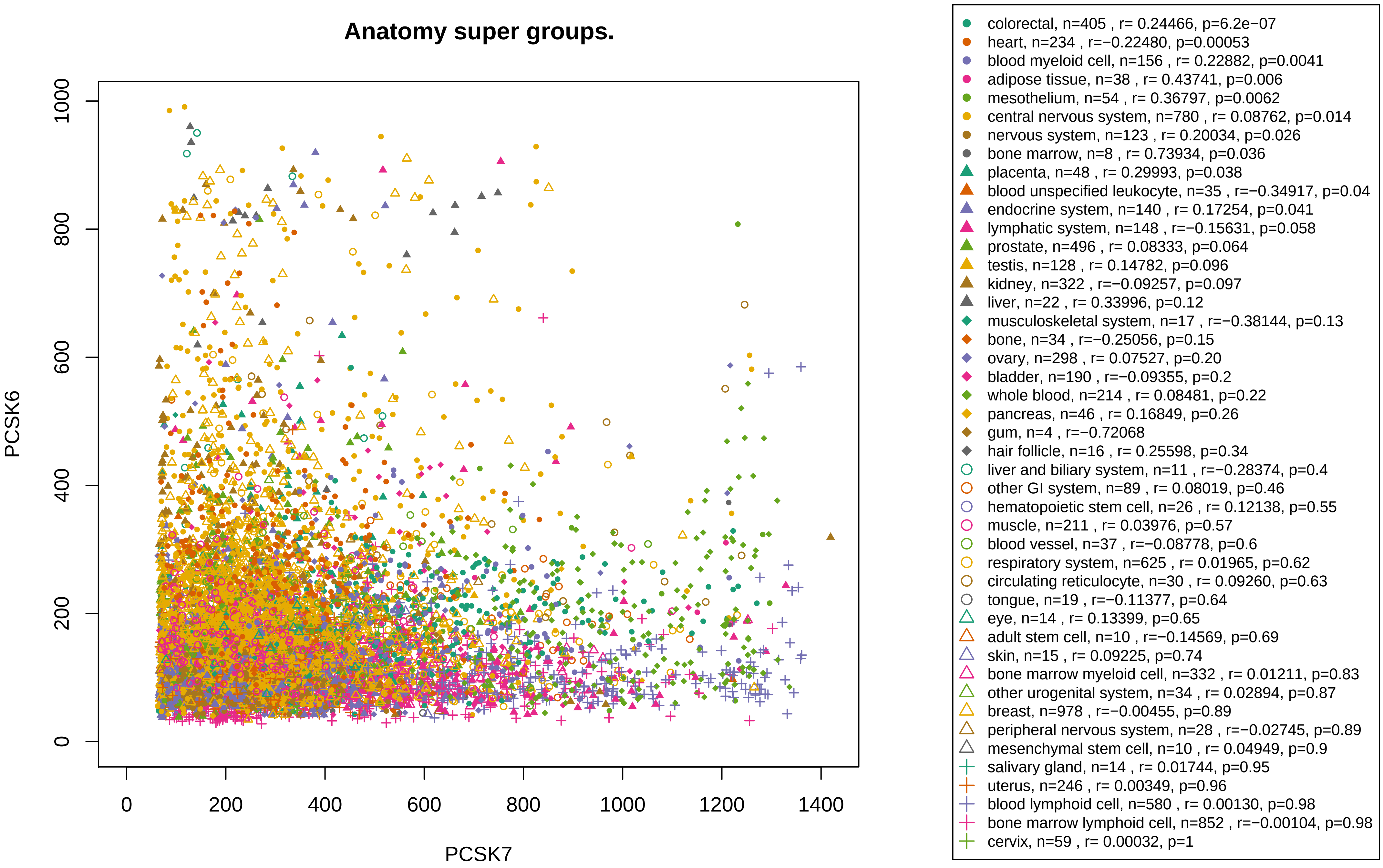
Anatomy super groups.
PCSK6
PCSK7
0
200
400
600
800
1000
1200
1400
colorectal, n=405 , r= 0.24466, p=6.2e−07
heart, n=234 , r=−0.22480, p=0.00053
blood myeloid cell, n=156 , r= 0.22882, p=0.0041
adipose tissue, n=38 , r= 0.43741, p=0.006
mesothelium, n=54 , r= 0.36797, p=0.0062
central nervous system, n=780 , r= 0.08762, p=0.014
nervous system, n=123 , r= 0.20034, p=0.026
bone marrow, n=8 , r= 0.73934, p=0.036
placenta, n=48 , r= 0.29993, p=0.038
blood unspecified leukocyte, n=35 , r=−0.34917, p=0.04
endocrine system, n=140 , r= 0.17254, p=0.041
lymphatic system, n=148 , r=−0.15631, p=0.058
prostate, n=496 , r= 0.08333, p=0.064
testis, n=128 , r= 0.14782, p=0.096
kidney, n=322 , r=−0.09257, p=0.097
liver, n=22 , r= 0.33996, p=0.12
musculoskeletal system, n=17 , r=−0.38144, p=0.13
bone, n=34 , r=−0.25056, p=0.15
ovary, n=298 , r= 0.07527, p=0.20
bladder, n=190 , r=−0.09355, p=0.2
whole blood, n=214 , r= 0.08481, p=0.22
pancreas, n=46 , r= 0.16849, p=0.26
gum, n=4 , r=−0.72068
hair follicle, n=16 , r= 0.25598, p=0.34
liver and biliary system, n=11 , r=−0.28374, p=0.4
other GI system, n=89 , r= 0.08019, p=0.46
hematopoietic stem cell, n=26 , r= 0.12138, p=0.55
muscle, n=211 , r= 0.03976, p=0.57
blood vessel, n=37 , r=−0.08778, p=0.6
respiratory system, n=625 , r= 0.01965, p=0.62
circulating reticulocyte, n=30 , r= 0.09260, p=0.63
tongue, n=19 , r=−0.11377, p=0.64
eye, n=14 , r= 0.13399, p=0.65
adult stem cell, n=10 , r=−0.14569, p=0.69
skin, n=15 , r= 0.09225, p=0.74
bone marrow myeloid cell, n=332 , r= 0.01211, p=0.83
other urogenital system, n=34 , r= 0.02894, p=0.87
breast, n=978 , r=−0.00455, p=0.89
peripheral nervous system, n=28 , r=−0.02745, p=0.89
mesenchymal stem cell, n=10 , r= 0.04949, p=0.9
salivary gland, n=14 , r= 0.01744, p=0.95
uterus, n=246 , r= 0.00349, p=0.96
blood lymphoid cell, n=580 , r= 0.00130, p=0.98
bone marrow lymphoid cell, n=852 , r=−0.00104, p=0.98
cervix, n=59 , r= 0.00032, p=1

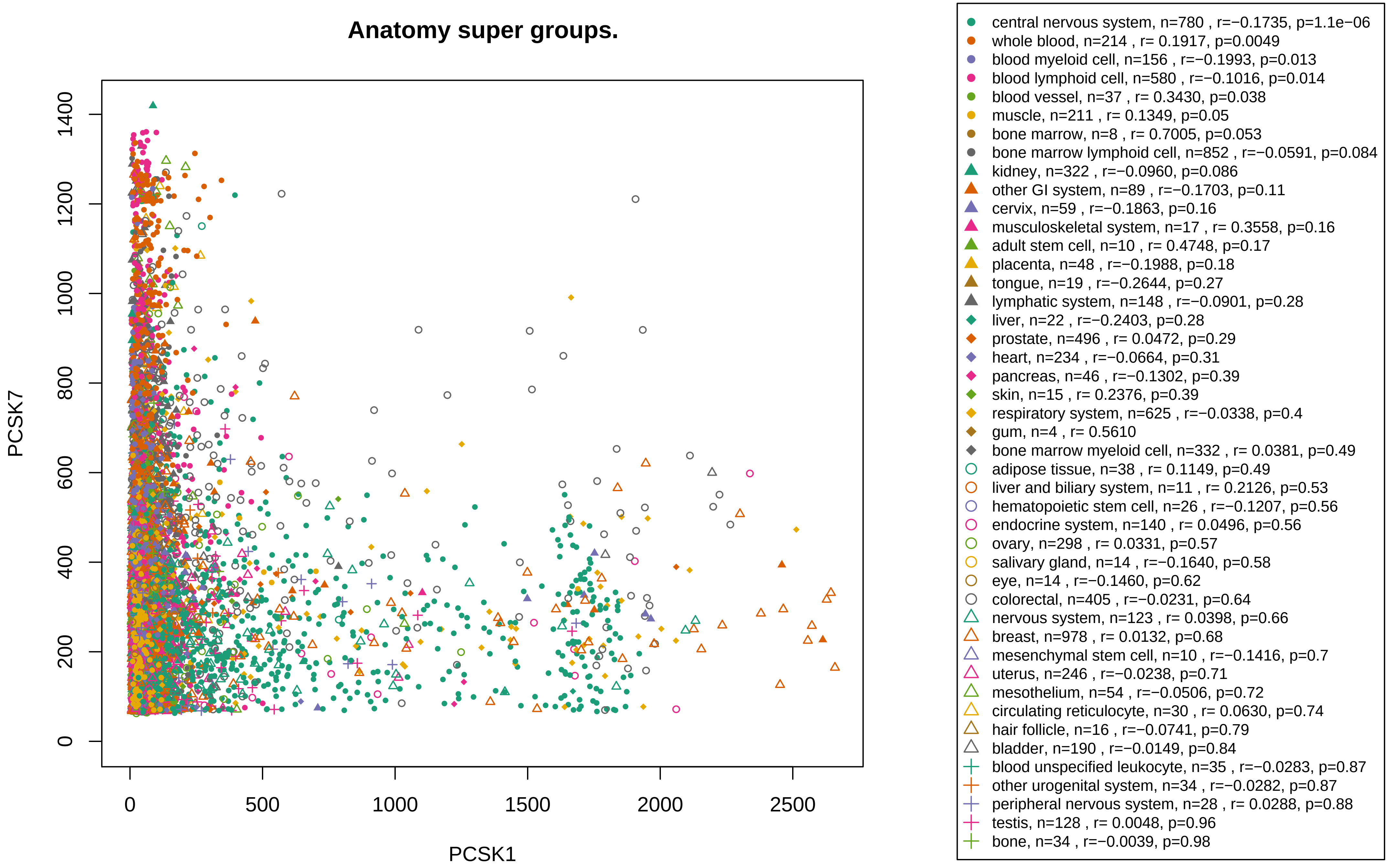

Anatomy super groups.
PCSK7
PCSK1
0
200
400
600
800
1000
1200
1400
0
500
1000
1500
2000
2500
central nervous system, n=780 , r=−0.1735, p=1.1e−06
whole blood, n=214 , r= 0.1917, p=0.0049
blood myeloid cell, n=156 , r=−0.1993, p=0.013
blood lymphoid cell, n=580 , r=−0.1016, p=0.014
blood vessel, n=37 , r= 0.3430, p=0.038
muscle, n=211 , r= 0.1349, p=0.05
bone marrow, n=8 , r= 0.7005, p=0.053
bone marrow lymphoid cell, n=852 , r=−0.0591, p=0.084
kidney, n=322 , r=−0.0960, p=0.086
other GI system, n=89 , r=−0.1703, p=0.11
cervix, n=59 , r=−0.1863, p=0.16
musculoskeletal system, n=17 , r= 0.3558, p=0.16
adult stem cell, n=10 , r= 0.4748, p=0.17
placenta, n=48 , r=−0.1988, p=0.18
tongue, n=19 , r=−0.2644, p=0.27
lymphatic system, n=148 , r=−0.0901, p=0.28
liver, n=22 , r=−0.2403, p=0.28
prostate, n=496 , r= 0.0472, p=0.29
heart, n=234 , r=−0.0664, p=0.31
pancreas, n=46 , r=−0.1302, p=0.39
skin, n=15 , r= 0.2376, p=0.39
respiratory system, n=625 , r=−0.0338, p=0.4
gum, n=4 , r= 0.5610
bone marrow myeloid cell, n=332 , r= 0.0381, p=0.49
adipose tissue, n=38 , r= 0.1149, p=0.49
liver and biliary system, n=11 , r= 0.2126, p=0.53
hematopoietic stem cell, n=26 , r=−0.1207, p=0.56
endocrine system, n=140 , r= 0.0496, p=0.56
ovary, n=298 , r= 0.0331, p=0.57
salivary gland, n=14 , r=−0.1640, p=0.58
eye, n=14 , r=−0.1460, p=0.62
colorectal, n=405 , r=−0.0231, p=0.64
nervous system, n=123 , r= 0.0398, p=0.66
breast, n=978 , r= 0.0132, p=0.68
mesenchymal stem cell, n=10 , r=−0.1416, p=0.7
uterus, n=246 , r=−0.0238, p=0.71
mesothelium, n=54 , r=−0.0506, p=0.72
circulating reticulocyte, n=30 , r= 0.0630, p=0.74
hair follicle, n=16 , r=−0.0741, p=0.79
bladder, n=190 , r=−0.0149, p=0.84
blood unspecified leukocyte, n=35 , r=−0.0283, p=0.87
other urogenital system, n=34 , r=−0.0282, p=0.87
peripheral nervous system, n=28 , r= 0.0288, p=0.88
testis, n=128 , r= 0.0048, p=0.96
bone, n=34 , r=−0.0039, p=0.98

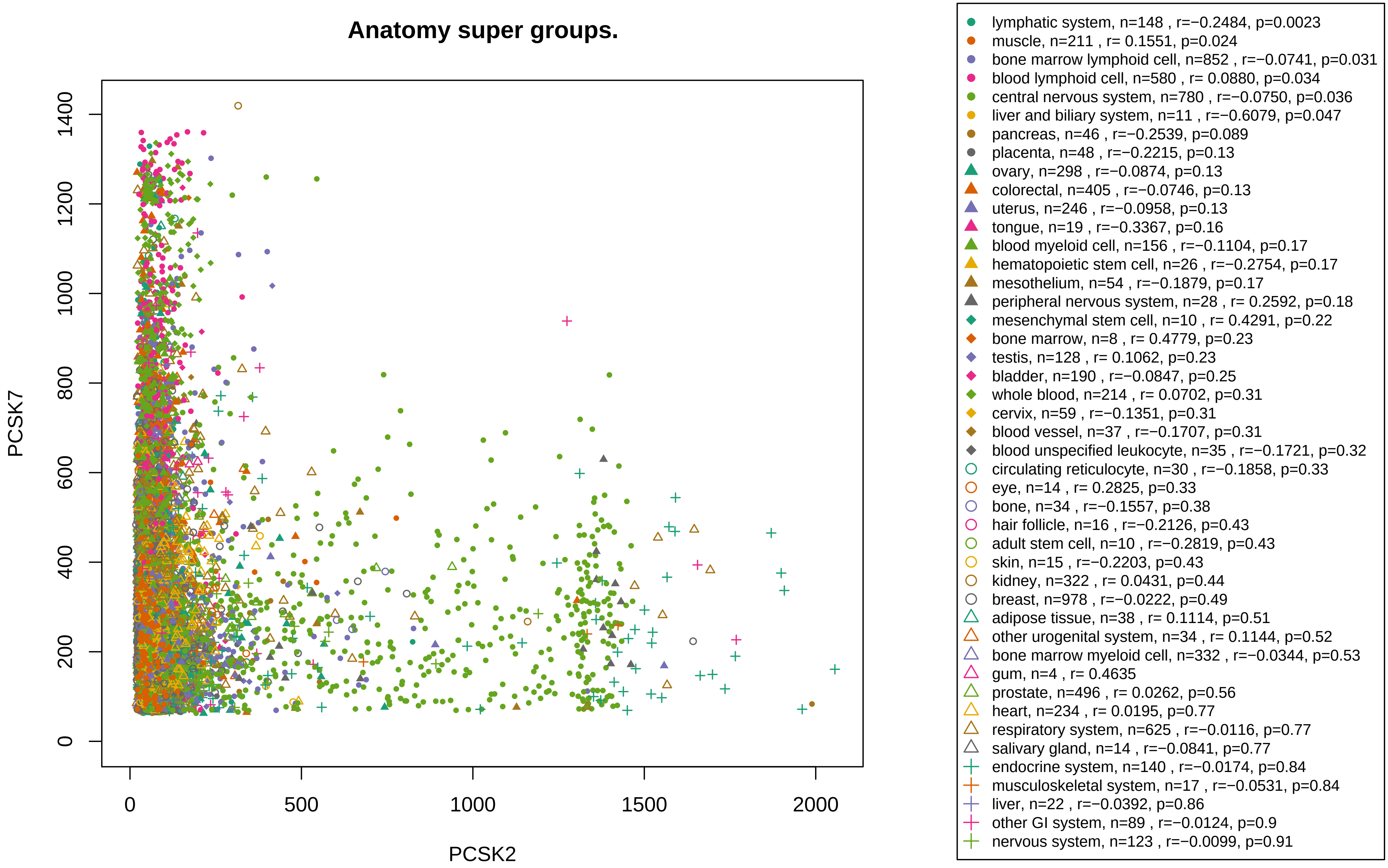
Anatomy super groups.
PCSK7
PCSK2
0
200
400
600
800
1000
1200
1400
0
500
1000
1500
2000
lymphatic system, n=148 , r=−0.2484, p=0.0023
muscle, n=211 , r= 0.1551, p=0.024
bone marrow lymphoid cell, n=852 , r=−0.0741, p=0.031
blood lymphoid cell, n=580 , r= 0.0880, p=0.034
central nervous system, n=780 , r=−0.0750, p=0.036
liver and biliary system, n=11 , r=−0.6079, p=0.047
pancreas, n=46 , r=−0.2539, p=0.089
placenta, n=48 , r=−0.2215, p=0.13
ovary, n=298 , r=−0.0874, p=0.13
colorectal, n=405 , r=−0.0746, p=0.13
uterus, n=246 , r=−0.0958, p=0.13
tongue, n=19 , r=−0.3367, p=0.16
blood myeloid cell, n=156 , r=−0.1104, p=0.17
hematopoietic stem cell, n=26 , r=−0.2754, p=0.17
mesothelium, n=54 , r=−0.1879, p=0.17
peripheral nervous system, n=28 , r= 0.2592, p=0.18
mesenchymal stem cell, n=10 , r= 0.4291, p=0.22
bone marrow, n=8 , r= 0.4779, p=0.23
testis, n=128 , r= 0.1062, p=0.23
bladder, n=190 , r=−0.0847, p=0.25
whole blood, n=214 , r= 0.0702, p=0.31
cervix, n=59 , r=−0.1351, p=0.31
blood vessel, n=37 , r=−0.1707, p=0.31
blood unspecified leukocyte, n=35 , r=−0.1721, p=0.32
circulating reticulocyte, n=30 , r=−0.1858, p=0.33
eye, n=14 , r= 0.2825, p=0.33
bone, n=34 , r=−0.1557, p=0.38
hair follicle, n=16 , r=−0.2126, p=0.43
adult stem cell, n=10 , r=−0.2819, p=0.43
skin, n=15 , r=−0.2203, p=0.43
kidney, n=322 , r= 0.0431, p=0.44
breast, n=978 , r=−0.0222, p=0.49
adipose tissue, n=38 , r= 0.1114, p=0.51
other urogenital system, n=34 , r= 0.1144, p=0.52
bone marrow myeloid cell, n=332 , r=−0.0344, p=0.53
gum, n=4 , r= 0.4635
prostate, n=496 , r= 0.0262, p=0.56
heart, n=234 , r= 0.0195, p=0.77
respiratory system, n=625 , r=−0.0116, p=0.77
salivary gland, n=14 , r=−0.0841, p=0.77
endocrine system, n=140 , r=−0.0174, p=0.84
musculoskeletal system, n=17 , r=−0.0531, p=0.84
liver, n=22 , r=−0.0392, p=0.86
other GI system, n=89 , r=−0.0124, p=0.9
nervous system, n=123 , r=−0.0099, p=0.91

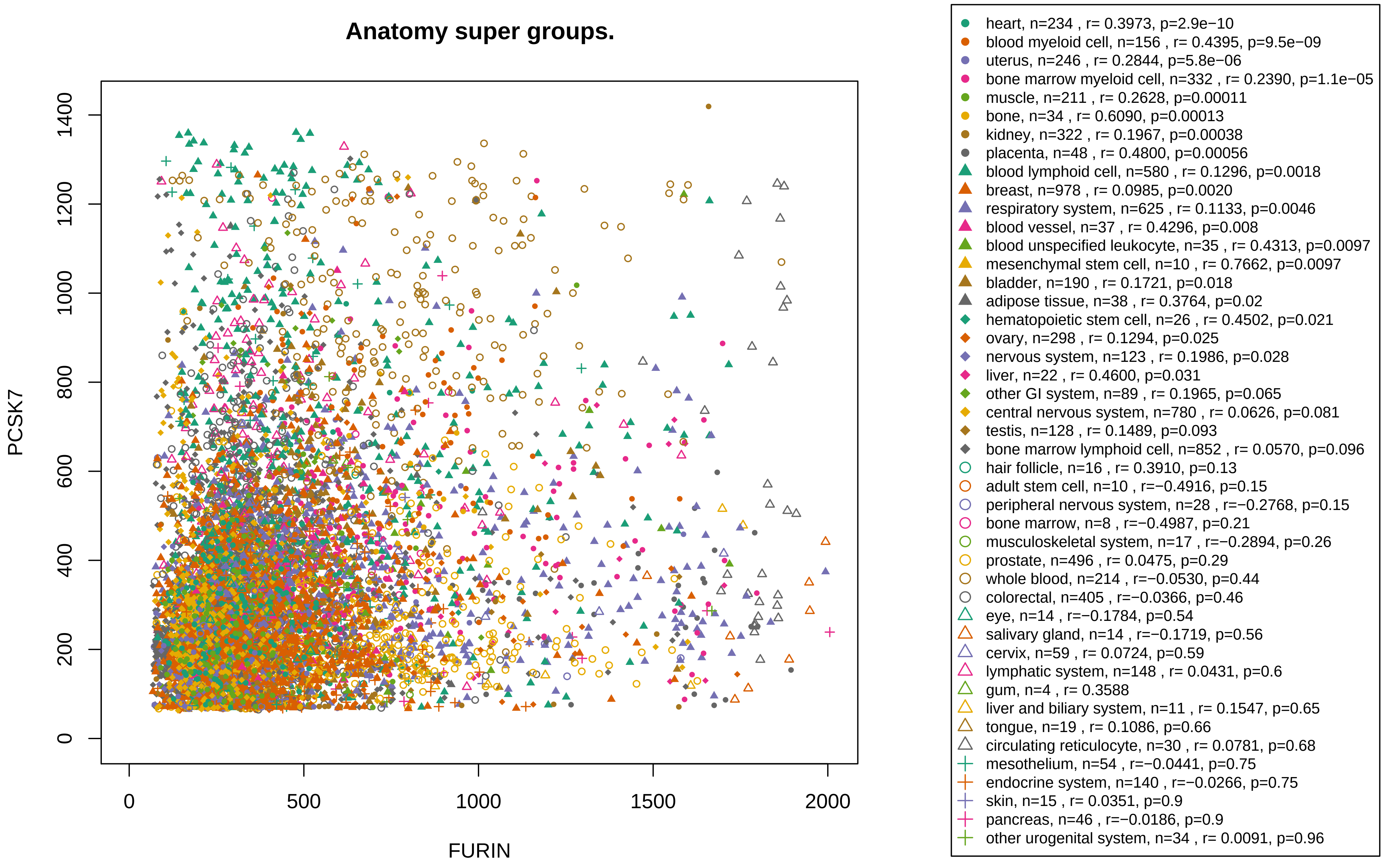
Anatomy super groups.
PCSK7
FURIN
0
200
400
600
800
1000
1200
1400
0
500
1000
1500
2000
heart, n=234 , r= 0.3973, p=2.9e−10
blood myeloid cell, n=156 , r= 0.4395, p=9.5e−09
uterus, n=246 , r= 0.2844, p=5.8e−06
bone marrow myeloid cell, n=332 , r= 0.2390, p=1.1e−05
muscle, n=211 , r= 0.2628, p=0.00011
bone, n=34 , r= 0.6090, p=0.00013
kidney, n=322 , r= 0.1967, p=0.00038
placenta, n=48 , r= 0.4800, p=0.00056
blood lymphoid cell, n=580 , r= 0.1296, p=0.0018
breast, n=978 , r= 0.0985, p=0.0020
respiratory system, n=625 , r= 0.1133, p=0.0046
blood vessel, n=37 , r= 0.4296, p=0.008
blood unspecified leukocyte, n=35 , r= 0.4313, p=0.0097
mesenchymal stem cell, n=10 , r= 0.7662, p=0.0097
bladder, n=190 , r= 0.1721, p=0.018
adipose tissue, n=38 , r= 0.3764, p=0.02
hematopoietic stem cell, n=26 , r= 0.4502, p=0.021
ovary, n=298 , r= 0.1294, p=0.025
nervous system, n=123 , r= 0.1986, p=0.028
liver, n=22 , r= 0.4600, p=0.031
other GI system, n=89 , r= 0.1965, p=0.065
central nervous system, n=780 , r= 0.0626, p=0.081
testis, n=128 , r= 0.1489, p=0.093
bone marrow lymphoid cell, n=852 , r= 0.0570, p=0.096
hair follicle, n=16 , r= 0.3910, p=0.13
adult stem cell, n=10 , r=−0.4916, p=0.15
peripheral nervous system, n=28 , r=−0.2768, p=0.15
bone marrow, n=8 , r=−0.4987, p=0.21
musculoskeletal system, n=17 , r=−0.2894, p=0.26
prostate, n=496 , r= 0.0475, p=0.29
whole blood, n=214 , r=−0.0530, p=0.44
colorectal, n=405 , r=−0.0366, p=0.46
eye, n=14 , r=−0.1784, p=0.54
salivary gland, n=14 , r=−0.1719, p=0.56
cervix, n=59 , r= 0.0724, p=0.59
lymphatic system, n=148 , r= 0.0431, p=0.6
gum, n=4 , r= 0.3588
liver and biliary system, n=11 , r= 0.1547, p=0.65
tongue, n=19 , r= 0.1086, p=0.66
circulating reticulocyte, n=30 , r= 0.0781, p=0.68
mesothelium, n=54 , r=−0.0441, p=0.75
endocrine system, n=140 , r=−0.0266, p=0.75
skin, n=15 , r= 0.0351, p=0.9
pancreas, n=46 , r=−0.0186, p=0.9
other urogenital system, n=34 , r= 0.0091, p=0.96

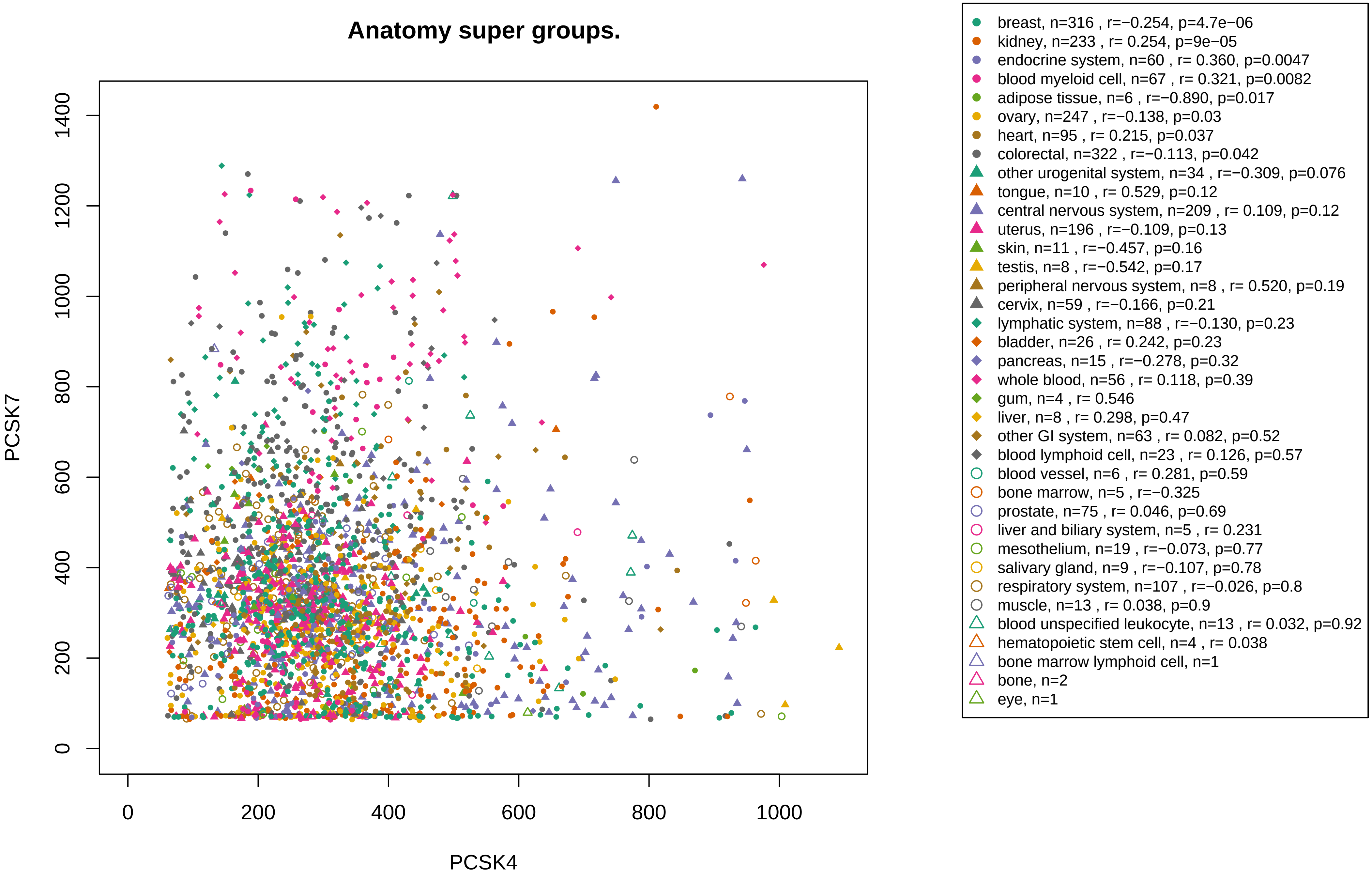
Anatomy super groups.
PCSK7
PCSK4
0
200
400
600
800
1000
1200
1400
0
200
400
600
800
1000
breast, n=316 , r=−0.254, p=4.7e−06
kidney, n=233 , r= 0.254, p=9e−05
endocrine system, n=60 , r= 0.360, p=0.0047
blood myeloid cell, n=67 , r= 0.321, p=0.0082
adipose tissue, n=6 , r=−0.890, p=0.017
ovary, n=247 , r=−0.138, p=0.03
heart, n=95 , r= 0.215, p=0.037
colorectal, n=322 , r=−0.113, p=0.042
other urogenital system, n=34 , r=−0.309, p=0.076
tongue, n=10 , r= 0.529, p=0.12
central nervous system, n=209 , r= 0.109, p=0.12
uterus, n=196 , r=−0.109, p=0.13
skin, n=11 , r=−0.457, p=0.16
testis, n=8 , r=−0.542, p=0.17
peripheral nervous system, n=8 , r= 0.520, p=0.19
cervix, n=59 , r=−0.166, p=0.21
lymphatic system, n=88 , r=−0.130, p=0.23
bladder, n=26 , r= 0.242, p=0.23
pancreas, n=15 , r=−0.278, p=0.32
whole blood, n=56 , r= 0.118, p=0.39
gum, n=4 , r= 0.546
liver, n=8 , r= 0.298, p=0.47
other GI system, n=63 , r= 0.082, p=0.52
blood lymphoid cell, n=23 , r= 0.126, p=0.57
blood vessel, n=6 , r= 0.281, p=0.59
bone marrow, n=5 , r=−0.325
prostate, n=75 , r= 0.046, p=0.69
liver and biliary system, n=5 , r= 0.231
mesothelium, n=19 , r=−0.073, p=0.77
salivary gland, n=9 , r=−0.107, p=0.78
respiratory system, n=107 , r=−0.026, p=0.8
muscle, n=13 , r= 0.038, p=0.9
blood unspecified leukocyte, n=13 , r= 0.032, p=0.92
hematopoietic stem cell, n=4 , r= 0.038
bone marrow lymphoid cell, n=1
bone, n=2
eye, n=1

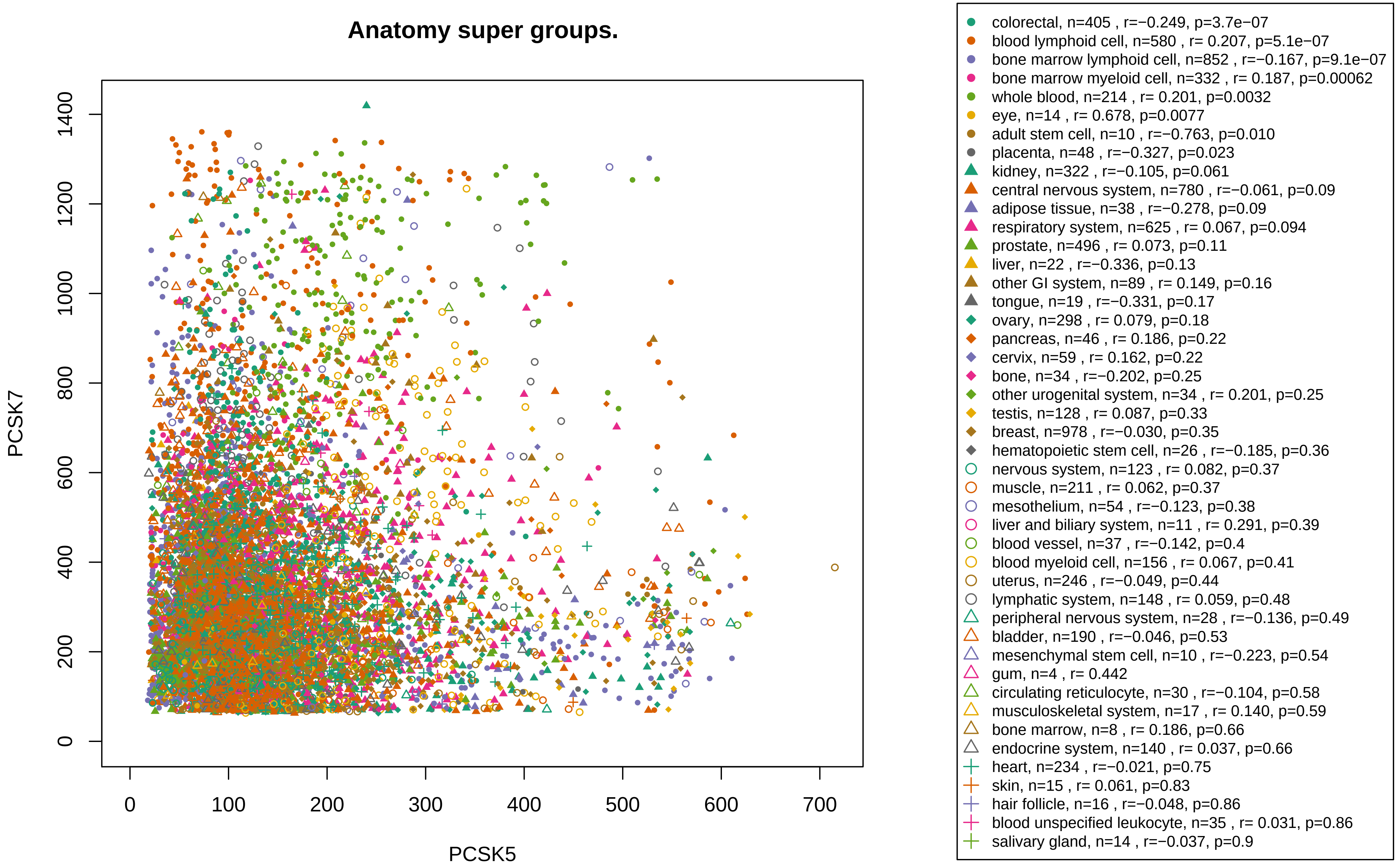
Anatomy super groups.
PCSK7
PCSK5
0
200
400
600
800
1000
1200
1400
0
100
200
300
400
500
600
700
colorectal, n=405 , r=−0.249, p=3.7e−07
blood lymphoid cell, n=580 , r= 0.207, p=5.1e−07
bone marrow lymphoid cell, n=852 , r=−0.167, p=9.1e−07
bone marrow myeloid cell, n=332 , r= 0.187, p=0.00062
whole blood, n=214 , r= 0.201, p=0.0032
eye, n=14 , r= 0.678, p=0.0077
adult stem cell, n=10 , r=−0.763, p=0.010
placenta, n=48 , r=−0.327, p=0.023
kidney, n=322 , r=−0.105, p=0.061
central nervous system, n=780 , r=−0.061, p=0.09
adipose tissue, n=38 , r=−0.278, p=0.09
respiratory system, n=625 , r= 0.067, p=0.094
prostate, n=496 , r= 0.073, p=0.11
liver, n=22 , r=−0.336, p=0.13
other GI system, n=89 , r= 0.149, p=0.16
tongue, n=19 , r=−0.331, p=0.17
ovary, n=298 , r= 0.079, p=0.18
pancreas, n=46 , r= 0.186, p=0.22
cervix, n=59 , r= 0.162, p=0.22
bone, n=34 , r=−0.202, p=0.25
other urogenital system, n=34 , r= 0.201, p=0.25
testis, n=128 , r= 0.087, p=0.33
breast, n=978 , r=−0.030, p=0.35
hematopoietic stem cell, n=26 , r=−0.185, p=0.36
nervous system, n=123 , r= 0.082, p=0.37
muscle, n=211 , r= 0.062, p=0.37
mesothelium, n=54 , r=−0.123, p=0.38
liver and biliary system, n=11 , r= 0.291, p=0.39
blood vessel, n=37 , r=−0.142, p=0.4
blood myeloid cell, n=156 , r= 0.067, p=0.41
uterus, n=246 , r=−0.049, p=0.44
lymphatic system, n=148 , r= 0.059, p=0.48
peripheral nervous system, n=28 , r=−0.136, p=0.49
bladder, n=190 , r=−0.046, p=0.53
mesenchymal stem cell, n=10 , r=−0.223, p=0.54
gum, n=4 , r= 0.442
circulating reticulocyte, n=30 , r=−0.104, p=0.58
musculoskeletal system, n=17 , r= 0.140, p=0.59
bone marrow, n=8 , r= 0.186, p=0.66
endocrine system, n=140 , r= 0.037, p=0.66
heart, n=234 , r=−0.021, p=0.75
skin, n=15 , r= 0.061, p=0.83
hair follicle, n=16 , r=−0.048, p=0.86
blood unspecified leukocyte, n=35 , r= 0.031, p=0.86
salivary gland, n=14 , r=−0.037, p=0.9

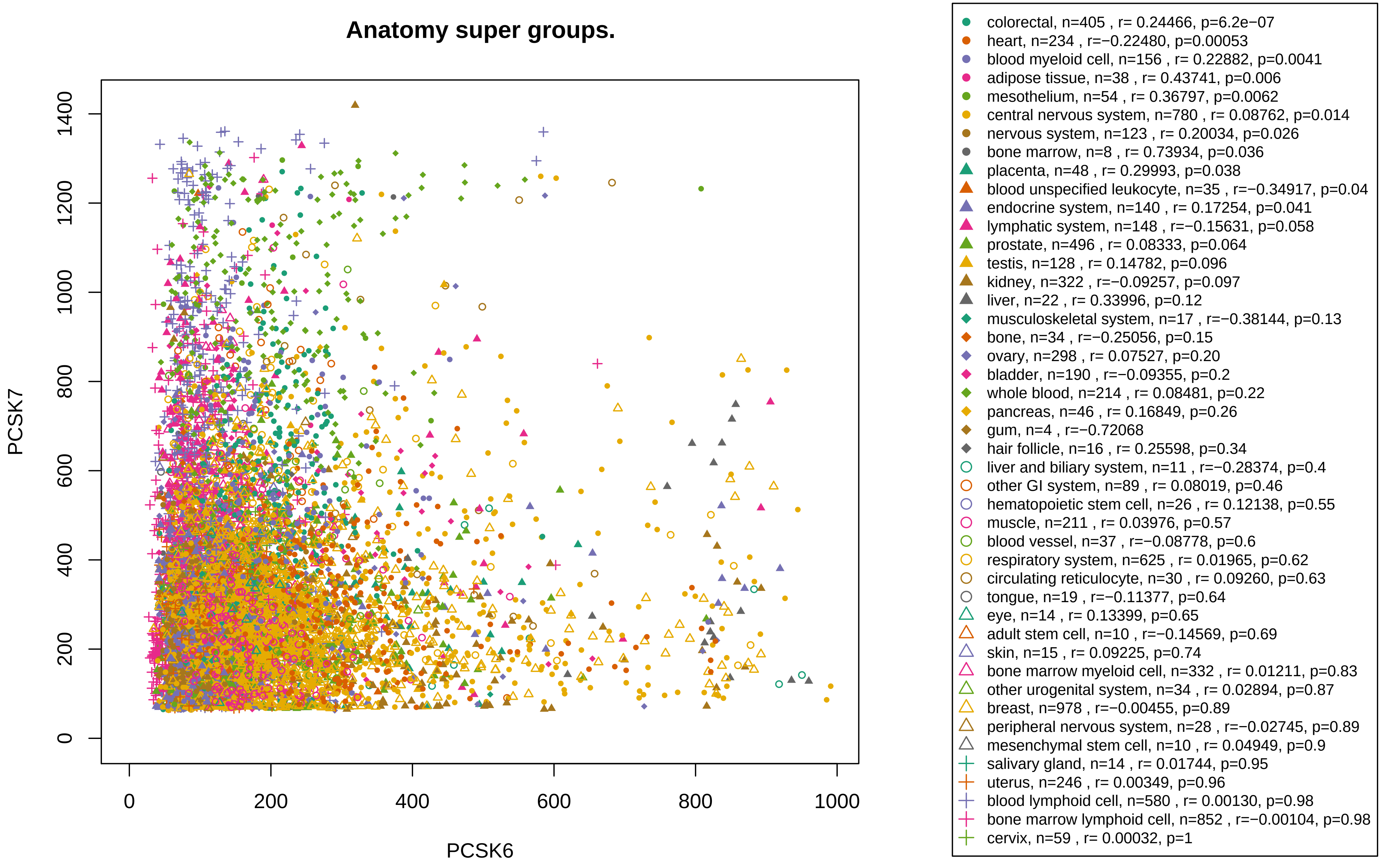
Anatomy super groups.
PCSK7
PCSK6
0
200
400
600
800
1000
1200
1400
colorectal, n=405 , r= 0.24466, p=6.2e−07
heart, n=234 , r=−0.22480, p=0.00053
blood myeloid cell, n=156 , r= 0.22882, p=0.0041
adipose tissue, n=38 , r= 0.43741, p=0.006
mesothelium, n=54 , r= 0.36797, p=0.0062
central nervous system, n=780 , r= 0.08762, p=0.014
nervous system, n=123 , r= 0.20034, p=0.026
bone marrow, n=8 , r= 0.73934, p=0.036
placenta, n=48 , r= 0.29993, p=0.038
blood unspecified leukocyte, n=35 , r=−0.34917, p=0.04
endocrine system, n=140 , r= 0.17254, p=0.041
lymphatic system, n=148 , r=−0.15631, p=0.058
prostate, n=496 , r= 0.08333, p=0.064
testis, n=128 , r= 0.14782, p=0.096
kidney, n=322 , r=−0.09257, p=0.097
liver, n=22 , r= 0.33996, p=0.12
musculoskeletal system, n=17 , r=−0.38144, p=0.13
bone, n=34 , r=−0.25056, p=0.15
ovary, n=298 , r= 0.07527, p=0.20
bladder, n=190 , r=−0.09355, p=0.2
whole blood, n=214 , r= 0.08481, p=0.22
pancreas, n=46 , r= 0.16849, p=0.26
gum, n=4 , r=−0.72068
hair follicle, n=16 , r= 0.25598, p=0.34
liver and biliary system, n=11 , r=−0.28374, p=0.4
other GI system, n=89 , r= 0.08019, p=0.46
hematopoietic stem cell, n=26 , r= 0.12138, p=0.55
muscle, n=211 , r= 0.03976, p=0.57
blood vessel, n=37 , r=−0.08778, p=0.6
respiratory system, n=625 , r= 0.01965, p=0.62
circulating reticulocyte, n=30 , r= 0.09260, p=0.63
tongue, n=19 , r=−0.11377, p=0.64
eye, n=14 , r= 0.13399, p=0.65
adult stem cell, n=10 , r=−0.14569, p=0.69
skin, n=15 , r= 0.09225, p=0.74
bone marrow myeloid cell, n=332 , r= 0.01211, p=0.83
other urogenital system, n=34 , r= 0.02894, p=0.87
breast, n=978 , r=−0.00455, p=0.89
peripheral nervous system, n=28 , r=−0.02745, p=0.89
mesenchymal stem cell, n=10 , r= 0.04949, p=0.9
salivary gland, n=14 , r= 0.01744, p=0.95
uterus, n=246 , r= 0.00349, p=0.96
blood lymphoid cell, n=580 , r= 0.00130, p=0.98
bone marrow lymphoid cell, n=852 , r=−0.00104, p=0.98
cervix, n=59 , r= 0.00032, p=1
