# Supplementary material for: Identification of proprotein convertase substrates using genome-wide expression correlation analysis
Source: BMC Genomics. 2011 Dec 20;12:618. doi: 10.1186/1471-2164-12-618 (PMC3258279; doi:10.1186/1471-2164-12-618)
Supplement: Additional file 6 — Chromosomal clustering of putative PCSK targets. [file 1471-2164-12-618-S6.PDF]

Supplementary Data 6. Chromosomal clusters of putative PCSK targets found.

| | chr | begin | end | genes | p value |
|---|---|---|---|---|---|
| **PCSK1** | 1 | 202852927 | 203837960 | MFSD4, NFASC, LRRN5 | 1.46e-03 |
| **PCSK1** | 2 | 219862590 | 224175365 | PTPRN, EPHA4, SCG2 | 1.45e-04 |
| **PCSK1** | 5 | 140286485 | 140586042 | PCDHAC1, PCDHB10, PCDHB14 | 1.33e-03 |
| **PCSK1** | 5 | 19508913 | 24680668 | CDH18, CDH12, CDH10 | 1.33e-03 |
| **PCSK1** | 12 | 69046328 | 71345688 | TRHDE, KCNMB4, PTPRR | 1.38e-04 |
| **PCSK1** | 13 | 83349344 | 96289812 | HS6ST3, SLITRK5, SLITRK1 | 2.44e-03 |
| **PCSK2** | 4 | 45946462 | 47123202 | GABRA2, GABRA4, GABRB1 | 3.70e-04 |
| **PCSK2** | 5 | 19508913 | 24680668 | CDH18, CDH12, CDH10 | 1.81e-03 |
| **PCSK2** | 13 | 83349344 | 96289812 | HS6ST3, SLITRK5, SLITRK1, GPC5 | 2.12e-04 |
| **furin, gpc** | 6 | 31799139 | 32021427 | C6orf25, NEU1, C2 | 8.00e-04 |
| **PCSK4** | 1 | 20832534 | 22802674 | EPHA8, PINK1, ELA3A | 2.52e-04 |
| **PCSK5** | 6 | 26473376 | 26561621 | BTN3A2, BTN3A1, BTN3A3 | 2.43e-04 |
| **PCSK7** | 6 | 26473376 | 26561621 | BTN3A2, BTN3A1, BTN3A3 | 8.00e-04 |
| **PCSK7** | 11 | 60495749 | 60970803 | CD6, FLJ20487, CD5 | 4.29e-04 |
| **PCSK7** | 11 | 117605546 | 117718669 | CD3E, LOC196264, CD3D | 4.29e-04 |
| **PCSK7** | 14 | 69148065 | 70651852 | SLC8A3, PCNX, KIAA0247 | 1.15e-03 |
| **PCSK7** | 16 | 28850760 | 29664841 | SPN, CD19, C16orf54 | 7.57e-04 |
| **furin f, PCSK6** | no clusters found | | | | |

Furin f = furin-specific cleavage site

Furin gpc = general PCSK cleavage site
