# Supplementary figures and images for: Identification of proprotein convertase substrates using genome-wide expression correlation analysis
Source: BMC Genomics. 2011 Dec 20;12:618. doi: 10.1186/1471-2164-12-618 (PMC3258279; doi:10.1186/1471-2164-12-618)

PCSK1

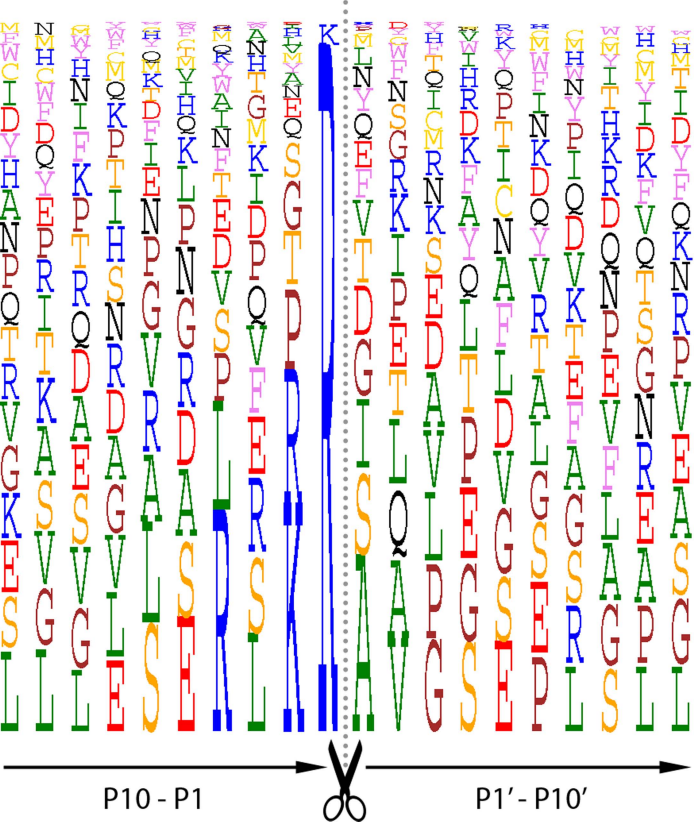

PCSK2

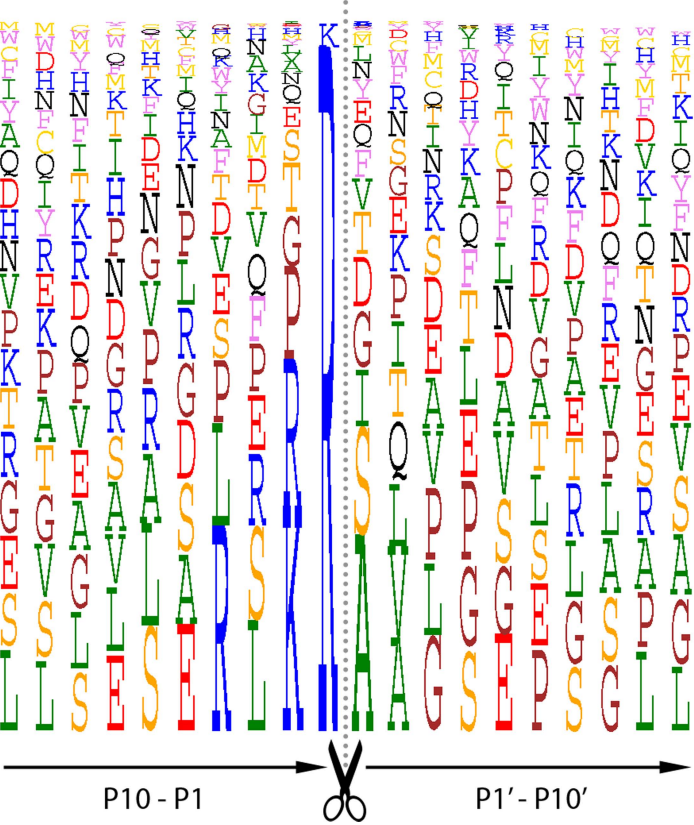

PCSK4

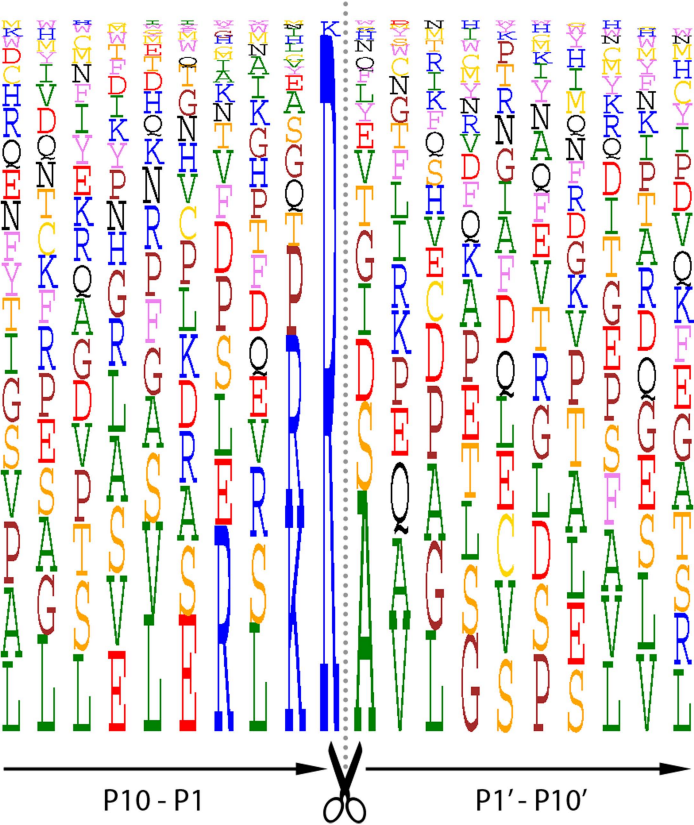

PCSK5

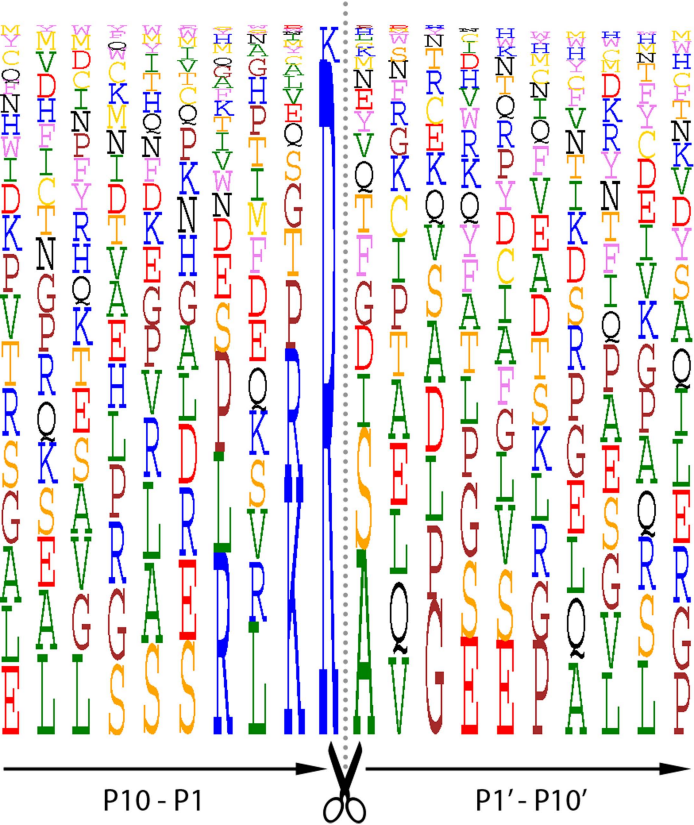

PCSK6

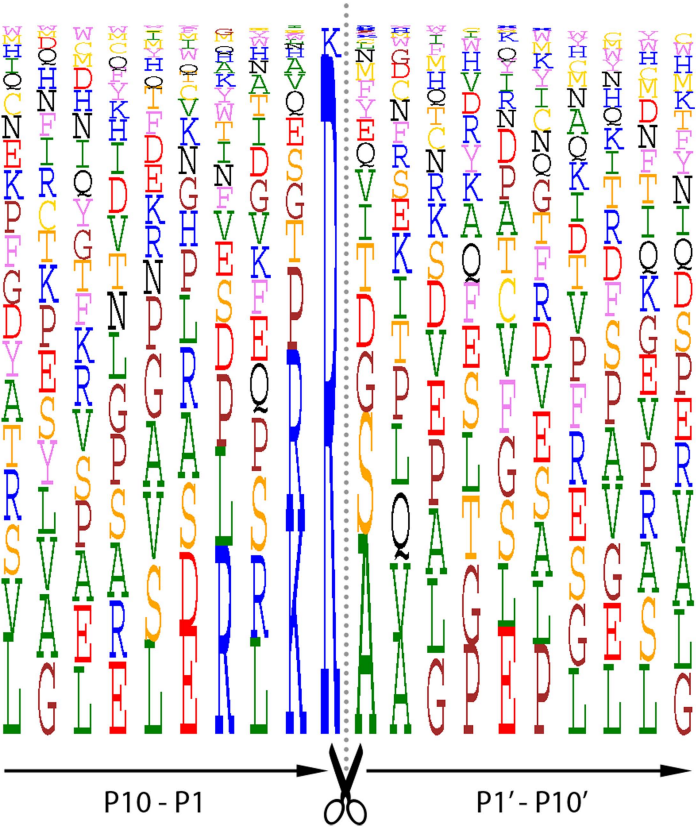

PCSK7

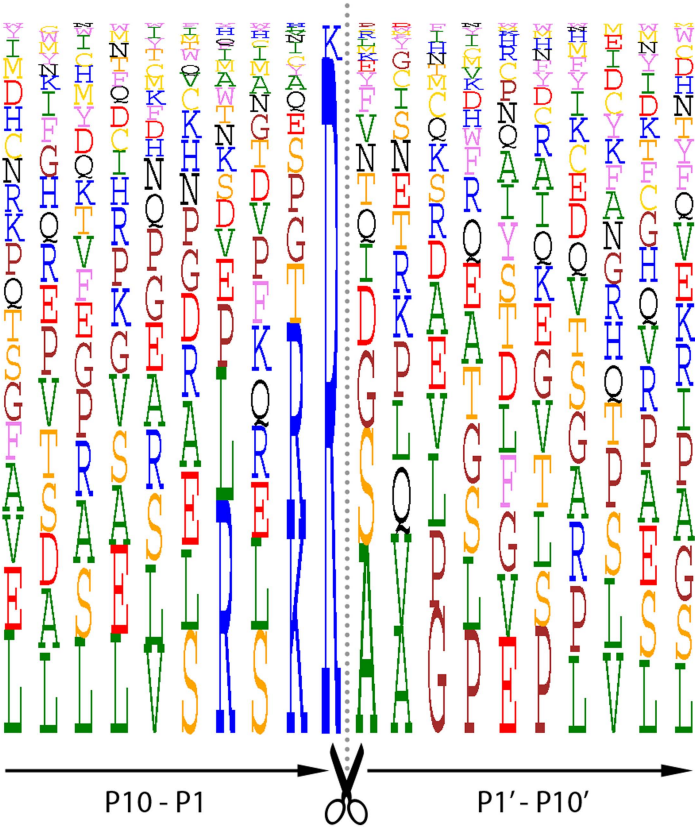

FURIN

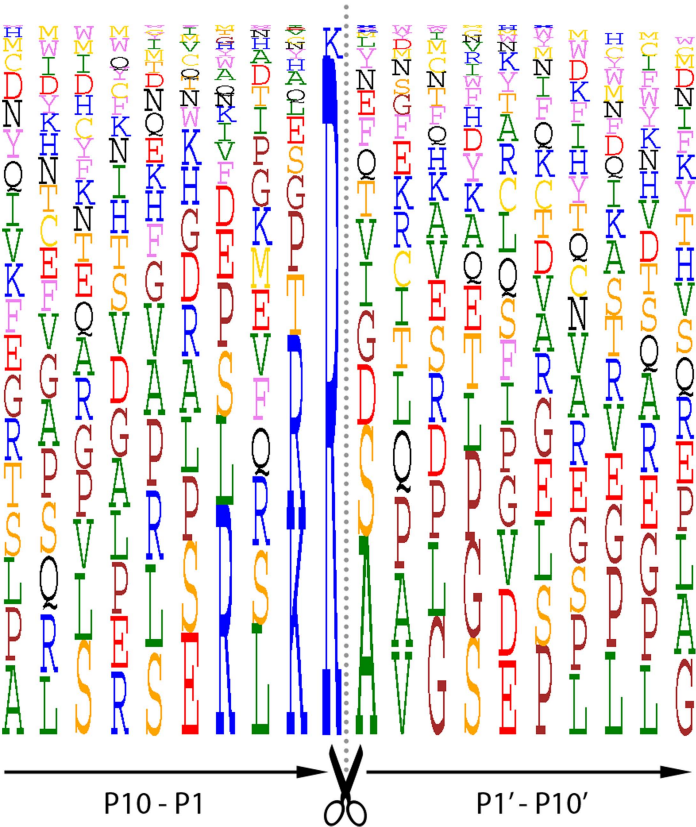

FURIN (furin-specific prediction)

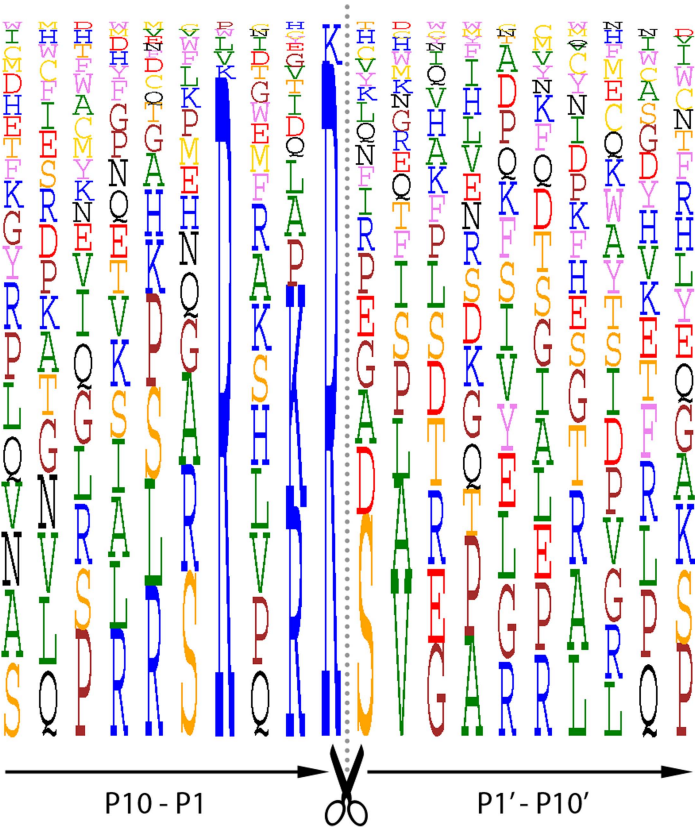

Supplement: Additional file 7 — MultiDisp figures of potential PCSK target sequences. The sequences were predicted from the group of top-correlated gene translations using the general PC prediction and signal peptide prediction methods on the ProP 1.0 server (http://www.cbs.dtu.dk/services/ProP/). Potential target peptides from signal peptide-containing sequences were restricted to ten residues upstream (P10-P1) and downstream (P1'-P10') of the predicted cleavage site. Amino acid compositions at each site in the groups of potential targets for each PCSK were plotted using MultiDisp (http://bioinf.uta.fi/cgi-bin/MultiDisp.cgi) that scales the character heights based on amino acid frequency. Scissors and dotted line mark the predicted cleavage site. [file 1471-2164-12-618-S7.PDF]

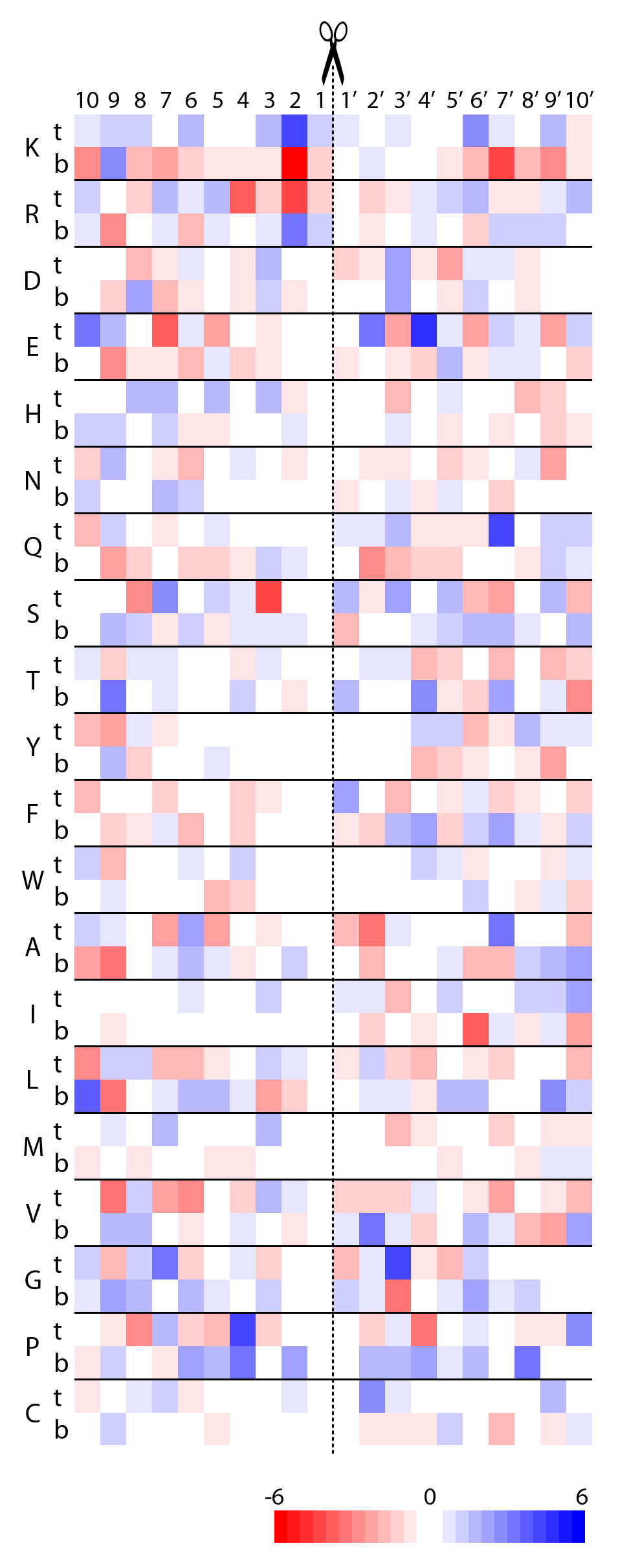

Supplement: Additional file 8 — Amino acid occurrence of putative target molecules for PCSK5. Twenty residues (P10 - P10', marked with numbers 10 - 10') around the PCSK cleavage sites of highly correlating genes have been plotted for PCSK5. Top (t) and bottom (b) groups are shown for each amino acid type. Blue color indicates increased occurrence of a particular amino acid residue type in certain position of the putative substrate when all PCSKs are considered, whereas red colors mean low occurrence of a specific amino acid. White indicates an average occurrence frequency of a specific amino acid. The increase or decrease in occurrence is shown as a scale of percentages and a combined data containing all PCSKs has been used as a comparison point. The scaling (-6 percentage to +6 percentage) is shown as a color gradient below the figure. The potentially scissile bond P1-P1' is marked with scissors and dashed line. [file 1471-2164-12-618-S8.TIFF]
